# BoltzOmics: Predicting genetic variant effects on drug binding with Boltz-2

**DOI:** 10.1016/j.isci.2026.116797

**Published:** 2026-07-16

**Authors:** Khoa Ngo, Kermit L. Carraway, Colleen E. Clancy, Hajar Amini

**Affiliations:** 1 Center for Precision Medicine and Data Sciences, School of Medicine, University of California, Sacramento, CA 95817, USA; 2 Department of Physiology and Membrane Biology, University of California, Davis, 1 Shields Avenue, Davis, CA 95616, USA; 3 Department of Biochemistry and Molecular Medicine, University of California, Davis, 1 Shields Avenue, Davis, CA 95616, USA; 4 Department of Pharmacology, University of California, Davis, 1 Shields Avenue, Davis, CA 95616, USA

**Keywords:** deep learning, protein structure prediction, drug binding, genetic variants, precision medicine, computational drug discovery

## Abstract

A mechanistic understanding of how genetic variants alter drug-receptor binding is central to precision medicine, drug response prediction, and drug development. Yet, experimental mutation-drug profiling remains slow and expensive, while existing computational approaches often trade accuracy for scalability. We developed BoltzOmics, an interactive, open-source platform that integrates Boltz-2, a deep learning model for biomolecular structure prediction, to rapidly assess mutation effects on drug binding. Starting from amino acid sequences, the workflow queries databases for genetic variants, generates wild-type and mutant protein structures, and screens multiple drugs across variants to predict binding affinity changes. We evaluated BoltzOmics across four targets: hERG, Na_V_1.5, HER2, and CYP3A4. Predictions achieved Pearson correlations with experimental drug IC_50_ data up to 0.76 for wild-type proteins and 0.60 for mutants. By enabling scalable, high-throughput assessment of drug-variant interactions, BoltzOmics establishes a practical AI-driven framework for accelerating computational drug discovery and advancing precision medicine research.

## Highlights

• BoltzOmics uses Boltz-2 to reveal variant-linked shifts in predicted drug potency
• BoltzOmics enables accessible and efficient screening of variant drug responses
• Drug-bound structures support predicted protein-ligand interaction poses
• Boltz-2 shows stronger agreement than tested docking and affinity predictors

## Graphical abstract

**Figure:**
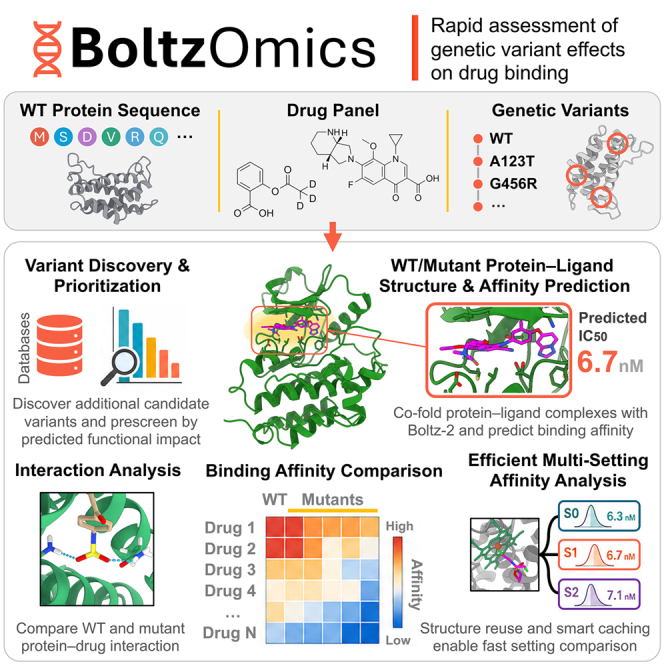

## Teaser

Artificial intelligence; Biological sciences; Machine learning; Protein structure aspects; Structural biology

## Introduction

One of the most pressing challenges in modern medicine^1^^,^^2^^,^^3^ has been to reveal the relationship between genetic variation and drug response. Addressing this challenge will allow the arrival of a true era of personalized therapies and improved prediction of adverse drug effects in individuals. Genetic variants that result in amino acid changes can dramatically alter protein structure and function, leading to changes in drug binding affinity that may result in therapeutic failure, adverse reactions, or drug resistance.^4^^,^^5^^,^^6^ Despite the recognition of this persistent pharmacogenomic challenge, it remains difficult to predict at scale how specific variants affect target protein-drug interactions, creating an obstacle to patient-specific precision medicine and rational design and selection of drugs.

Current approaches to characterizing mutation-drug interactions rely on experimental methods that, while useful, are resource-intensive and time-consuming.^7^ High-throughput screening of mutant proteins requires specialized facilities, extensive protein engineering, and months of laboratory work to generate comprehensive datasets. This experimental bottleneck has created a critical gap between the ever-growing list of genetic variants through large-scale sequencing efforts^8^^,^^9^ and our understanding of their functional consequences for drug therapy.

Computational methods have emerged as promising alternatives for predicting mutation effects on drug binding, offering the potential for rapid, cost-effective screening of genetic variants.^10^ Among these, physics-based approaches such as molecular dynamics simulations^11^^,^^12^^,^^13^ provide detailed mechanistic and kinetic insights, but these techniques remain computationally demanding and difficult to scale due to the extensive sampling required to traverse complex conformational landscapes.^14^

Structure-based docking algorithms^15^^,^^16^^,^^17^^,^^18^ can generate candidate binding poses and relative rankings across compounds, but docking accuracy is sensitive to scoring function limitations, and they often require extensive system preparation, protocol tuning, and post-processing to yield interpretable results. In addition, docking methods may produce poor affinity estimates in realistic settings.^19^^,^^20^^,^^21^ In parallel, neural network-based drug-target affinity (DTA) models^22^^,^^23^ can rapidly map drug and protein representations to predicted interaction strength without explicit 3D docking. However, these models often perform well only on biased test sets and lack the explicit structural context needed for variant-dependent binding generalization.^24^

Together, the limitations of existing approaches highlight the need for workflows that combine structural insight with practical scalability for large variant and drug panels. Boltz-2 helps address part of this challenge by jointly predicting protein-ligand structure and binding affinity within a single inference framework, reducing reliance on complex docking pipelines or purely sequence-based predictions. BoltzOmics extends this function into a practical screening system by organizing variant discovery and prioritization, reusing intermediate artifacts such as sequence alignments and predicted structures across related runs, and providing integrated visualization and comparison tools. This design reduces repetitive setup and computation while making large protein-drug screening tasks easier to run, interpret, and reproduce across targets.

The recent revolution in protein structure prediction, driven by deep learning models like AlphaFold and its successors,^25^^,^^26^^,^^27^ has fundamentally transformed our ability to rapidly generate accurate structural models. Boltz-2, representing the latest generation of these approaches, can predict protein structures with remarkable accuracy in minutes rather than the days or weeks required by traditional methods.^28^ This breakthrough creates an unprecedented opportunity to leverage high-quality structural predictions for drug discovery applications, potentially bridging the gap between computational efficiency and prediction accuracy. Benchmark studies indicate that Boltz-2 can estimate binding affinities with performance comparable to, or better than, a variety of physics-based and machine-learning-based methods, on a far shorter timescale.^28^ Despite its promise, the efficacy of Boltz-2 in modeling the effects of genetic variants and resulting amino acid changes on drug binding remains largely unexplored.

The clinical significance of genetic variant-drug interactions varies dramatically across therapeutic areas and protein targets. Cardiac ion channels such as hERG and Na_V_1.5 are critical for drug safety assessment, as variants in these proteins can lead to life-threatening arrhythmias when combined with certain medications. The hERG channel has been responsible for numerous drug withdrawals due to its role in drug-induced long QT syndrome,^29^^,^^30^^,^^31^ while Na_V_1.5 variants cause inherited arrhythmias like Brugada syndrome that can be exacerbated by sodium channel-blocking drugs.^32^^,^^33^ Oncogenic proteins such as the human epidermal growth factor receptor 2 (HER2) kinase domain are targets where mutation-driven drug resistance represents a major clinical challenge, with specific variants conferring resistance to targeted therapies like afatinib and lapatinib.^34^^,^^35^ Meanwhile, metabolic enzymes such as cytochrome P450 3A4 (CYP3A4) present different challenges, as this enzyme metabolizes approximately 50% of clinically used drugs, and genetic polymorphisms can dramatically affect drug clearance and efficacy.^36^^,^^37^

Despite growing recognition of the importance of how genetic differences affect drug response, both experimental and computational screening of genetic variants remain time- and resource-intensive, making it impractical to comprehensively evaluate the full spectrum of clinically relevant variants. As a result, drug development and clinical decision making often rely on generalized, one-size-fits-all approaches that overlook individual variability. This disconnect contributes to suboptimal therapeutic outcomes, including adverse drug reactions, reduced efficacy, and the emergence of resistance, highlighting the urgent need for scalable, mutation-aware strategies that can be integrated into precision medicine pipelines.

Here, we demonstrate an approach to address these limitations by developing and validating an interactive pipeline that integrates Boltz-2 structure prediction to rapidly assess the effects of genetic variants on protein-drug binding. Our approach offers several key innovations: first, it utilizes state-of-the-art deep learning structure prediction for rapid, accurate modeling of both wild-type (WT) and mutant proteins^28^; second, it demonstrates generalizability across therapeutically diverse protein targets representing multiple structural classes and clinical applications; and third, it provides an interactive, accessible, open-source implementation that broadens access to sophisticated computational drug discovery methods.

We further evaluate both the potential and the limitations of deep learning-based mutation effect prediction across the commonly drugged protein targets: hERG, Na_V_1.5, CYP3A4, and HER2. Germline variants in ion channels such as hERG and Na_V_1.5 as well as in the metabolic enzyme CYP3A4 influence interindividual differences in drug metabolism, efficacy, and/or toxicity. In contrast, alterations in HER2 represent somatic mutations that drive tumorigenesis and therapeutic resistance in cancer. While previous studies have shown that Boltz-2 can achieve comparable accuracy to physics-based methods for WT protein structure prediction,^28^ our results extend this finding to how gene variants affect prediction, demonstrating meaningful correlations with experimental data. This advantage opens new possibilities for large-scale pharmacogenomic analysis and precision medicine applications that would be computationally intractable with traditional methods. The open-source nature of our pipeline facilitates broader adoption and validation, potentially accelerating the integration of computational methods into drug discovery and clinical practice.

## Results

### Interactive computational pipeline for screening drug effects on protein variants

**Figure 1:**
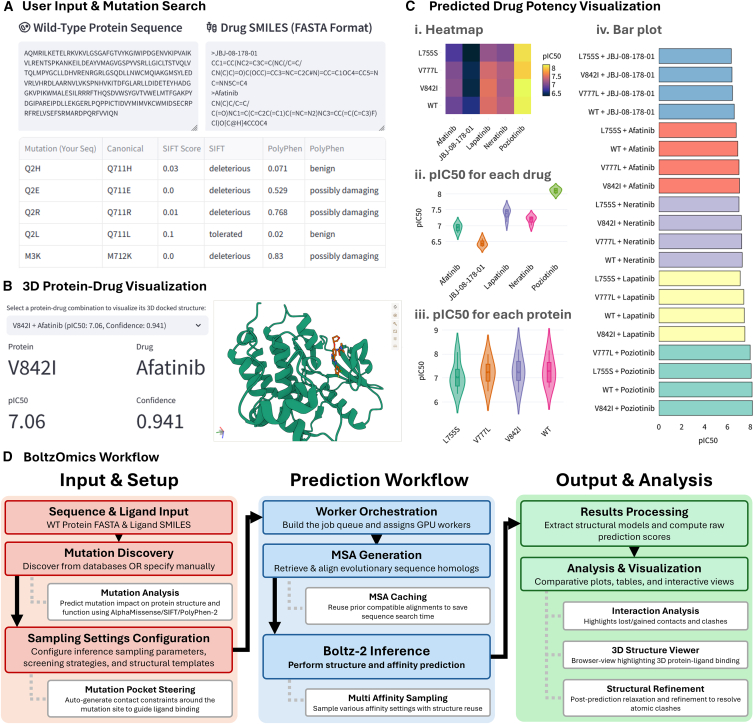
Overview of the open-source deep learning pipeline for mutation-drug interaction prediction (A) User input and mutation search: the interactive interface allows users to input wild-type (WT) protein sequences, customize computational parameters, and perform mutation searches that first query the WT sequence to identify the correct protein, then retrieve known mutations and associated *in silico* prediction scores from available databases. Candidate drugs can also be provided as SMILES strings, formatted in FASTA style with “>” as separators, to screen selected mutants for potential drug interactions. (B) 3D structural visualization: interactive molecular visualization displays predicted protein structures with bound ligand, along with predicted pIC_50_ and confidence (defined as 0.8 × pLDDT + 0.2 × ipTM). (C) Predicted drug potency visualization: multiple visualization formats present binding affinity predictions across protein variants and drug panels. (i) Heatmap displays pIC_50_ values for all protein-drug combinations with color-coded binding strengths. (ii) Violin plots show pIC_50_ distributions for individual drugs across all protein variants. (iii) Violin plots illustrate binding affinity ranges for each protein variant across the drug panel. (iv) Bar plot provides comparative pIC_50_ values for specific protein-drug combinations, enabling direct comparison between WT and mutant forms. The pipeline generates comprehensive binding affinity profiles in minutes, facilitating rapid pharmacogenomic screening and hypothesis generation for precision medicine applications. (D) BoltzOmics workflow. The pipeline is organized into three stages: input and setup, prediction workflow, and output and analysis. Input includes sequence and ligand specification, mutation discovery and prioritization, and configurable sampling strategies. The prediction stage extends vanilla Boltz-2 inference with multiple mechanistic additions focusing on efficiency, including MSA caching, affinity multi-sampling with structure reuse, and mutation-conditioned pocket steering. The output stage integrates post-inference structural extraction, interaction comparison, optional structure refinement, and ranked data export.

The drug screening pipeline comprises a comprehensive software framework, called BoltzOmics, with a fully interactive graphical interface, allowing users to carry out advanced pharmacogenomic analyses without requiring programming expertise (Figure 1).

The workflow begins with the user providing a WT protein sequence. The tool then queries public databases to identify the closest protein match, retrieves known gene variants, and annotates their potential functional impact using established resources such as Sorting Intolerant From Tolerant (SIFT)^38^ and PolyPhen-2,^39^ which assess the likelihood that amino acid substitutions disrupt protein function. To further support mutation prioritization, the platform also incorporates machine-learning-based predictors such as AlphaMissense,^40^ which leverages deep learning to estimate the pathogenicity of missense variants across the genome based on large-scale evolutionary and structural patterns. Together, these complementary annotations help identify the variants most likely to influence protein behavior or drug response, streamlining selection of high-impact mutations for downstream structural and affinity screening. Alternatively, if specific gene variants are already known, users can enter them directly. Next, the user supplies a list of drug compounds represented as SMILES strings (simplified molecular input line entry system, a text-based molecular representation).

The deep learning model Boltz-2 then performs large-scale screening of each protein variant against each compound, predicting 3D structures of WT and mutant proteins in complex with drugs and estimating binding potency (IC_50_, half-maximal inhibitory concentration). Finally, the tool generates visualizations of binding profiles across all drug-protein combinations, allowing pharmacogenomic analysis of genetic variants in a systematic and scalable manner. Optionally, for added comprehensiveness, users may also provide a list of proteins in FASTA format to evaluate binding of each ligand across multiple targets.

The tool supports multiple operational modes in addition to core screening approaches (Figure 1A). Users can incorporate ligand cofactors essential for protein function, utilize existing structural files as templates to guide prediction accuracy, and specify binding pocket residues to steer ligand docking toward specific sites within complex protein structures. In addition, users can add post-translational modifications that may affect drug binding.

Deep learning Boltz-2 prediction parameters are fully customizable to optimize the balance between accuracy and computational efficiency. Recycling steps control the number of iterative refinement cycles used to improve prediction accuracy, with higher values enhancing structural quality at the cost of increased computation time. Sampling steps determine the number of conformations sampled from the model distribution, while diffusion samples control prediction reliability through ensemble averaging. The system also enables customization of multiple sequence alignments (MSAs) usage and generation, allowing users to include evolutionary information for improved structural predictions.

BoltzOmics is designed to make large-scale variant-drug screening more practical and reproducible by adding workflow steps that are not part of a standard Boltz-2 run. Beyond single protein-ligand inference, the platform supports variant discovery and mutation prioritization before prediction, allowing users to identify and triage candidate variants for screening. During batch screening, BoltzOmics reduces repeated preprocessing by caching compatible MSAs across related WT and mutant jobs when possible, with fallback to fresh generation if reuse fails. It also separates structural prediction from affinity sensitivity analysis: structure is generated once, and the affinity stage can then be reevaluated across multiple sampling settings while reusing the same structural output. This allows users to inspect setting dependence without rerunning the full structure-plus-affinity pipeline for every condition. Beyond prediction, the workflow integrates downstream structural interpretation, including protein-ligand interaction analysis with Protein-Ligand Interaction Profiler (PLIP)^41^ and optional OpenMM-based refinement to resolve local steric clashes.^42^ Together, these additions turn Boltz-2 from a single-query predictor into a mutation-screening workflow that supports variant selection, repeated execution across many mutants and drugs, setting-aware affinity analysis, and integrated structural interpretation.

The integrated 3D molecular viewer allows real-time exploration of predicted protein-ligand complexes with downloadable structural files in standard formats (Figure 1B). This comprehensive implementation makes gene variant effect prediction accessible to researchers while maintaining the flexibility required for specialized applications in drug discovery and precision medicine. The visualization suite provides multiple complementary views of binding affinity data, including interactive heatmaps displaying pIC_50_ values, defined as −log_10_(IC_50_ in molar units), across all protein-drug combinations (Figure 1C i); violin plots showing binding affinity distributions for individual compounds (Figure 1C ii) and protein variant effects on drug binding (Figure 1C iii); and comparative bar plots enabling direct WT versus mutant comparisons (Figure 1C iv). A dedicated workflow diagram (Figure 1D) summarizes the full end-to-end usage pattern: mutation discovery and prioritization, WT-first batching across variants, structure generation, affinity estimation with optional multi-sampling, and downstream comparison and visualization outputs. Figure S1 further illustrates how these capabilities are exposed in the interface, including runtime-saving multi-setting affinity analysis, optional post-prediction refinement, and integrated interaction summaries. While BoltzOmics relies on pretrained Boltz-2 weights for structure and affinity prediction, it introduces a mutation-oriented orchestration layer that governs how inference is prepared, executed, aggregated, and interpreted across WT-mutant-drug combinations.

**Figure 2:**
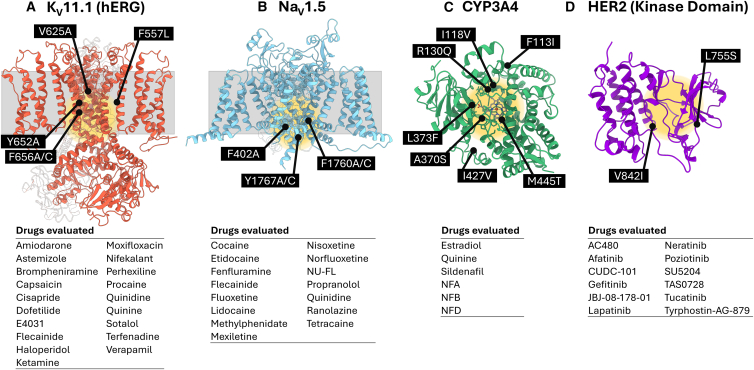
Target proteins, drugs tested, and mutation locations for pharmacogenomic benchmarking Structural representations of the four therapeutically diverse protein targets evaluated in this study, showing the locations of clinically relevant mutations tested and common drug binding sites (highlighted in yellow), along with a list of drugs assessed for each target. (A) hERG potassium channel (red) with mutations V625A, F557L, Y652A, and F656A/C located within the pore domain that are critical for channel gating and drug binding. (B) Na_V_1.5 sodium channel (blue) displays mutations F402A, F1760A/C, and Y1767A/C positioned in the pore domain where sodium channel blockers typically interact. (C) CYP3A4 metabolic enzyme (green) with mutations A370S, F113I, I118V, I427V, L373F, M445T, and R130Q distributed around the active site cavity where substrate binding and metabolism occur. (D) HER2 kinase domain (purple) showing mutations L755S and V842I within the ATP-binding pocket (highlighted in yellow) where kinase inhibitors compete with ATP for binding.

To evaluate the predictive performance and validate the clinical utility of the pipeline, we performed comprehensive benchmarking across four therapeutically diverse drug targets representing key protein classes in pharmacology and drug safety assessment (Figures 2A–2D). These targets include cardiac ion channels essential for drug safety evaluation (hERG and Na_V_1.5), drug-metabolizing enzymes crucial for pharmacokinetics (CYP3A4), and oncogenic kinases central to cancer therapeutics (HER2). The mutation set comprises both naturally occurring variants and experimentally characterized substitutions commonly used to probe drug binding mechanisms. All selected mutations were located within or near known drug-binding sites, enabling a focused evaluation of the pipeline's accuracy to model mutation-induced changes in drug-protein interactions. While mutations outside these regions can also influence drug response, they may do so through complex signaling cascades, long-range allosteric effects, or stability changes. These mechanisms fall outside the scope of current structural modeling approaches and will be explored in future studies. All experimental IC_50_ (or related potency/activity) values used for benchmarking were curated from peer-reviewed publications reporting direct measurements (e.g., electrophysiology-derived IC_50_ for ion channels and biochemical inhibition/activity assays for enzymes/kinases). Source references for each variant-drug data point are provided in Tables S1–S4.

### K_V_11.1 (hERG) cardiac potassium channel

The K_V_11.1, also known as hERG (encoded by the human *Ether-à-go-go*-related gene), is a potassium channel that represents a critical target for drug safety assessment. Drug-induced hERG blockade can lead to life-threatening cardiac arrhythmias and has been responsible for numerous drug market withdrawals.^29^^,^^30^^,^^43^ We evaluated our Boltz-2-based pipeline against experimental pIC_50_ data across multiple hERG variants and a panel of 19 drugs, including known hERG blockers and drugs with varying cardiac risk profiles. Experimental and predicted values were compared on the pIC_50_ scale, defined as pIC_50_ = −log_10_(IC_50_ in M). This logarithmic transformation makes very strong and very weak binders easier to compare on the same scale and improves interpretability of correlations between predicted and experimental values.

To examine how increasing affinity sampling rigor affects predictive results, we evaluated Boltz-2 under three affinity sampling settings (settings 0, 1, and 2), where setting 0 corresponds to the Boltz-2 default. These settings control the extent of affinity refinement: “sampling steps” define the number of diffusion refinement steps per affinity estimate, and “diffusion samples” specifies how many independent affinity runs are ensemble-averaged. Higher settings, therefore, apply deeper refinement and broader averaging. Specifically, setting 0 uses 200 steps and 5 samples, setting 1 uses 300 steps and 7 samples, and setting 2 uses 400 steps and 9 samples.

**Figure 3:**
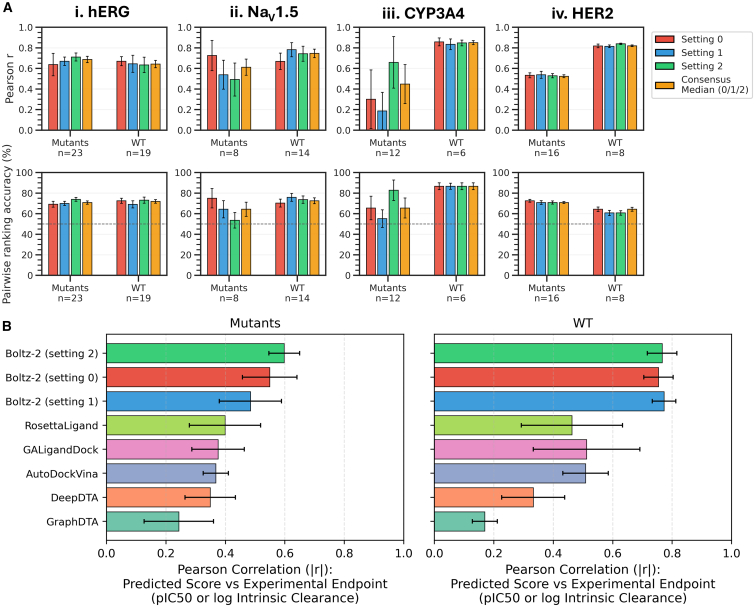
Summary of Boltz-2 prediction performance and comparison with alternative computational approaches (A) Performance of Boltz-2 across four protein targets (hERG, NaV1.5, CYP3A4, and HER2) for both mutant variants and WT proteins under three affinity sampling settings (settings 0–2), together with a consensus median across all settings. The top row shows Pearson correlation coefficients (r) between predicted binding scores and experimental measurements (pIC_50_ or log intrinsic clearance). The bottom row shows pairwise affinity ranking accuracy, defined as the percentage of correctly ordered drug-protein pairs relative to experimental values (e.g., if 10 comparable pairs are evaluated and 7 have matching predicted and experimental binding affinity order, the pairwise ranking accuracy is 70%). The dashed line indicates 50% random baseline. Sample sizes for each dataset are indicated below the *x* axis labels. The consensus median reflects the median of values obtained across all 3 sampling settings. Data are represented as the mean correlation across targets, and error bars indicate standard errors of the mean. (B) Cross-method performance comparison summarized as mean absolute Pearson correlation across all targets for mutant and WT datasets. Methods include Boltz-2 (three sampling settings), structure-based docking approaches (GALigandDock, RosettaLigand, and AutoDock Vina), and sequence-based deep learning drug-target affinity models (DeepDTA and GraphDTA). Data are represented as the mean correlation across targets, and error bars indicate standard errors of the mean. Boltz-2 consistently shows the strongest agreement with experimental measurements across both WT and mutant panels.

Across three different sampling settings, the deep learning approach demonstrated consistently moderately-strong to strong predictive performance, with Pearson correlation coefficients (r) ranging from 0.63 to 0.71 depending on the protein variant and sampling parameters (Figure 3A i, with detailed scatterplots in Figure S2). All experimental data^43^^,^^44^^,^^45^^,^^46^^,^^47^^,^^48^^,^^49^^,^^50^^,^^51^^,^^52^^,^^53^^,^^54^^,^^55^^,^^56^^,^^57^^,^^58^^,^^59^^,^^60^^,^^61^^,^^62^^,^^63^^,^^64^^,^^65^^,^^66^^,^^67^ used for comparison are recorded in Table S1. WT hERG showed moderately-strong correlations (r = 0.63–0.67) with relatively low root-mean-square error (RMSE = 0.72–0.74, measuring average pIC_50_ deviation from experimental values along the 1:1 line), indicating reliable absolute binding affinity predictions. Interestingly, mutant variants showed stronger correlations with experimental data (r = 0.64–0.71) than the WT, suggesting the model is especially good at capturing differences in binding across mutant forms, even though the higher RMSE values (1.20–1.27) point to lower accuracy in absolute predictions. WT had slightly lower correlations; while seemingly counterintuitive, it can be in part attributed to experimental data being aggregated from numerous independent studies using diverse assay formats, which introduces variability and statistical noise. Pairwise ranking accuracy remained consistently above random across all sampling settings (Figure 3A i). Here, drugs are ranked by predicted pIC_50_ and compared against experimental pIC_50_ rankings within each protein-mutant context; each unordered drug pair is counted as correct when the predicted and experimental order agree. This shows that the model reliably preserves which compounds are relatively stronger vs. weaker binders, even when absolute pIC_50_ values shift across settings.

The approach showed consistent performance across different sampling settings, with minimal variation in prediction errors between settings for most compounds (Figure S3). Certain drugs, including haloperidol and sotalol, showed larger deviations from experimental values, while others, such as amiodarone and cisapride, demonstrated more consistent predictions across all variants. The model maintained consistent relative rankings across different hERG variants suggesting utility for comparative binding affinity assessments in pharmacogenomic applications, where understanding relative mutation effects may be more critical than absolute binding predictions.

### Na_V_1.5 cardiac sodium channel

Encoded by the *SCN5A* gene, Na_V_1.5 represents the predominant voltage-gated sodium channel in cardiac tissue, with germline variants causing inherited arrhythmia syndromes including Brugada syndrome and long QT syndrome.^33^ Understanding how genetic variants affect drug interactions with Na_V_1.5 is crucial for both avoiding drug-induced arrhythmias^68^^,^^69^^,^^70^^,^^71^ and other side effects^32^^,^^72^ and optimizing antiarrhythmic therapy.^32^ We evaluated our pipeline against experimental data for WT Na_V_1.5 and five variants (F1760A, F1760C, F402A, Y1767A, and Y1767C) by using a panel of 15 drugs including sodium channel blockers, local anesthetics, and antiarrhythmics.

Boltz-2 predictions showed variable performance across different protein variants and sampling settings (Figure 3A ii; Figure S4). All experimental data^73^^,^^74^^,^^75^^,^^76^^,^^77^^,^^78^^,^^79^^,^^80^^,^^81^^,^^82^^,^^83^ used for comparison are shown in Table S2. In Figure S4, WT Na_V_1.5 demonstrated the strongest correlations with experimental data, achieving r = 0.67–0.74 (R^2^ = 0.45–0.61) across the three sampling conditions. In contrast, mutant variants showed more varied performance (r = 0.49–0.72), depending on the settings. Although statistical significance (*p* values) was generally lower for mutants due to the limited availability of experimental data, the observed trends still suggest meaningful capture of variant-specific effects. Pairwise ranking accuracy remained above the random baseline across all settings (Figure 3A ii), with WT performance improving modestly at higher settings while mutant ranking was more variable, consistent with the greater conformational complexity of Na_V_1.5.

Drug-specific prediction patterns revealed several differences in model accuracy across compound classes (Figure S5). Local anesthetics, such as lidocaine and cocaine, showed relatively consistent predictions across all variants and sampling conditions. Antiarrhythmic drugs, including quinidine, flecainide, and propranolol, demonstrated variable performance, with some compounds, such as mexiletine, showing larger prediction errors in certain mutant backgrounds. Notably, the model maintained consistent relative performance rankings across different sampling parameters, suggesting robustness in the underlying structure-activity relationships regardless of absolute prediction variations. The superior performance observed for WT Na_V_1.5 compared with mutant variants may reflect the model’s training advantage on WT structures, highlighting the importance of variant-specific validation in clinical applications.

### Cytochrome P450 3A4 (CYP3A4)

CYP3A4 represents the most clinically significant drug-metabolizing enzyme, responsible for the breakdown of nearly 50% of marketed pharmaceuticals.^37^^,^^84^ Genetic polymorphisms in CYP3A4 can dramatically alter drug clearance rates, leading to therapeutic failures or adverse drug reactions.^37^^,^^85^^,^^86^ Unlike binding affinity measurements used for the previous targets, CYP3A4 evaluation required comparison against experimental intrinsic clearance data, presenting a fundamentally different prediction challenge. Predicted pIC_50_ and experimental intrinsic clearance probe different aspects of enzyme behavior and are, therefore, not expected to align on an absolute scale. pIC_50_ reflects inhibitory potency, whereas intrinsic clearance captures overall metabolic turnover, which includes contributions beyond binding. Nonetheless, comparing these endpoints remains informative because both are shaped by how substrates interact with the variant-specific CYP3A4 active site, allowing us to assess whether the model captures consistent relative trends across mutations and compounds. We assessed how well the pipeline predicted the effect of genetic variations on drug metabolism using WT CYP3A4 and clinically relevant variants (A370S, F113I, I118V, I427V, L373F, M445T, and R130Q)^86^ across six test substrates representing distinct metabolic pathways.

The Boltz-2 predictions demonstrated impressive performance for CYP3A4 (Figure 3A iii; Figure S6). All experimental data^86^^,^^87^^,^^88^^,^^89^ used for comparison are shown in Table S3. For WT CYP3A4, correlations with experimental clearance data were strong (r = −0.83 to −0.86, R^2^ = 0.69–0.74), with the negative correlation reflecting the expected inverse relationship between predicted binding affinity and metabolic clearance rates. Pairwise ranking accuracy for WT was also consistently high across all settings (86.7%; Figure 3A iii, second row). In other words, compounds predicted to bind more tightly to the enzyme tend to be metabolized and eliminated more slowly, as stronger binding reduces the amount of free drug available for metabolism, thereby slowing its breakdown and clearance.

For CYP3A4 variants, we report a within-drug correlation to avoid confounding by substrate-level offsets. Specifically, for each drug (quinine and sildenafil), we mean-center both experimental endpoint values and predicted scores within that drug, then pool the centered points across drugs and compute Pearson r. This metric reflects how well the model ranks mutants within each drug, rather than between-drug differences. Under this analysis, within-drug correlation is modest in settings 0 and 1 (r = 0.30 and 0.19) but improves substantially in setting 2 (r = 0.66, R^2^ = 0.43, *p* = 0.0197), suggesting that increased affinity sampling better resolves mutation-dependent effects. Consistent with this, pairwise ranking accuracy remains high overall and shows a clear gain for mutants in setting 2 (∼80%–85%) relative to settings 0 and 1 (∼55%–70%) (Figure 3A iii, second row). The notable improvement in accuracy with increased affinity sampling (setting 2) likely reflects a better resolution of subtle, mutation-specific effects that are otherwise obscured in this particularly complex system. CYP3A4 poses additional challenges compared to other targets, including the presence of the heme cofactor, which plays a central role in catalysis by enabling oxidative metabolism and directly influencing ligand binding modes within the active site. Additionally, the indirect relationship between pIC_50_ and intrinsic clearance introduces further complexity, as inhibitory potency does not directly reflect metabolic turnover. Together, these factors introduce potential confounding effects, making the weaker performance because of fewer sampling steps (i.e., settings 0 and 1) not unexpected. Encouragingly, the substantial gains in both correlation and ranking in setting 2 suggest that, with sufficient sampling, the model can overcome these challenges and capture meaningful variant-dependent trends despite the mechanistic differences between predicted and experimental endpoints.

Drug-specific analysis revealed consistent prediction accuracy across different CYP3A4 substrates and metabolic pathways (Figure S7). The model showed remarkable consistency across all sampling settings, with minimal variation in prediction errors for most drug-variant combinations. Sildenafil was predicted to have the largest deviation from experimental values across all variants, potentially reflecting specific limitations in modeling this compound and its unique metabolic pathway. The other substrates, including estradiol, rifampicin, and quinidine, showed excellent agreement with experimental clearance data across all genetic variants. The performance suggests promise for this approach in pharmacogenomic applications involving drug metabolism, where accurate prediction of variant effects on clearance is critical for personalized dosing strategies.

### Receptor tyrosine-protein kinase erbB-2 (HER2)

The Human Epidermal Growth Factor Receptor 2 (HER2), also known as ERBB2, is a member of the ERBB family of receptor tyrosine kinases and plays a central role in regulating cell growth, survival, and differentiation through activation of downstream signaling pathways such as PI3K/AKT and MAPK.^90^^,^^91^ HER2 is a critical oncogenic driver in a subset of breast, gastric, and other cancers, where gene amplification or overexpression leads to uncontrolled cell proliferation and poor clinical prognosis.^34^ Structurally, HER2 consists of an extracellular ligand-binding domain that mediates receptor dimerization, a single-pass transmembrane helix, and an intracellular tyrosine kinase domain responsible for propagating growth signals. The kinase domain is the primary target of several FDA-approved small molecule therapeutics, including lapatinib, neratinib, and tucatinib, which inhibit its catalytic activity.^34^^,^^92^^,^^93^^,^^94^ However, mutations within this domain frequently confer acquired resistance to targeted therapies, posing a major challenge for precision oncology.^35^^,^^95^^,^^96^ For this analysis, we focused on modeling the intracellular kinase domain rather than the full transmembrane receptor, evaluating our pipeline against experimental data for WT HER2 and two clinically relevant variants (L755S and V842I) across a panel of 12 kinase inhibitors.

Despite modeling only the isolated kinase domain rather than the complete receptor structure, the Boltz-2 approach demonstrated excellent predictive performance across all evaluated conditions (Figure 3A iv; Figure S8). All experimental data^95^^,^^96^^,^^97^^,^^98^^,^^99^^,^^100^^,^^101^^,^^102^^,^^103^^,^^104^ used for comparison are recorded in Table S4. WT HER2 kinase domain showed the strongest correlation with experimental data, achieving r = 0.82–0.84 (R^2^ = 0.66–0.70) across the three sampling settings, representing the highest correlation observed among all protein targets evaluated. The strong performance suggests that the kinase domain contains the primary determinants of drug binding affinity, validating the domain-focused modeling approach for this target class. Mutant variants showed moderate but still meaningful correlations (r = 0.53–0.54, R^2^ = 0.28–0.29, *p* ≈ 0.03) with RMSE values of 0.79–0.82. Although accuracy was reduced compared with WT, the statistically significant correlations indicate that the model captures variant-specific effects despite the limited availability of experimental data. Pairwise ranking accuracy remained above random for both mutant and WT HER2 panels (Figure 3A iv), although WT ranking was somewhat lower than the corresponding correlation values might suggest.

Analysis of drug-specific prediction patterns revealed distinct performance profiles across different kinase inhibitor classes (Figure S9). First-generation inhibitors such as lapatinib and gefitinib showed consistent predictions across variants and sampling conditions, while the covalent inhibitor afatinib demonstrated underestimation of binding affinity across all variants, potentially reflecting limitations in modeling covalent bond formation within the current pipeline. Newer agents like neratinib and tucatinib showed variable performance in mutant backgrounds, with poziotinib and tyrphostin-ag-879 exhibiting the largest deviations from experimental values. Notably, the model maintained strong consistency across sampling parameters for most compounds, with minimal variation between different sampling settings. The strong predictive performance despite modeling only the kinase domain highlights the potential for domain-specific approaches in large, multi-domain proteins where full-length modeling may be computationally prohibitive or structurally complex.

### Structural validation against experimental data

The structural validation against experimental crystal structures and cryo-EM data provides crucial insights into the predictive accuracy of the pipeline to reproduce authentic drug binding modes beyond simple affinity predictions. We compared predicted structures with experimental complexes: hERG bound to astemizole (PDB: 8ZYO)^105^ and E4031 (PDB: 8ZYP),^105^ Na_V_1.5 with quinidine (PDB: 6LQA),^73^ and HER2 kinase domain with JBJ-08-178-01 (PDB: 7JXH).^95^ Notably, Boltz-2 was trained on PDB data available through June 2023.^28^ While Na_V_1.5 and HER2 structures were available for training as they were released on March and September 2021,^73^^,^^95^ respectively, the two hERG structures were released in September 2024,^105^ post-dating the cutoff by over a year. Accordingly, the hERG structures provide a blind structural evaluation, whereas the Na_V_1.5 and HER2 structures predate the cutoff and are interpreted more cautiously as recovery checks rather than strict hold-out tests.

**Figure 4:**
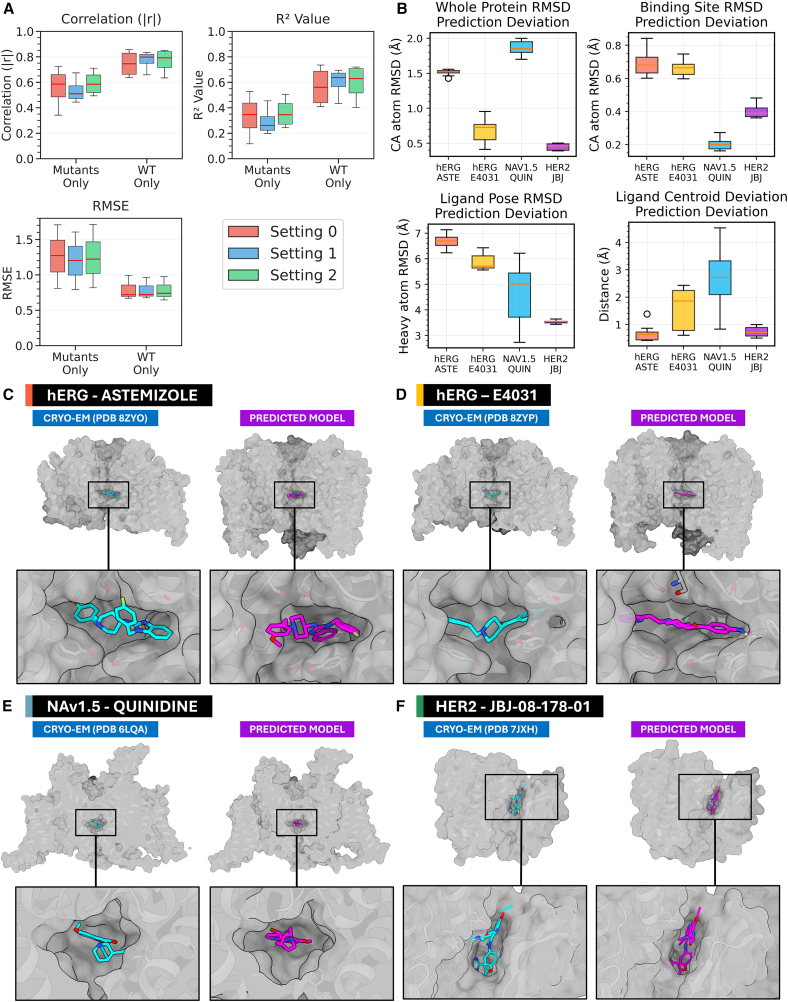
Performance analysis and structural comparison of Boltz-2 predictions with experimental structures (A) Performance metrics across sampling settings showing absolute correlation coefficients (|r|), R^2^ values, and RMSE (lower value indicates less error) between Boltz-2-predicted drug potency (pIC_50_) and experimental pIC_50_ values (or enzyme intrinsic clearance for CYP3A4) for all data combined, mutants only, and WT only. Performance is summarized across setting 0, the Boltz-2 default using 200 sampling steps and 5 diffusion samples; setting 1, using 300 sampling steps and 7 diffusion samples; and setting 2, using 400 sampling steps and 9 diffusion samples. Box-and-whisker plots summarize the spread of performance values within each comparison group and sampling setting. (B) Structural prediction accuracy metrics comparing whole protein root-mean-square deviation (RMSD), binding site RMSD, ligand pose RMSD, and ligand centroid deviation between Boltz-2 predictions and experimental crystal structures for representative protein-drug complexes. For each structural metric, box-and-whisker plots show the distribution of prediction deviations for each evaluated complex. (C–F) Side-by-side structural comparisons of experimental structures (left) and Boltz-2 predictions (right) for: (C) hERG with astemizole,^105^ (D) hERG with E4031,^105^ (E) Na_V_1.5 with quinidine,^73^ and (F) HER2 with JBJ-08-178-01.^95^ Surface representations show the overall protein structure with binding pockets highlighted, while detailed views below show ligand binding poses in stick representation (cyan for experimental and magenta for predicted structures). Structural alignments demonstrate the pipeline’s ability to reproduce experimentally observed binding modes across diverse protein-drug combinations.

Performance analysis across Boltz-2 sampling settings showed broadly consistent prediction quality across the three configurations (Figure 4A). Increasing sampling generally produced modest improvements in correlation, with the highest median values often observed for setting 2 (400 sampling steps, 9 diffusion samples). However, the differences between settings 1 and 2 were small, and RMSE values were comparable across the two higher sampling regimes. This pattern indicates diminishing returns at the highest sampling level: additional refinement can slightly improve stability of affinity estimates but provides only marginal gains relative to the intermediate setting. Consequently, setting 1 offers a practical balance between computational cost and predictive performance, while setting 2 represents the upper bound of sampling used in this study.

The structural accuracy assessment demonstrated varying degrees of success in reproducing experimental binding poses across different targets (Figure 4B). Whole protein root-mean-square deviation (RMSD) of predicted models from experimental structures ranged from 0.5 to 2.0 Å, indicating generally accurate overall protein structural prediction. The HER2 kinase domain showed the lowest deviations, whereas Na_V_1.5 displayed the highest structural variability. Binding site RMSD measurements, focusing specifically on residues within 5 Å of bound ligands, showed more target-specific patterns, with hERG and HER2 demonstrating predicted sub-angstrom accuracy while Na_V_1.5 exhibited greater binding site prediction challenges.

Ligand pose prediction (i.e., the predicted 3D binding orientation of a drug within its target) accuracy varied considerably across protein-drug combinations, with some achieving high fidelity to experimental structures while others showed deviations. For hERG complexes with astemizole and E4031, the predicted binding poses closely recapitulated the experimental conformations,^105^ capturing key interactions within the channel pore (Figures 4C and 4D). The astemizole complex showed particularly strong agreement, with the predicted pose overlaying almost perfectly with the cryo-EM structure,^105^ while E4031 binding reproduced the characteristic deep insertion into the channel cavity observed experimentally.^105^ The high fidelity observed in the hERG-astemizole and E4031 complexes is particularly noteworthy, given their status as blind predictions, suggesting that the pipeline’s learned physical and chemical priors may be robust enough to generalize to novel, post-training structural data.

Na_V_1.5-quinidine interactions presented greater prediction challenges, with slightly larger ligand centroid deviations (reflecting shifts in predicted ligand binding location relative to the experimental structure) and pose RMSD values (reflecting overall 3D ligand pose accuracy) compared with other complexes (Figure 4E). The predicted quinidine binding mode, while positioned within the generally expected region of the channel pore, showed a different orientation compared to the experimental structure.^73^ This deviation suggests that even with training exposure, the inherent complexity and conformational flexibility of the sodium channel pore remain a significant challenge for current predictive models.

HER2 kinase domain predictions with JBJ-08-178-01 demonstrated intermediate accuracy, successfully positioning the inhibitor within the ATP-binding pocket but with some differences in specific molecular contacts (Figure 4F). The molecular interaction analysis revealed reasonably good reproduction of π-interactions and salt bridges characteristic of kinase inhibitor binding, though some hydrogen bonding patterns differed from experimental observations.^95^ The predicted pose captured the general binding mode characteristic of kinase inhibitors, while subtle differences in side chain interactions highlight areas for methodological refinement.

Detailed molecular interaction analysis revealed the extent to which predicted structures capture the specific binding mechanisms observed experimentally (Figure S10). The hERG-astemizole complex demonstrated excellent reproduction of hydrogen bonding patterns and hydrophobic contacts, with high overlap in interacting residues and balanced interaction count ratios near unity. In contrast, the hERG-E4031 complex showed some discrepancies in hydrogen bonding and hydrophobic contact patterns, though the overall binding mode remained consistent. Na_V_1.5-quinidine interactions showed the greatest deviation in molecular contacts relative to other cases. However, the physical distance deviation remained small, with altered hydrogen bonding patterns and reduced overlap in interacting residues, consistent with the larger structural deviations observed in pose prediction.

The structural comparisons of the model predictions to experimentally available data reveal that while the pipeline shows promise for reproducing experimental binding modes, performance remains target- and compound-specific. The strong correlations with experimental affinity data do not always translate directly to expected structural accuracy, highlighting the complex relationship between binding pose prediction and affinity estimation. The molecular interaction analysis particularly emphasizes that even when overall binding poses appear similar, detailed contact patterns may vary significantly, which could impact the accuracy of mutation effect predictions that depend on specific residue-drug interactions.

### Performance comparison against other computational approaches

To contextualize Boltz-2 performance, we benchmarked it against five comparator methods representing two computational paradigms: structure-based docking (GALigandDock,^16^ RosettaLigand with receptor backbone/side-chain flexibility,^106^ and AutoDock Vina^18^) and deep neural networks trained to predict binding affinity directly from drug chemical features and target amino acid sequences, without generating 3D structures or docking poses (Deep Target Affinity, or DTA, models: DeepDTA^23^ and GraphDTA^22^) (Figure 3B; Figures S11–S15). All methods were evaluated on identical protein-mutation-drug entries across hERG, Na_V_1.5, CYP3A4, and HER2 by using identical experimental drug datasets.

As summarized in Figure 3B, Boltz-2 consistently showed the strongest overall agreement with experimental data across both mutant and WT panels. In mutants, mean Pearson correlations for Boltz-2 ranged from approximately 0.48 to 0.60 across settings, exceeding those of RosettaLigand (∼0.40), GALigandDock (∼0.37), AutoDock Vina (∼0.36), DeepDTA (∼0.34), and GraphDTA (∼0.23). In WT proteins, Boltz-2 again led with mean correlations of approximately 0.74–0.76, whereas structure-based docking methods formed an intermediate group (∼0.45–0.50) and sequence-based DTA approaches remained substantially lower (∼0.17–0.33). Differences among Boltz-2 sampling settings were modest compared with the cross-method gaps, indicating that the performance advantage primarily reflects the modeling framework rather than simply increased sampling.

Among comparator methods, RosettaLigand showed the strongest overall performance, followed closely by GALigandDock and AutoDock Vina, with all structure-based approaches consistently outperforming the sequence-based models. GALigandDock uses a genetic algorithm to explore large pose ensembles and refines ligand placement using Rosetta scoring while allowing local side-chain flexibility near the binding site, which can be effective when the pocket is reasonably well defined. RosettaLigand (with flexible receptor protocol implemented) further samples receptor degrees of freedom, including side-chain repacking and limited backbone motion near the ligand. Its consistently strong performance across both WT and mutant panels suggests that incorporating receptor flexibility can be beneficial, although gains are still constrained by scoring accuracy and pose selection. AutoDock Vina, despite using a simpler semi-empirical scoring function and more limited receptor flexibility, achieved comparable performance across most targets, indicating that increased flexibility alone does not guarantee improved accuracy. In practice, performance remains strongly influenced by scoring calibration, and in state-dependent systems such as ion channels, binding can depend on broader conformational changes that are difficult to capture with local flexibility and a single-pocket docking setup.

The learned models, DeepDTA and GraphDTA, represent a fundamentally different strategy. These models are trained on large drug-target interaction datasets and learn statistical associations between molecular features and binding affinity without explicitly modeling 3D structure. While efficient and scalable, their predictions depend heavily on training distribution and sequence similarity to known targets. In this benchmark, which includes membrane proteins, enzyme variants, and oncogenic mutations, the DTA models showed greater variability and generally lower agreement with experimental data. This likely reflects limited mutation-specific structural context and the absence of explicit modeling of variant-induced conformational changes.

In contrast, Boltz-2 integrates structural prediction and affinity estimation within a unified deep learning framework. Rather than docking a ligand into a fixed receptor, it jointly predicts protein conformation, ligand pose, and binding score. This allows pocket geometry to adapt to the ligand during inference, implicitly modeling induced-fit effects. The improved performance across diverse targets suggests that this integrated approach better captures mutation-induced structural perturbations than either fixed-pocket docking or sequence-only affinity prediction.

Overall, the comparison indicates that structure-aware modeling remains important for mutation-sensitive prediction tasks, and that the ability to adapt binding pocket geometry during prediction confers a measurable advantage. While advanced physics-based simulations could, in principle, further refine predictions, their computational cost limits scalability. Within a unified and tractable framework, Boltz-2 provides the most consistent cross-target performance across both WT and mutant panels.

## Discussion

This study demonstrates the potential of deep learning-based biomolecular modeling to rapidly evaluate how amino acid mutations affect drug binding across diverse therapeutic protein targets. By integrating Boltz-2, a deep learning model for biomolecular structure and binding affinity prediction, into an accessible workflow, BoltzOmics enables systematic assessment of mutation-dependent drug binding changes with substantially reduced setup and computational burden compared with traditional structure-based approaches. Across hERG, Na_V_1.5, CYP3A4, and HER2, predicted values generally showed meaningful agreement with experimental measurements, with differences largely reflecting the inherent structural complexity of each protein class and the amount of experimental data available for validation.

Figure 3A clarifies these cross-target patterns. Across all four proteins, Pearson correlations and pairwise ranking accuracies were generally strongest for WT CYP3A4 and HER2, while hERG showed consistently moderate-to-strong performance across settings. Na_V_1.5 displayed the greatest sensitivity to sampling configuration, particularly in the mutant panel, where the lowest setting produced the strongest correlation. Pairwise ranking accuracy remained above the 50% random baseline in all panels and was often in the ∼60%–80% range, indicating that the model generally preserved the relative ordering of stronger and weaker interactions even when absolute agreement varied by target. This distinction is important because, for screening applications, identifying which variants or compounds are more likely to show altered binding can be more informative than recovering exact potency values.

In several target-specific analyses, mutant models displayed higher correlations between pIC_50_ and experimental binding affinity data (or intrinsic clearance for CYP3A4) than WT models, indicating the potential to effectively rank drug affinities across variants. However, in some cases, higher correlation values were accompanied by larger RMSEs, meaning that while the model captures relative trends well, the absolute prediction values may deviate more from experimental measurements. In practical terms, this indicates that the pipeline is more effective at ranking variants more likely to influence drug sensitivity, even if exact potency estimates may need further refinement. For pharmacogenomic applications, rapid and reliable ranking can help prioritize experimental evaluation of the most impactful mutation-drug combinations.

Prediction patterns indicate target-specific tendencies, such as overestimation for certain hERG and Na_V_1.5 protein-drug pairs. These trends likely reflect both the underlying structural assumptions in the models and variation in the experimental reference data, which were collected using different assay formats or cell systems. While variability is an inherent challenge in benchmarking computational predictions, the model predictions indicate consistent relative rankings across variants. This result indicates robustness to experimental methodological differences, a key need for early-stage screening.

Prediction accuracy was influenced more by the structural and functional dynamics of proteins than by simple molecular descriptors such as drug size. This is supported by Figures S3, S5, S7, and S9, which show drug-specific prediction errors for hERG, Na_V_1.5, CYP3A4, and HER2 variants, respectively, across different sampling conditions, with drugs ordered from highest to lowest molecular weight. For ion channels, experimental IC_50_ can depend on gating state and conformational dynamics, which are not fully captured in Boltz-2. Accordingly, hERG and Na_V_1.5 inhibition is strongly state dependent, whereas Boltz-2 predicts a single representative protein-ligand conformation. Experimental IC_50_ values may, therefore, reflect both binding preference for particular channel states and the fraction of time the channel occupies those states. Mutations can reduce apparent drug potency by shifting gating equilibria or kinetics without directly altering binding-pocket geometry, a mechanism that cannot be captured by a single static predicted complex. We therefore interpret predicted affinities primarily as relative binding propensity and ranking signals, especially when mutation effects are likely to be local and structural. Capturing state occupancy and allosteric gating effects will require complementary approaches such as electrophysiological modeling, kinetic schemes, or multi-state molecular dynamics simulations. In future work, incorporating MSA subsampling and state-guided structural templates may provide a practical route to explore alternative conformational states more explicitly. In contrast, for more rigid proteins, such as the HER2 tyrosine kinase, high accuracy in model predictions indicates that domain-focused modeling can provide a computationally efficient approach while still yielding meaningful insight into the effects of gene variants. These findings underscore the importance of follow-up studies that directly compare flexible and rigid binding environments, allow for further model optimization, and interrogate effects of intrinsically disordered regions, to better understand how conformational heterogeneity shapes prediction accuracy.

Detailed molecular interaction analysis provided additional insights into the structural basis of prediction accuracy differences across targets (Figure S10). The reproduction of specific binding mechanisms varied considerably between protein-drug combinations. The hERG complexes generally showed better preservation of interaction patterns, with high overlap in interacting residues and balanced interaction count ratios. In contrast, Na_V_1.5 predictions exhibited more deviation in molecular contact patterns, consistent with long-standing structural prediction challenges for Na_V_1.5. The HER2 kinase domain showed intermediate performance, successfully capturing characteristic kinase inhibitor interactions while showing some variability in detailed contact profiles. These findings highlight that accurate affinity prediction does not necessarily imply atomic-level recovery of a single experimental binding pose, particularly for flexible targets such as ion channels. Boltz-2 is trained to predict binding strength rather than to recover a unique ligand orientation, and experimental IC_50_ values for ion channels measure functional inhibition rather than a specific static binding configuration. As a result, multiple ligand orientations or transient interaction patterns may produce similar levels of channel block even if detailed hydrogen-bond or salt-bridge networks differ from those observed in a particular cryo-EM structure. In such cases, predicted affinity reflects overall physicochemical compatibility between the ligand and the binding environment rather than exact atomic contacts. Predicted affinities should, therefore, be interpreted primarily as indicators of functional binding strength, while predicted poses for highly flexible ion channels should be viewed cautiously and not over-interpreted at the level of individual molecular interactions. In addition, diffusion-based co-folding does not strictly enforce chirality during sampling, and rare stereochemical inversions may occur.

Boltz-2 contains separate structural and affinity modules trained on different data sources. The structural module is trained primarily on experimentally determined macromolecular structures from the Protein Data Bank (PDB), whereas the affinity module is trained on large public drug-target interaction resources such as ChEMBL,^107^ BindingDB,^108^ and PubChem.^109^ These public affinity databases are dominated by WT measurements, while mutation-specific drug response data are more often scattered across individual studies rather than deposited as unified benchmark datasets. Accordingly, the mutation-resolved drug panels used here are unlikely to exist as an intact set in the Boltz-2 affinity training corpus. However, because the exact affinity-training snapshots are not released publicly, we cannot completely exclude the possibility that some individual WT measurements, closely related ligands, or homologous targets were present in the training data.

While interpretation of all model results should consider available data and the potential for validation, the pipeline consistently revealed meaningful mutation-drug interaction patterns across all targets studied. The scarcity of experimental datasets, such as the small number of CYP3A4 substrates tested *in vitro* and the relatively narrow mutant panels examined for Na_V_1.5 and HER2, reflects the enormous experimental effort required to generate high-quality pharmacogenomic measurements. As a result, current literature captures only a fraction of the possible mutation space. Our evaluation therefore focused on mutations within known drug binding sites, even though structural changes from distant mutations, allosteric effects, or altered protein stability can also influence drug binding. By filling in some of these gaps computationally, the results and the tool presented here can help prioritize which variants and drug combinations merit deeper experimental investigation, hopefully kickstarting broader follow-up studies that expand the diversity of available genetic variants. Furthermore, variability in experimental reference datasets adds further noise, and for ion channels, the inability to account for state-dependent binding remains a broader modeling challenge. These limitations highlight the value of complementary strategies that can help narrow the search space and predict drug binding to alternative protein conformations including previously described state-guided AlphaFold approaches for hERG^110^ In parallel, recent deep learning approaches for DTA prediction have improved representation learning for proteins and ligands, including models incorporating multiscale attention mechanisms and geometric message passing to better capture interaction patterns,^111^^,^^112^ and may provide useful complementary directions for future benchmarking.

Despite these considerations, the combination of accuracy, flexibility, and accessibility makes this approach a powerful tool for rapid hypothesis generation and variant prioritization. Although precision oncology has grown into a global industry exceeding $100 billion each year, therapeutic resistance remains the principal obstacle to lasting success. Improving our capacity to differentiate responders from non-responders based on genetic and molecular context, whether arising from germline variants or somatic mutations, will be essential to enhance clinical outcomes while minimizing costly and ineffective treatments. The ability to rank relative drug sensitivities enables the identification of high-impact variants for deeper experimental investigation, even when absolute potency values require validation. By providing an open-source and interactive platform, we enable researchers across disciplines to integrate variant-aware drug screening into their workflows, using it as an efficient first-pass filter to guide and accelerate precision medicine research.

This work establishes deep learning-based structure prediction as a transformative tool for computational pharmacogenomics, fundamentally improving the speed-accuracy paradigm through unprecedented computational efficiency that enables previously intractable precision medicine investigations. The significance of results in 3–5 min versus hours or days cannot be overstated: With over 1 billion genetic variants identified in human populations^113^ and thousands of marketed drugs, the combinatorial space of mutation-drug interactions represents billions of potential relationships requiring assessment. Over the past three decades, the rate of genetic variant discovery has far outpaced functional characterization of variant effects. This is because traditional physics-based methods cannot scale to address this vast prediction space within research, industrial, or clinically relevant time frames.

The rapid pharmacogenomic screening demonstrated in our study enables transformative applications that were not previously feasible. Population-level pharmacogenomic screening can be applied to identify potential drug-variant interactions before clinical encounters. Real-time clinical decision support based on genotype may become feasible, where genetic test results could be rapidly cross-referenced against prescribed medications. Drug development can incorporate large-scale variant effect prediction during early discovery phases, potentially identifying safety liabilities or resistance mechanisms before expensive clinical trials. Beyond reducing cost and time, the improvements in both speed and predictive accuracy might make it possible to evaluate hundreds to thousands of variants across multiple drug classes in days rather than months. In preclinical research, the potential for high throughput pharmacogenomic screening can accelerate hypothesis generation, allowing for prioritization of variants for experimental validation, and more efficient allocation of laboratory resources. In the pharmaceutical industry, rapid *in silico* evaluation of variant-drug interactions can inform lead optimization, early safety assessment, and adaptive trial design, reducing late-stage attrition. In clinical settings, the same framework offers the potential for prescreening of patient genotypes against entire formularies, flagging high-risk variant-drug interactions in advance and supporting precision prescribing at the point of care. Together, these advances establish a bridge from computational prediction to real-world translational impact, positioning AI-driven variant screening as a foundational tool across the biomedical pipeline.

Most critically, this approach transforms the prediction of genetic variant effects on protein structure and drug interaction from a computational bottleneck requiring extensive resources into a rapid screening tool that informs experimental prioritization and clinical decision-making. While experimental validation is still required for definitive application, the ability to rapidly generate hypotheses and identify high-priority variants represents a fundamental shift in pharmacogenomic research approaches.

The broader implications extend to system-level precision medicine. The speed of deep-learning-based prediction makes it possible to perform large-scale analyses that can uncover hidden patterns in mutation-drug interactions, reveal shared mechanisms across drug classes, and guide the development of more sophisticated predictive models. As training datasets grow and algorithms continue to improve, the balance of speed and accuracy offered by this approach positions it as a key enabler for implementing precision medicine at the population scale.

The trade-off between accuracy and speed strongly favors rapid screening approaches in the current precision medicine era, where the primary challenge is not achieving perfect predictions but enabling assessment of genetic variation affecting drug response. Even imperfect predictions provide valuable clinical utility when they can guide experimental resources toward the most relevant mutation-drug interactions while enabling broad variant landscape assessment.

This work provides a crucial foundation for making computational mutation effect prediction practical for widespread clinical and research applications. The goal is not to replace experimental validation but rather to allow for intelligent guidance, ensuring limited resources target the most clinically relevant interactions while enabling rapid assessment of the broader variant landscape that defines individual drug response profiles. The potential broad application of rapid AI-based pharmacogenomics represents a key step toward making personalized drug therapy a clinical reality.

### Limitations of the study

Several limitations should be considered when interpreting the results of this study. First, the benchmark datasets remain limited in size and scope. Although the four selected targets represent diverse and therapeutically important protein classes, the number of experimentally characterized mutation-drug combinations remains small relative to the enormous possible variant-drug interaction space. As a result, the reported correlations should be interpreted as evidence of promising predictive behavior rather than definitive estimates of general performance across all variants, compounds, or target families.

Second, the analysis focused primarily on mutations located within or near known drug-binding regions. This should be viewed as a deliberate benchmarking constraint rather than a complete representation of all mechanisms that influence drug response. Variants outside the binding pocket can also alter drug sensitivity through long-range allosteric effects, changes in protein stability, altered expression, modified trafficking, or shifts in conformational equilibria. Because these mechanisms were not systematically tested here, future studies should expand the benchmark to include distal and allosteric variants.

Third, experimental reference data were compiled from published studies that used different assay formats, cell systems, experimental conditions, and endpoint definitions. Rather than representing a new observation separate from the discussion, this heterogeneity defines an important source of benchmark uncertainty. It may contribute to cases where the model captures relative trends but shows larger deviations in absolute predicted values. Therefore, BoltzOmics predictions should be treated primarily as ranking and prioritization signals rather than definitive absolute affinity measurements.

Fourth, Boltz-2 predicts representative protein-ligand complexes and binding-related scores, but it does not fully model dynamic biological processes. As discussed earlier for hERG and Na_V_1.5, this is especially relevant for ion channels, where apparent IC_50_ values can reflect channel state, gating kinetics, and state occupancy rather than binding-pocket geometry alone. Capturing these effects will likely require complementary methods such as electrophysiological modeling, kinetic state models, multi-state structural prediction, and/or molecular dynamics simulations.

Fifth, predicted ligand poses should not always be interpreted as unique atomic-level binding mechanisms. This point is distinct from affinity prediction: a model may recover useful ranking information even when the detailed ligand orientation or contact network differs from a reference structure. This was most apparent for Na_V_1.5, consistent with the structural complexity and flexibility of sodium channels. Therefore, predicted interactions such as hydrogen bonds, salt bridges, and hydrophobic contacts should be interpreted cautiously, particularly for flexible or state-dependent targets. In addition, diffusion-based co-folding may rarely produce stereochemical artifacts, including chirality inversions, which should be checked during structural interpretation.

Sixth, the training data composition of Boltz-2 introduces uncertainty when evaluating benchmark independence. As noted earlier, Boltz-2 uses separate structural and affinity modules trained on large public structural and drug-target interaction resources. Because the exact affinity-training snapshots are not publicly released, we cannot fully determine whether some individual WT measurements, closely related ligands, or homologous targets were represented during training. However, mutation-specific drug response datasets are typically scattered across individual studies, making it unlikely that the mutation-resolved drug panels evaluated here were present as intact benchmark sets in the affinity training corpus.

Finally, this study evaluated retrospective agreement with published experimental data rather than prospective experimental validation of newly predicted mutation-drug interactions. Prospective validation will be essential to determine how well BoltzOmics performs when applied to novel variants, new compounds, or targets with limited prior characterization. Future work should include blinded experimental testing of high-priority predictions generated by the pipeline, ideally across both rigid and flexible protein systems, to better define where the approach is most reliable.

Collectively, these limitations define the current scope of BoltzOmics as a rapid prioritization framework rather than a replacement for experimental validation or more mechanistic simulation. Future benchmarking should combine prospective experimental testing, broader variant panels, multi-state structural approaches, and complementary DTA models to better define where the method is most reliable. Despite these limitations, BoltzOmics provides a scalable and accessible framework for prioritizing mutation-drug interactions. Its greatest current value is as a rapid hypothesis-generation and ranking tool that can narrow the search space, guide experimental design, and support more systematic exploration of variant-aware drug discovery.

## Resource availability

### Lead contact

Further information and any reasonable requests should be directed to and will be fulfilled by the lead contact, Hajar Amini (hamini@health.ucdavis.edu).

### Materials availability

This study did not generate new unique reagents.

### Data and code availability

• Original simulation data and input files have been deposited at Zenodo and are publicly available as of the date of publication at https://doi.org/10.5281/zenodo.19244181.
• All original code has been deposited on GitHub and is publicly available at the date of publication at https://doi.org/10.5281/zenodo.20388203. URLs are listed in the key resources table.
• Any additional information required to reproduce the analyses reported in this paper is available from the lead contact upon request.

## Acknowledgments

We would like to thank all members of the UC Davis Center for Precision Medicine and Data Sciences and K.N.’s cats, Momo and Orange, for helpful discussions and support. This work was supported by; 10.13039/100000050National Heart, Lung, and Blood Institute (NHLBI) grants R01HL128537, R01HL174001, R01HL085844, R01HL152681, and U01HL126273 (to C.E.C.); UC Davis Department of Physiology and Membrane Biology Research Partnership Fund (to C.E.C.); as well as UC Davis T32 Predoctoral Training in Basic and Translational Cardiovascular Medicine fellowship supported in part by 10.13039/100000050NHLBI Institutional Training Grant T32HL086350 (to K.N.). Computer allocations were provided through Advanced Cyberinfrastructure Coordination Ecosystem: Services & Support (ACCESS) grant BIO250260 (to K.N.).

## Author contributions

K.N., conceptualization, methodology, software, validation, formal analysis, investigation, data curation, visualization, writing – original draft, and writing – review and editing; K.L.C., supervision, project administration, and writing – review and editing; C.E.C., conceptualization, supervision, funding acquisition, project administration, and writing – review and editing; H.A., conceptualization, methodology, validation, formal analysis, investigation, data curation, supervision, project administration, writing – original draft, and writing – review and editing.

## Declaration of interests

The authors declare no competing interests.

## Declaration of generative AI and AI-assisted technologies in the writing process

The authors used ChatGPT to assist with proofreading for grammar issues and code debugging. The authors subsequently reviewed and edited the text as needed and take full responsibility for the content of the publication.

## STAR★Methods

### Key resources table

**Table:** 

REAGENT or RESOURCE	SOURCE	IDENTIFIER
**Deposited data**		

Title: “BoltzOmics: Rapid Assessment of Genetic Variant Effects on Drug Binding Using Boltz-2 Deep Learning Model”	Zenodo	https://zenodo.org/records/19244182 https://doi.org/10.5281/zenodo.19244181

**Software and algorithms**		

BoltzOmics (for result reproduction)	Zenodo	https://doi.org/10.5281/zenodo.20388203
BoltzOmics (latest version)	Github	https://github.com/k-ngo/boltzomics/
Boltz-2	Github	https://github.com/jwohlwend/boltz
Streamlit	Github	https://github.com/streamlit/streamlit
UniProt Knowledgebase	UniProt	https://rest.uniprot.org
EBI NCBI BLAST service	EMBL-EBI	https://blast.ncbi.nlm.nih.gov/Blast.cgi
EBI Proteins API	EMBL-EBI	https://www.ebi.ac.uk/proteins/api
AlphaMissense	Github	https://github.com/google-deepmind/alphamissense
Rosetta Software Suite	Rosetta	https://www.rosettacommons.org
AutoDock Vina	Github	https://github.com/ccsb-scripps/AutoDock-Vina
DeepDTA	Github	https://github.com/hkmztrk/DeepDTA
GraphDTA	Github	https://github.com/thinng/GraphDTA

### Method details

#### Interactive computational pipeline for pharmacogenomic screening

We developed a comprehensive computational pipeline delivered as accessible, fully interactive, graphical, no-code software that runs with minimal setup. It operates in two primary modes: a drug–mutation screening mode for predicting variant effects on a single protein, and a protein–drug screening mode for large-scale pharmacological profiling across multiple protein targets and compounds. A structure-only mode is also available for protein folding and structural studies without ligands, and the software produces interactive plots and visualizations that are directly suitable for publication. The complete codebase is provided as an open-source program in our GitHub repository: https://github.com/k-ngo/boltzomics.

#### Protein, ligand, and co-factor specification

Protein targets were specified using their amino acid sequences in single-letter code. The pipeline accepts single WT sequences via manual text entry or multiple sequences in FASTA format for batch processing. Multi-chain proteins are supported using a colon-separated format (e.g., *SEQUENCE1*:*SEQUENCE2*). Drug compounds were provided as Simplified Molecular Input Line Entry System (SMILES) strings, either through direct text input for single or multiple semicolon-separated entries or via a FASTA-formatted file for larger libraries. The pipeline also supports the integration of essential biological co-factors (e.g., ATP, NAD, heme), which can be specified by their SMILES string or Chemical Component Dictionary (CCD) code. Advanced modeling features are also supported, including the use of an existing structural file (.cif) as a template to guide prediction and the specification of binding pocket constraints to steer ligand docking toward specific residues.

#### Mutation discovery and generation

To evaluate the impact of amino acid substitutions, the pipeline integrates two workflows for defining variants: automated discovery and manual specification. The automated discovery process begins with a user-provided WT protein sequence. The system queries the UniProt database^114^ and employs the EBI Basic Local Alignment Search Tool (BLAST) service to identify the canonical protein match. Subsequently, it retrieves a comprehensive list of known variants from the EBI Proteins API.^115^ Where available, this list is enriched with pre-calculated functional prediction scores from SIFT (Sorting Intolerant From Tolerant)^38^ and PolyPhen-2 (Polymorphism Phenotyping v2),^39^ which help classify variants as potentially “deleterious” or “benign.” Variant annotation is extended by incorporating AlphaMissense predictions,^40^ together with clinical significance labels from public variant resources.^116^ These annotations are combined into a practical prioritization layer so that users can rank variants before screening rather than treating all mutations as equally important.

If the initial input was a sequence fragment, the system intelligently maps the full-length protein’s mutation positions onto the user’s partial sequence. In addition, users can manually define mutations using a simple text-based format. The system supports comma-separated lists for individual variants (e.g., Y652A, F656A) or hyphen-separated lists to generate a single variant with multiple substitutions (e.g., Y652A-F656A). For each variant identified through either discovery or manual input, the system generates a corresponding mutant protein sequence by programmatically applying the specified amino acid changes to the WT sequence. This prepares the WT and all mutant sequences for subsequent structural and energetic analysis.

#### Boltz-2 prediction

The primary method for generating structural models and predicting binding affinity utilized Boltz-2. For each protein-drug pair, Boltz-2 simultaneously predicts the 3D protein structure, the ligand binding pose, and the binding affinity. The prediction process is guided by several user-customizable parameters, including recycling steps, sampling steps, diffusion samples, and the number of sequences used in the Multiple Sequence Alignment (MSA). Recycling steps determine how many times the model iteratively revisits and refines the predicted protein–ligand structure. Sampling steps control how long the diffusion-based sampling process runs when generating candidate structures or affinity estimates, while diffusion samples specify how many independent predictions are generated and averaged to improve stability. The MSA depth determines how much evolutionary sequence information is provided to guide the structural prediction.

In BoltzOmics, this inference stage is extended beyond a standard single Boltz-2 run in several ways. First, repeated jobs across related WT and mutant sequences can reuse compatible MSA information when available, reducing repeated preprocessing during large-scale mutant screening. Second, the workflow supports coordinated execution across many protein–drug pairs, including seamless worker assignment for multi-GPU system, so that large mutation panels can be evaluated efficiently. Third, users can optionally provide structural templates and specify pocket residues or mutation-centered regions to guide drug binding.

For the benchmarking analysis in this study, we evaluated three distinct parameter configurations to assess the trade-off between computational cost and accuracy:

**Setting 0 (Boltz-2 default):** 200 affinity sampling steps, 5 diffusion samples.

**Setting 1:** 300 affinity sampling steps, 7 diffusion samples.

**Setting 2:** 400 affinity sampling steps, 9 diffusion samples.

For affinity prediction, Boltz-2 uses a diffusion-based refinement procedure. The affinity settings control how many refinement steps are taken per affinity run (i.e., sampling steps) and how many independent affinity runs are averaged as an ensemble (i.e., diffusion samples). Higher settings therefore increase the amount of affinity refinement and ensemble averaging, which can stabilize the predicted affinity or reveal sensitivity to sampling depth depending on the target and mutation context. Each protein-drug pair was run with three replicas for each of the three settings, totaling nine runs per pair. The final predicted pIC_50_ values reported are the average of the three replicas for a given setting. To evaluate setting dependence without repeatedly recomputing structure, BoltzOmics also implements affinity multi-sampling, in which the structure is generated once and the affinity stage is then reevaluated across multiple settings while reusing the same structural output. In addition to per-setting outputs, the workflow can report a consensus median across settings as a stable summary estimate for each protein–drug–mutation combination, together with the cross-setting variation as a simple measure of setting sensitivity.

In our study, Boltz-2 inference ran on an NVIDIA RTX 5090 GPU with 32 GB of video random-access memory (VRAM), where larger proteins such as hERG and Na_V_1.5 (∼1150 residues) consumed nearly the full capacity, while smaller proteins like CYP3A4 and HER2 required less VRAM. Inference speed differences are minimal; higher settings sample more extensively and may take only a few minutes longer than the default setting. After prediction, BoltzOmics further supports integrated downstream analysis, including WT-versus-mutant drug interaction comparison, PLIP-based protein–ligand interaction summaries,^41^ and optional OpenMM^42^ structural refinement to reduce local steric clashes. These additions make the prediction stage part of a broader mutation-screening workflow rather than an isolated single-query inference step. The amino acid sequences used as inputs to BoltzOmics for the WT proteins are provided in Data S1.

#### Performance comparison against other computational approaches

To contextualize the performance of Boltz-2, we benchmarked it against five comparator methods representing two computational paradigms: structure-based docking and learned drug–target affinity prediction. All methods were evaluated on shared protein–mutation–drug entries and compared against the same curated experimental benchmark panel. Potency-like endpoints were transformed to logarithmic affinity scale, and CYP3A4 clearance endpoints were transformed to logarithmic clearance scale to preserve directional comparability. Results were computed separately by protein and genotype group (WT and mutants) and then summarized across proteins.

For all structure-based docking methods, initial structural files were retrieved from the Protein Data Bank: hERG (PDB: 5VA2),^117^ Na_V_1.5 (PDB: 6LQA),^73^ HER2 (PDB: 3RCD),^118^ and CYP3A4 (PDB: 8SO2).^119^ Rosetta^120^ was first used to model any missing loops, and the complete structures were subsequently relaxed. Mutations were then introduced into the WT structures using UCSF ChimeraX,^121^ followed by another relaxation step with Rosetta to accommodate the structural changes. Because the docking engines differ substantially in pose count and internal search strategy, aggregation was scaled to the effective sampling depth of each method rather than forced to the same absolute or percentage threshold. This reduces sensitivity to noisy outlier poses while preserving the ranking signal produced by each protocol.

**GALigandDock**^16^ was run in *dockflex* mode with Rosetta energy scoring. In this mode, ligand poses are explored with local receptor side-chain flexibility near the binding site, while the receptor backbone is kept fixed. For each protein–ligand pair, 1,000 docked decoys were generated, with the internal genetic algorithm exploring approximately 20 candidate poses per evolutionary cycle, corresponding to roughly 20,000 sampled poses before final decoy selection. For cross-method comparison, the final GALigandDock score for each complex was summarized by averaging the top 10% of scored docked decoys, meaning 100 most favorable docked poses per complex.

**RosettaLigand**^106^ was run using a flexible-receptor RosettaScripts protocol with explicit local receptor flexibility. For each protein–ligand pair, 10,000 docked decoys were generated. The protocol included initial ligand perturbation and placement, iterative high-resolution docking, side-chain repacking near the interface, limited local backbone motion adjacent to the ligand-contact region, and final minimization with interface-energy scoring. To avoid arbitrary post-processing, we systematically screened two-stage aggregation strategies. In each strategy, poses were first prefiltered by total Rosetta energy and then reranked by interface interaction energy. The selected benchmark strategy retained the best 10% of poses by total energy, yielding a prefilter set of 1,000 poses per complex, and then averaged the top 10 interface-ranked poses to obtain the final score.

**AutoDock Vina**^18^ was run using a standard receptor–ligand docking workflow. Structures were prepared in PDBQT format, and grid boxes were centered on the reference ligand binding region for each protein. Docking was performed with exhaustiveness set to 20. For each protein–ligand pair, approximately 200 poses were generated, and the final score for comparison was taken as the average of the top 20 poses per complex.

**DeepDTA**^23^ was run as a dual-branch sequence-based neural network trained on KIBA interactions when a cached model was not already available. The drug branch encoded SMILES tokens and the protein branch encoded amino acid sequences, each followed by stacked one-dimensional convolutional layers and global pooling, with fully connected regression layers for final output. Training used Adam optimization, mean squared error loss, early stopping on validation loss, and an 80/20 split of non-missing KIBA entries in this workflow. Inference produced one predicted affinity value per benchmark pair.

**GraphDTA**^22^ was run as a graph neural network–based drug–target affinity predictor trained on the KIBA benchmark framework, using GINConvNet as the default architecture in this workflow. Drug molecules were represented as molecular graphs derived from SMILES strings, and protein targets were represented by encoded amino acid sequences. Training followed the KIBA fold-setting procedure with mean squared error optimization and checkpoint selection based on test-fold performance. Inference on the benchmark pair list produced one predicted affinity value per protein–drug pair.

#### Binding affinity analysis

Binding affinity for each protein-ligand complex predicted by Boltz-2 was reported as pIC_50_ = -log_10_(IC_50_(M)). We use pIC_50_ because IC_50_ values often span orders of magnitude. The log transform compresses this wide range, makes plots easier to read and compare, and typically yields more linear, well-behaved relationships for correlation and error metrics.

For the drug-metabolizing enzyme CYP3A4, where binding affinity is inversely related to metabolic activity, predicted pIC_50_ values were compared against experimental intrinsic clearance data. The predictive performance was evaluated by comparing predicted values against experimental data collected from published literature (Tables S1–S4). The agreement was quantified using the Pearson correlation coefficient (r), the coefficient of determination (R^2^), and the Root Mean Square Error (RMSE) from a perfect 1:1 correlation. Linear fitting was performed using Orthogonal Distance Regression (ODR), which was chosen over standard linear regression because it accounts for errors in both the experimental and predicted variables, providing a more robust and unbiased fit for this type of comparative analysis.

#### Structural validation

The structural accuracy of the Boltz-2 pipeline was validated by comparing predicted models to experimentally determined structures from the Protein Data Bank (PDB)^122^: hERG-astemizole (PDB: 8ZYO),^105^ hERG-E4031 (PDB: 8ZYP),^105^ Na_V_1.5-quinidine (PDB: 6LQA),^73^ and HER2-JBJ-08-178-01 (PDB: 7JXH).^95^ Accuracy was assessed by calculating the Root Mean Square Deviation (RMSD) between the predicted and experimental structures for the whole protein, the binding site residues (within 5 Å of the ligand), and the ligand pose.

#### Visualization and data output

The pipeline generates a suite of interactive visualizations to facilitate data interpretation, including heatmaps of pIC_50_ values, violin plots of binding affinity distributions, and comparative bar plots. A 3D molecular viewer, utilizing MolViewSpec,^123^ is integrated for real-time exploration of predicted protein-ligand complexes. All results, including predicted pIC_50_ values, confidence metrics (e.g., pTM, ipTM, pLDDT), and downloadable 3D structure files in .pdb format, are organized into a project-specific directory. Final protein structure visualizations and figures for publication were generated using UCSF ChimeraX.^121^

### Quantification and statistical analysis

Predicted and experimental drug-response values were compared using Pearson correlation coefficients, coefficients of determination (R^2^), root mean square error (RMSE), and pairwise ranking accuracy. Linear fitting was performed using orthogonal distance regression to account for uncertainty in both predicted and experimental variables. Pairwise ranking accuracy was calculated by comparing the predicted and experimental ordering of all unordered drug or mutation pairs within each relevant benchmark group. Sample sizes are reported in the Results, figure legends, and Tables S1–S4. Unless otherwise stated, summary statistics in bar plots represent mean values, and error bars represent standard error of the mean.

## References

[1] Evans W.E., McLeod H.L. (2003). Pharmacogenomics — Drug Disposition, Drug Targets, and Side Effects. N. Engl. J. Med..

[2] Roden D.M., Altman R.B., Benowitz N.L., Flockhart D.A., Giacomini K.M., Johnson J.A., Krauss R.M., McLeod H.L., Ratain M.J., Relling M.V. (2006). Pharmacogenomics: Challenges and Opportunities. Ann. Intern. Med..

[3] Weinshilboum R. (2003). Inheritance and Drug Response. N. Engl. J. Med..

[4] Wilke R.A., Lin D.W., Roden D.M., Watkins P.B., Flockhart D., Zineh I., Giacomini K.M., Krauss R.M. (2007). Identifying genetic risk factors for serious adverse drug reactions: current progress and challenges. Nat. Rev. Drug Discov..

[5] Chan H.T., Chin Y.M., Low S.-K. (2019). The Roles of Common Variation and Somatic Mutation in Cancer Pharmacogenomics. Oncol. Ther..

[6] Heller F. (2013). Genetics/genomics and drug effects. Acta Clin. Belg..

[7] Lyumkis D. (2019). Challenges and opportunities in cryo-EM single-particle analysis. J. Biol. Chem..

[8] Altshuler D., Daly M.J., Lander E.S. (2008). Genetic mapping in human disease. Science.

[9] Leshchiner I., Alexa K., Kelsey P., Adzhubei I., Austin-Tse C.A., Cooney J.D., Anderson H., King M.J., Stottmann R.W., Garnaas M.K. (2012). Mutation mapping and identification by whole-genome sequencing. Genome Res..

[10] Friedman R. (2022). Computational studies of protein–drug binding affinity changes upon mutations in the drug target. WIREs Comput. Mol. Sci..

[11] Wang C., Nguyen P.H., Pham K., Huynh D., Le T.N., Wang H., Ren P., Luo R. (2016). Calculating protein–ligand binding affinities with MMPBSA: Method and error analysis. J. Comput. Chem..

[12] Ahn S.-H., Jagger B.R., Amaro R.E. (2020). Ranking of Ligand Binding Kinetics Using a Weighted Ensemble Approach and Comparison with a Multiscale Milestoning Approach. J. Chem. Inf. Model..

[13] Case D.A., Cheatham T.E., Darden T., Gohlke H., Luo R., Merz K.M., Onufriev A., Simmerling C., Wang B., Woods R.J. (2005). The Amber biomolecular simulation programs. J. Comput. Chem..

[14] Wei H., McCammon J.A. (2024). Structure and dynamics in drug discovery. npj Drug Discov..

[15] Verdonk M.L., Cole J.C., Hartshorn M.J., Murray C.W., Taylor R.D. (2003). Improved protein-ligand docking using GOLD. Proteins.

[16] Park H., Zhou G., Baek M., Baker D., DiMaio F. (2021). Force Field Optimization Guided by Small Molecule Crystal Lattice Data Enables Consistent Sub-Angstrom Protein-Ligand Docking. J. Chem. Theory Comput..

[17] Friesner R.A., Banks J.L., Murphy R.B., Halgren T.A., Klicic J.J., Mainz D.T., Repasky M.P., Knoll E.H., Shelley M., Perry J.K. (2004). Glide: A New Approach for Rapid, Accurate Docking and Scoring. 1. Method and Assessment of Docking Accuracy. J. Med. Chem..

[18] Eberhardt J., Santos-Martins D., Tillack A.F., Forli S. (2021). AutoDock Vina 1.2.0: New Docking Methods, Expanded Force Field, and Python Bindings. J. Chem. Inf. Model..

[19] Harris B.J., Nguyen P.T., Zhou G., Wulff H., DiMaio F., Yarov-Yarovoy V. (2024). Toward high-resolution modeling of small molecule-ion channel interactions. Front. Pharmacol..

[20] Nivatya H.K., Singh A., Kumar N., Sonam, Sharma L., Singh V., Mishra R., Gaur N., Mishra A.K. (2025). Assessing molecular docking tools: understanding drug discovery and design. Futur. J. Pharm. Sci..

[21] Buccheri R., Rescifina A. (2025). High-Throughput, High-Quality: Benchmarking GNINA and AutoDock Vina for Precision Virtual Screening Workflow. Molecules.

[22] Nguyen T., Le H., Quinn T.P., Nguyen T., Le T.D., Venkatesh S. (2021). GraphDTA: predicting drug–target binding affinity with graph neural networks. Bioinformatics.

[23] Öztürk H., Özgür A., Ozkirimli E. (2018). DeepDTA: deep drug–target binding affinity prediction. Bioinformatics.

[24] Zeng X., Li S.-J., Lv S.-Q., Wen M.-L., Li Y. (2024). A comprehensive review of the recent advances on predicting drug-target affinity based on deep learning. Front. Pharmacol..

[25] Jumper J., Evans R., Pritzel A., Green T., Figurnov M., Ronneberger O., Tunyasuvunakool K., Bates R., Žídek A., Potapenko A. (2021). Highly accurate protein structure prediction with AlphaFold. Nature.

[26] Abramson J., Adler J., Dunger J., Evans R., Green T., Pritzel A., Ronneberger O., Willmore L., Ballard A.J., Bambrick J. (2024). Accurate structure prediction of biomolecular interactions with AlphaFold 3. Nature.

[27] Krishna R., Wang J., Ahern W., Sturmfels P., Venkatesh P., Kalvet I., Lee G.R., Morey-Burrows F.S., Anishchenko I., Humphreys I.R. (2024). Generalized biomolecular modeling and design with RoseTTAFold All-Atom. Science.

[28] Passaro S., Corso G., Wohlwend J., Reveiz M., Thaler S., Somnath V.R., Getz N., Portnoi T., Roy J., Stark H. (2025). Boltz-2: Towards Accurate and Efficient Binding Affinity Prediction. bioRxiv.

[29] Roden D.M. (2004). Drug-Induced Prolongation of the QT Interval. N. Engl. J. Med..

[30] Fenichel R.R., Malik M., Antzelevitch C., Sanguinetti M., Roden D.M., Priori S.G., Ruskin J.N., Lipicky R.J., Cantilena L.R., Independent Academic Task Force (2004). Drug-Induced Torsades de Pointes and Implications for Drug Development. J. Cardiovasc. Electrophysiol..

[31] Sanguinetti M.C., Jiang C., Curran M.E., Keating M.T. (1995). A mechanistic link between an inherited and an acquird cardiac arrthytmia: *HERG* encodes the IKr potassium channel. Cell.

[32] Chaudhary K.W., Clancy C.E., Yang P.C., Pierson J.B., Goldin A.L., Koerner J.E., Wisialowski T.A., Valentin J.P., Imredy J.P., Lagrutta A. (2024). An overview of drug-induced sodium channel blockade and changes in cardiac conduction: Implications for drug safety. Clin. Transl. Sci..

[33] Alsaloum M., Dib-Hajj S.D., Page D.A., Ruben P.C., Krainer A.R., Waxman S.G. (2025). Voltage-gated sodium channels in excitable cells as drug targets. Nat. Rev. Drug Discov..

[34] Swain S.M., Shastry M., Hamilton E. (2023). Targeting HER2-positive breast cancer: advances and future directions. Nat. Rev. Drug Discov..

[35] Cocco E., Lopez S., Santin A.D., Scaltriti M. (2019). Prevalence and role of HER2 mutations in cancer. Pharmacol. Ther..

[36] Zhou S.-F. (2008). Drugs behave as substrates, inhibitors and inducers of human cytochrome P450 3A4. Curr. Drug Metab..

[37] Haddad A., Davis M., Lagman R. (2007). The pharmacological importance of cytochrome CYP3A4 in the palliation of symptoms: review and recommendations for avoiding adverse drug interactions. Support. Care Cancer.

[38] Ng P.C., Henikoff S. (2003). SIFT: Predicting amino acid changes that affect protein function. Nucleic Acids Res..

[39] Adzhubei I., Jordan D.M., Sunyaev S.R. (2013). Predicting functional effect of human missense mutations using PolyPhen-2. Curr. Protoc. Hum. Genet..

[40] Cheng J., Novati G., Pan J., Bycroft C., Žemgulytė A., Applebaum T., Pritzel A., Wong L.H., Zielinski M., Sargeant T. (2023). Accurate proteome-wide missense variant effect prediction with AlphaMissense. Science.

[41] Salentin S., Schreiber S., Haupt V.J., Adasme M.F., Schroeder M. (2015). PLIP: fully automated protein–ligand interaction profiler. Nucleic Acids Res..

[42] Eastman P., Pande V. (2010). OpenMM: A Hardware Independent Framework for Molecular Simulations. Comput. Sci. Eng..

[43] Orvos P., Kohajda Z., Szlovák J., Gazdag P., Árpádffy-Lovas T., Tóth D., Geramipour A., Tálosi L., Jost N., Varró A., Virág L. (2019). Evaluation of Possible Proarrhythmic Potency: Comparison of the Effect of Dofetilide, Cisapride, Sotalol, Terfenadine, and Verapamil on hERG and Native IKr Currents and on Cardiac Action Potential. Toxicol. Sci..

[44] Duan J.j., Ma J.h., Zhang P.h., Wang X.p., Zou A.r., Tu D.n. (2007). Verapamil blocks HERG channel by the helix residue Y652 and F656 in the S6 transmembrane domain. Acta Pharmacol. Sin..

[45] Saxena P., Zangerl-Plessl E.M., Linder T., Windisch A., Hohaus A., Timin E., Hering S., Stary-Weinzinger A. (2016). New potential binding determinant for hERG channel inhibitors. Sci. Rep..

[46] Chiu P.J.S., Marcoe K.F., Bounds S.E., Lin C.H., Feng J.J., Lin A., Cheng F.C., Crumb W.J., Mitchell R. (2004). Validation of a [3H]astemizole binding assay in HEK293 cells expressing HERG K+ channels. J. Pharmacol. Sci..

[47] Zhang Y., Colenso C.K., El Harchi A., Cheng H., Witchel H.J., Dempsey C.E., Hancox J.C. (2016). Interactions between amiodarone and the hERG potassium channel pore determined with mutagenesis and in silico docking. Biochem. Pharmacol..

[48] Park S.-J., Kim K.-S., Kim E.-J. (2008). Blockade of HERG K+ channel by an antihistamine drug brompheniramine requires the channel binding within the S6 residue Y652 and F656. J. Appl. Toxicol..

[49] Xing J., Ma J., Zhang P., Fan X. (2010). Block effect of capsaicin on hERG potassium currents is enhanced by S6 mutation at Y652. Eur. J. Pharmacol..

[50] Rampe D., Roy M.L., Dennis A., Brown A.M. (1997). A mechanism for the proarrhythmic effects of cisapride (Propulsid): high affinity blockade of the human cardiac potassium channel HERG. FEBS Lett..

[51] Ficker E., Jarolimek W., Kiehn J., Baumann A., Brown A.M. (1998). Molecular Determinants of Dofetilide Block of HERG K+ Channels. Circ. Res..

[52] Li P., Sun H.f., Zhou P.z., Ma C.y., Hu G.y., Jiang H.l., Li M., Liu H., Gao Z.b. (2012). Comparison of the effects of DC031050, a class III antiarrhythmic agent, on hERG channel and three neuronal potassium channels. Acta Pharmacol. Sin..

[53] Zhou Z., Gong Q., Ye B., Fan Z., Makielski J.C., Robertson G.A., January C.T. (1998). Properties of HERG channels stably expressed in HEK 293 cells studied at physiological temperature. Biophys. J..

[54] Ishii K., Nagai M., Takahashi M., Endoh M. (2003). Dissociation of E-4031 from the HERG channel caused by mutations of an amino acid results in greater block at high stimulation frequency. Cardiovasc. Res..

[55] Melgari D., Zhang Y., El Harchi A., Dempsey C.E., Hancox J.C. (2015). Molecular basis of hERG potassium channel blockade by the class Ic antiarrhythmic flecainide. J. Mol. Cell. Cardiol..

[56] Suessbrich H., Schönherr R., Heinemann S.H., Attali B., Lang F., Busch A.E. (1997). The inhibitory effect of the antipsychotic drug haloperidol on HERG potassium channels expressed in Xenopus oocytes. Br. J. Pharmacol..

[57] Zhang P., Xing J., Luo A., Feng J., Liu Z., Gao C., Ma J. (2013). Blockade of the human ether-a-go-go-related gene potassium channel by ketamine. J. Pharm. Pharmacol..

[58] Alexandrou A.J., Duncan R.S., Sullivan A., Hancox J.C., Leishman D.J., Witchel H.J., Leaney J.L. (2006). Mechanism of hERG K+ channel blockade by the fluoroquinolone antibiotic moxifloxacin. Br. J. Pharmacol..

[59] Chen X., Cass J.D., Bradley J.A., Dahm C.M., Sun Z., Kadyszewski E., Engwall M.J., Zhou J. (2005). QT prolongation and proarrhythmia by moxifloxacin: concordance of preclinical models in relation to clinical outcome. Br. J. Pharmacol..

[60] Kushida S., Ogura T., Komuro I., Nakaya H. (2002). Inhibitory effect of the class III antiarrhythmic drug nifekalant on HERG channels: mode of action. Eur. J. Pharmacol..

[61] Walker B.D., Valenzuela S.M., Singleton C.B., Tie H., Bursill J.A., Wyse K.R., Qiu M.R., Breit S.N., Campbell T.J. (1999). Inhibition of HERG channels stably expressed in a mammalian cell line by the antianginal agent perhexiline maleate. Br. J. Pharmacol..

[62] Wang N., Ma J.H., Zhang P.H. (2013). a state-dependent blocker, inhibits HERG channels by helix residue Y652 and F656 in the S6 transmembrane domain. J. Pharmacol. Sci..

[63] Yan M., Fan P., Shi Y., Feng L., Wang J., Zhan G., Li B. (2016). Stereoselective Blockage of Quinidine and Quinine in the hERG Channel and the Effect of Their Rescue Potency on Drug-Induced hERG Trafficking Defect. Int. J. Mol. Sci..

[64] Paul A.A., Witchel H.J., Hancox J.C. (2002). Inhibition of the current of heterologously expressed HERG potassium channels by flecainide and comparison with quinidine, propafenone and lignocaine. Br. J. Pharmacol..

[65] Perrin M.J., Kuchel P.W., Campbell T.J., Vandenberg J.I. (2008). Drug binding to the inactivated state is necessary but not sufficient for high-affinity binding to human ether-à-go-go-related gene channels. Mol. Pharmacol..

[66] Zhang S., Zhou Z., Gong Q., Makielski J.C., January C.T. (1999). Mechanism of block and identification of the verapamil binding domain to HERG potassium channels. Circ. Res..

[67] Johnson A.A., Trudeau M.C. (2024). Inhibition of hERG K channels by verapamil at physiological temperature: Implications for the CiPA initiative. J. Pharmacol. Toxicol. Methods.

[68] Yang P.-C., Belardinelli L., Clancy C.E. (2024). Mechanisms of Chemical Atrial Defibrillation by Flecainide and Ibutilide. JACC. Clin. Electrophysiol..

[69] Docken S.S., Clancy C.E., Lewis T.J. (2021). Rate-dependent effects of lidocaine on cardiac dynamics: Development and analysis of a low-dimensional drug-channel interaction model. PLoS Comput. Biol..

[70] Docken S.S., Clancy C.E., Lewis T.J. (2023). Rate-dependent effects of state-specific sodium channel blockers in cardiac tissue: Insights from idealized models. J. Theor. Biol..

[71] Moreno J.D., Zhu Z.I., Yang P.C., Bankston J.R., Jeng M.T., Kang C., Wang L., Bayer J.D., Christini D.J., Trayanova N.A. (2011). A computational model to predict the effects of class I anti-arrhythmic drugs on ventricular rhythms. Sci. Transl. Med..

[72] Yang P.-C., Giles W.R., Belardinelli L., Clancy C.E. (2021). Mechanisms of flecainide induced negative inotropy: An in silico study. J. Mol. Cell. Cardiol..

[73] Li Z., Jin X., Wu T., Huang G., Wu K., Lei J., Pan X., Yan N. (2021). Structural Basis for Pore Blockade of the Human Cardiac Sodium Channel Nav1.5 by the Antiarrhythmic Drug Quinidine. Angew. Chem. Int. Ed..

[74] Bankston J.R., Kass R.S. (2010). Molecular determinants of local anesthetic action of beta-blocking drugs: Implications for therapeutic management of long QT syndrome variant 3. J. Mol. Cell. Cardiol..

[75] Bräu M.E., Vogel W., Hempelmann G. (1998). Fundamental properties of local anesthetics: half-maximal blocking concentrations for tonic block of Na+ and K+ channels in peripheral nerve. Anesth. Analg..

[76] Poulin H., Bruhova I., Timour Q., Theriault O., Beaulieu J.M., Frassati D., Chahine M. (2014). Fluoxetine blocks Nav1.5 channels via a mechanism similar to that of class 1 antiarrhythmics. Mol. Pharmacol..

[77] Liu H., Atkins J., Kass R.S. (2003). Common Molecular Determinants of Flecainide and Lidocaine Block of Heart Na+ Channels. J. Gen. Physiol..

[78] Bean B.P., Cohen C.J., Tsien R.W. (1983). Lidocaine block of cardiac sodium channels. J. Gen. Physiol..

[79] Kambouris N.G., Nuss H.B., Johns D.C., Marbán E., Tomaselli G.F., Balser J.R. (2000). A revised view of cardiac sodium channel ‘blockade’ in the long-QT syndrome. J. Clin. Investig..

[80] Wang D.W., Mistry A.M., Kahlig K.M., Kearney J.A., Xiang J., George A.L. (2010). Propranolol Blocks Cardiac and Neuronal Voltage-Gated Sodium Channels. Front. Pharmacol..

[81] Crumb W.J., Vicente J., Johannesen L., Strauss D.G. (2016). An evaluation of 30 clinical drugs against the comprehensive *in vitro* proarrhythmia assay (CiPA) proposed ion channel panel. J. Pharmacol. Toxicol. Methods.

[82] Finkel A., Wittel A., Yang N., Handran S., Hughes J., Costantin J. (2006). Population Patch Clamp Improves Data Consistency and Success Rates in the Measurement of Ionic Currents. SLAS Discov..

[83] O’Leary M.E., Chahine M. (2024). Regulation of cardiac Nav1.5 channel gating and drug binding by the beta-1 subunit. Biophys. J..

[84] Zhou S.-F., Xue C.C., Yu X.-Q., Li C., Wang G. (2007). Clinically important drug interactions potentially involving mechanism-based inhibition of cytochrome P450 3A4 and the role of therapeutic drug monitoring. Ther. Drug Monit..

[85] Werk A.N., Cascorbi I. (2014). Functional gene variants of CYP3A4. Clin. Pharmacol. Ther..

[86] Zhou X.-Y., Hu X.X., Wang C.C., Lu X.R., Chen Z., Liu Q., Hu G.X., Cai J.P. (2019). Enzymatic Activities of CYP3A4 Allelic Variants on Quinine 3-Hydroxylation In Vitro. Front. Pharmacol..

[87] Tang P.F., Zheng X., Hu X.X., Yang C.C., Chen Z., Qian J.C., Cai J.P., Hu G.X. (2020). Functional Measurement of CYP2C9 and CYP3A4 Allelic Polymorphism on Sildenafil Metabolism. Drug Des. Devel. Ther.

[88] Usmani K.A., Cho T.M., Rose R.L., Hodgson E. (2006). Inhibition of the Human Liver Microsomal and Human Cytochrome P450 1A2 and 3A4 Metabolism of Estradiol by Deployment-Related and Other Chemicals. Drug Metab. Dispos..

[89] Zhang F., Song L., Wang R., Zhao B., Huang J., Wu L., Fan Y., Lin H., Jiang Z., Yang X. (2025). Functional Imaging of CYP3A4 at Multiple Dimensions Using an AI-Driven High Performance Fluorogenic Substrate. Small.

[90] Pan L., Li J., Xu Q., Gao Z., Yang M., Wu X., Li X. (2024). HER2/PI3K/AKT pathway in HER2-positive breast cancer: A review. Medicine (Baltimore).

[91] Wang Z., Wang W., Xu S., Wang S., Tu Y., Xiong Y., Mei J., Wang C. (2016). The role of MAPK signaling pathway in the Her-2-positive meningiomas. Oncol. Rep..

[92] Johnston S.R.D., Leary A. (2006). Lapatinib: a novel EGFR/HER2 tyrosine kinase inhibitor for cancer. Drugs Today (Barc).

[93] Chan A., Moy B., Mansi J., Ejlertsen B., Holmes F.A., Chia S., Iwata H., Gnant M., Loibl S., Barrios C.H. (2021). Final Efficacy Results of Neratinib in HER2-positive Hormone Receptor-positive Early-stage Breast Cancer From the Phase III ExteNET Trial. Clin. Breast Cancer.

[94] Olson D., Taylor J., Willis K., Hensley K., Allred S., Zaval M., Farr L., Thurman R., Jain N., Hein R. (2023). HER2-Selective and Reversible Tyrosine Kinase Inhibitor Tucatinib Potentiates the Activity of T-DM1 in Preclinical Models of HER2-positive Breast Cancer. Cancer Res. Commun..

[95] Son J., Jang J., Beyett T.S., Eum Y., Haikala H.M., Verano A., Lin M., Hatcher J.M., Kwiatkowski N.P., Eser P.Ö. (2022). A Novel HER2-Selective Kinase Inhibitor Is Effective in HER2 Mutant and Amplified Non–Small Cell Lung Cancer. Cancer Res..

[96] Cocco E., Javier Carmona F., Razavi P., Won H.H., Cai Y., Rossi V., Chan C., Cownie J., Soong J., Toska E. (2018). Neratinib is effective in breast tumors bearing both amplification and mutation of ERBB2 (HER2). Sci. Signal..

[97] Harada Y., Sato A., Nakamura H., Kai K., Kitamura S., Nakamura T., Kurihara Y., Ikeda S., Sueoka E., Kimura S., Sueoka-Aragane N. (2023). Anti-cancer effect of afatinib, dual inhibitor of HER2 and EGFR, on novel mutation HER2 E401G in models of patient-derived cancer. BMC Cancer.

[98] Schroeder R., Stevens C., Sridhar J. (2014). Small Molecule Tyrosine Kinase Inhibitors of ErbB2/HER2/Neu in the Treatment of Aggressive Breast Cancer. Molecules.

[99] Collins D.M., Conlon N.T., Kannan S., Verma C.S., Eli L.D., Lalani A.S., Crown J. (2019). Preclinical Characteristics of the Irreversible Pan-HER Kinase Inhibitor Neratinib Compared with Lapatinib: Implications for the Treatment of HER2-Positive and HER2-Mutated Breast Cancer. Cancers.

[100] Cai X., Zhai H.X., Wang J., Forrester J., Qu H., Yin L., Lai C.J., Bao R., Qian C. (2010). Discovery of 7-(4-(3-ethynylphenylamino)-7-methoxyquinazolin-6-yloxy)-N-hydroxyheptanamide (CUDc-101) as a potent multi-acting HDAC, EGFR, and HER2 inhibitor for the treatment of cancer. J. Med. Chem..

[101] Ashar Y.V., Zhou J., Gupta P., Teng Q.X., Lei Z.N., Reznik S.E., Lusvarghi S., Wurpel J., Ambudkar S.V., Chen Z.S. (2020). BMS-599626, a Highly Selective Pan-HER Kinase Inhibitor, Antagonizes ABCG2-Mediated Drug Resistance. Cancers.

[102] Zoeller R.A., Geoghegan-Barek K. (2019). A cell-based high-throughput screen identifies tyrphostin AG 879 as an inhibitor of animal cell phospholipid and fatty acid biosynthesis. Biochem. Biophys. Rep..

[103] Tang P.C., Sun L., McMahon G. (1998). McMahon G. 3-heteroaryl-2-indolinone Compounds for the Treatment of Disease. U.S. Patent No.

[104] Irie H., Ito K., Fujioka Y., Oguchi K., Fujioka A., Hashimoto A., Ohsawa H., Tanaka K., Funabashi K., Araki H. (2019). TAS0728, A Covalent-binding, HER2-selective Kinase Inhibitor Shows Potent Antitumor Activity in Preclinical Models. Mol. Cancer Ther..

[105] Miyashita Y., Moriya T., Kato T., Kawasaki M., Yasuda S., Adachi N., Suzuki K., Ogasawara S., Saito T., Senda T., Murata T. (2024). Improved higher resolution cryo-EM structures reveal the binding modes of hERG channel inhibitors. Structure.

[106] Davis I.W., Baker D. (2009). RosettaLigand docking with full ligand and receptor flexibility. J. Mol. Biol..

[107] Gaulton A., Bellis L.J., Bento A.P., Chambers J., Davies M., Hersey A., Light Y., McGlinchey S., Michalovich D., Al-Lazikani B., Overington J.P. (2012). ChEMBL: a large-scale bioactivity database for drug discovery. Nucleic Acids Res..

[108] Liu T., Hwang L., Burley S.K., Nitsche C.I., Southan C., Walters W.P., Gilson M.K. (2025). BindingDB in 2024: a FAIR knowledgebase of protein-small molecule binding data. Nucleic Acids Res..

[109] Kim S. (2021). Exploring Chemical Information in PubChem. Curr. Protoc..

[110] Ngo K., Yang P.-C., Yarov-Yarovoy V., Clancy C.E., Vorobyov I. (2025). Harnessing AlphaFold to reveal hERG channel conformational state secrets. eLife.

[111] Li J., Jiang H., Ma W., Bi X., Chen R., Lu W., Cai Q., Yang F., Wei Z., Zhang S. (2025). Geometric Deep Learning for Protein-Ligand Affinity Prediction with Hybrid Message Passing Strategies. IEEE J. Biomed. Health Inform..

[112] Li J., Bi X., Ma W., Jiang H., Liu S., Lu Y., Wei Z., Zhang S. (2025). MHAN-DTA: A Multiscale Hybrid Attention Network for Drug-Target Affinity Prediction. IEEE J. Biomed. Health Inform..

[113] Bick A.G., Metcalf G.A., Mayo K.R., Lichtenstein L., Rura S., Carroll R.J., Musick A., Linder J.E., Jordan I.K., Nagar S.D. (2024). Genomic data in the All of Us Research Program. Nature.

[114] UniProt Consortium (2025). UniProt: the Universal Protein Knowledgebase in 2025. Nucleic Acids Res..

[115] Stephenson J.D., Totoo P., Burke D., Jänes J., Beltrao P., Martin M. (2024). ProtVar: mapping and contextualizing human missense variation. Nucleic Acids Res..

[116] Landrum M.J., Lee J.M., Benson M., Brown G.R., Chao C., Chitipiralla S., Gu B., Hart J., Hoffman D., Jang W. (2018). ClinVar: improving access to variant interpretations and supporting evidence. Nucleic Acids Res..

[117] Wang W., MacKinnon R. (2017). Structure of the Open Human Ether-à-go-go-Related K+ Channel hERG. Cell.

[118] Ishikawa T., Seto M., Banno H., Kawakita Y., Oorui M., Taniguchi T., Ohta Y., Tamura T., Nakayama A., Miki H. (2011). Design and synthesis of novel human epidermal growth factor receptor 2 (HER2)/epidermal growth factor receptor (EGFR) dual inhibitors bearing a pyrrolo[3,2-d]pyrimidine scaffold. J. Med. Chem..

[119] Sevrioukova I.F. (2023). Interaction of CYP3A4 with caffeine: First insights into multiple substrate binding. J. Biol. Chem..

[120] Fleishman S.J., Leaver-Fay A., Corn J.E., Strauch E.M., Khare S.D., Koga N., Ashworth J., Murphy P., Richter F., Lemmon G. (2011). RosettaScripts: A Scripting Language Interface to the Rosetta Macromolecular Modeling Suite. PLoS One.

[121] Pettersen E.F., Goddard T.D., Huang C.C., Meng E.C., Couch G.S., Croll T.I., Morris J.H., Ferrin T.E. (2021). UCSF ChimeraX: Structure visualization for researchers, educators, and developers. Protein Sci..

[122] Berman H.M., Westbrook J., Feng Z., Gilliland G., Bhat T.N., Weissig H., Shindyalov I.N., Bourne P.E. (2000). The Protein Data Bank. Nucleic Acids Res..

[123] Midlik A., Bittrich S., Fleming J.R., Nair S., Velankar S., Burley S.K., Young J.Y., Vallat B., Sehnal D. (2025). MolViewSpec: a Mol∗ extension for describing and sharing molecular visualizations. Nucleic Acids Res..

