# Supplementary material for: BoltzOmics: Predicting genetic variant effects on drug binding with Boltz-2
Source: iScience. 2026 Jul 16;29(8):116797. doi: 10.1016/j.isci.2026.116797 (PMC13401010; doi:10.1016/j.isci.2026.116797)
Supplement: Document S1. Figures S1–S15 and Tables S1–S4 [file mmc1.pdf]

iScience, Volume *29*

Supplemental information

BoltzOmics: Predicting genetic variant effects on drug binding with Boltz-2

Khoa Ngo, Kermit L. Carraway, Colleen E. Clancy, and Hajar Amini

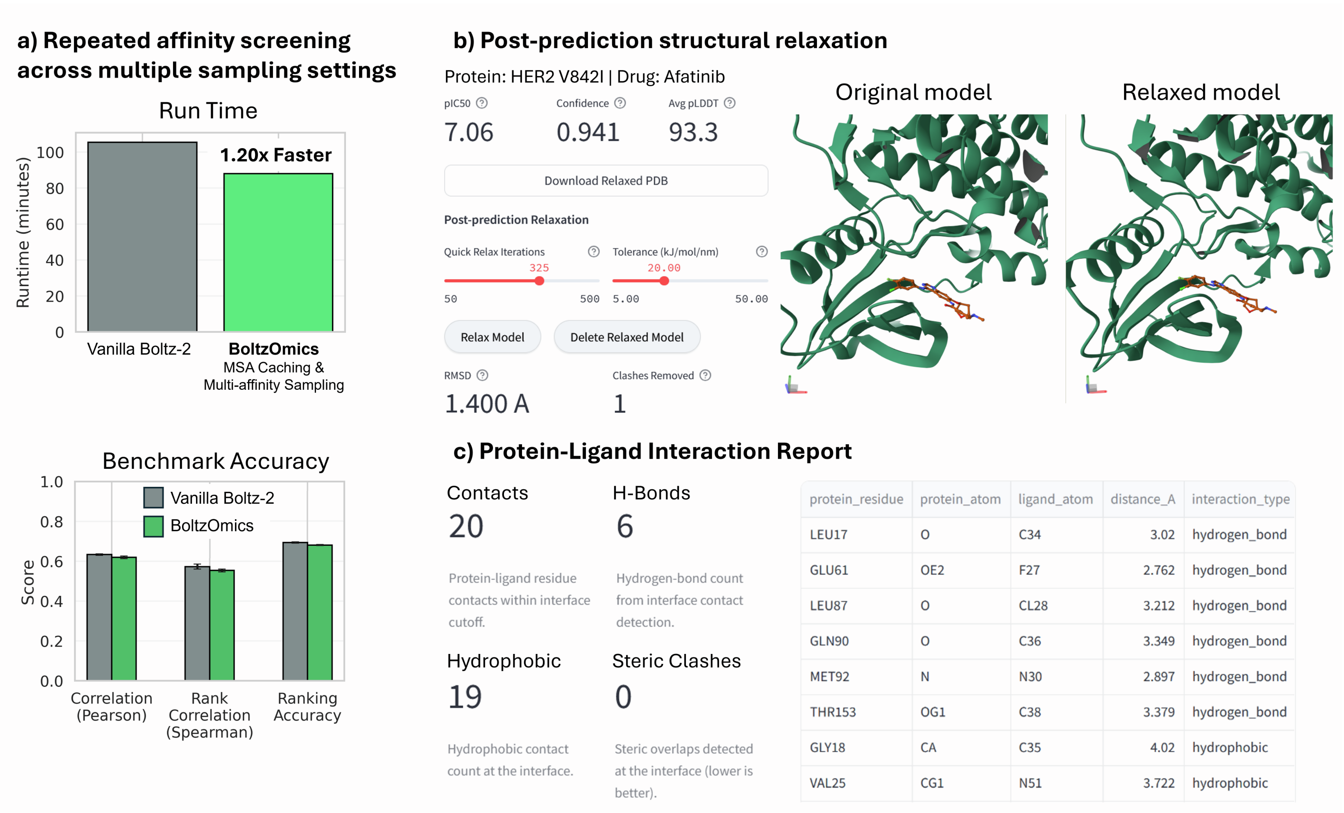

Figure S1. Examples of workflow-level capabilities implemented in BoltzOmics not available in vanilla Boltz-2. (a) Example benchmark on the HER2 panel showing repeated affinity screening across three sampling settings. Using affinity multi-sampling together with MSA caching reduced runtime from 105.5 min for three separate manual runs to 88.1 min in the integrated workflow (1.20× faster; 16.5% runtime reduction) while maintaining comparable accuracy within error on the shared evaluated set (35 matched mutant-drug pairs). Data are presented as mean values with standard error of the mean. **(b)** Post-prediction structural relaxation within the interface, shown here for HER2 V842I bound to afatinib, including side-by-side visualization of the original and relaxed models together with refinement metrics. **(c)** Integrated protein–ligand interaction report, including contact counts, hydrogen bonds, hydrophobic interactions, steric clashes, and a residue-level interaction table. Together, these panels illustrate how BoltzOmics extends Boltz-2 from single-query inference to a more complete screening workflow that supports repeated execution, structural inspection, and downstream interpretation.

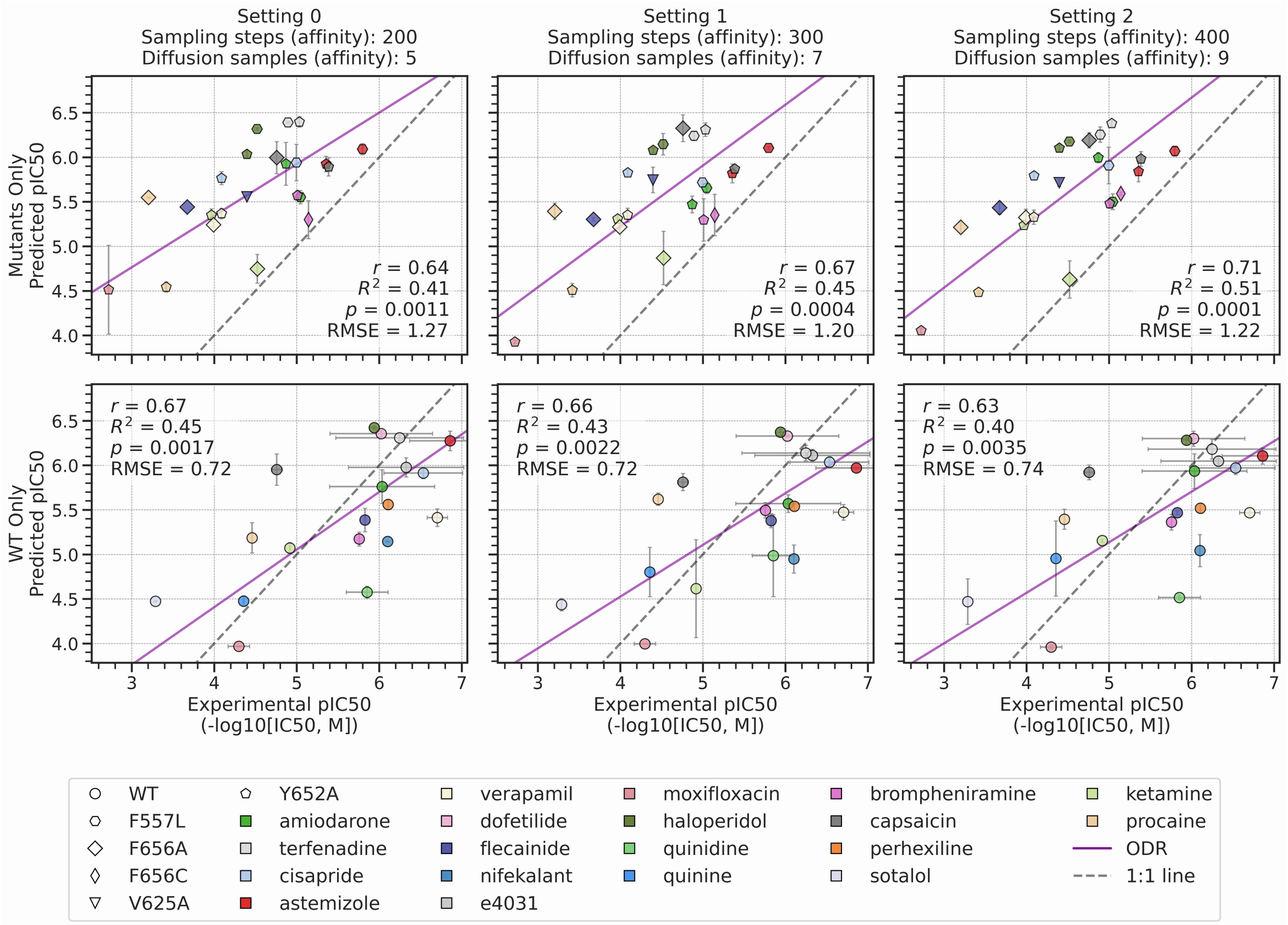

Figure S2. Boltz-2 prediction performance for hERG channel variants across multiple sampling settings. Scatter plots comparing predicted pIC_{50} values against experimental pIC_{50} data for hERG WT and five mutant variants (F557L, F656A, F656C, V625A, Y652A) across three sampling parameter configurations. Each point represents a protein-drug combination from a panel of 20 compounds, including known hERG blockers and cardiac-active drugs, and shows the mean predicted pIC_{50} value with error bars indicating the standard error of the mean. The purple line represents orthogonal distance regression (ODR) fit, while the dashed gray line indicates 1-to-1 correlation. Pearson correlation coefficients (r), coefficient of determination (R^2), and root mean square error (RMSE, representing the average deviation of predictions from perfect correlation) are displayed for each variant-setting combination. WT hERG shows moderately-strong correlations (r = 0.63-0.67) with low RMSE values (0.72-0.74), while mutant variants demonstrate higher correlations (r = 0.64-0.71) but increased RMSE values (1.20-1.27), indicating better relative ranking ability but reduced absolute accuracy for altered channel structures. Performance remains consistent across sampling settings, suggesting robustness of the underlying structure-activity relationships.

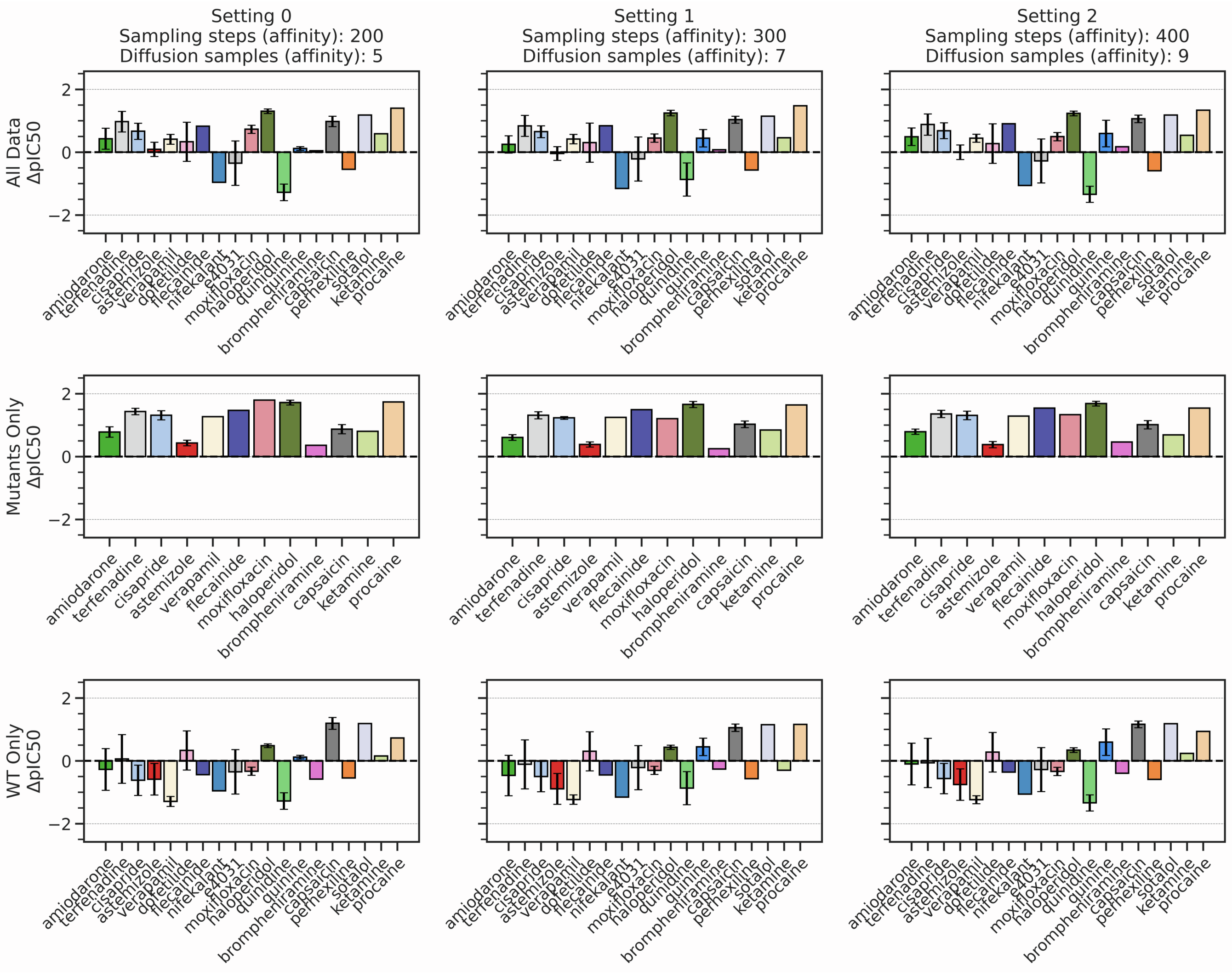

Figure S3. Drug-specific prediction errors for hERG variants across sampling conditions. Bar plots showing prediction errors (ΔpIC_{50} = predicted - experimental) for individual drugs across all hERG protein variants and three sampling settings, with drugs ordered from highest to lowest molecular weight. Bars represent mean ΔpIC_{50} values, with error bars indicating the standard error of the mean. The top panel displays errors aggregated across all protein variants for each drug, the middle panel shows errors for mutant variants only, and the bottom panel presents errors for WT hERG only. Error bars represent standard error across variants (top and middle panels) or technical replicates (bottom panel). The analysis reveals drug-specific biases, with compounds like haloperidol and sotalol showing consistently larger deviations from experimental values, while drugs such as amiodarone and cisapride demonstrate more accurate predictions. The consistent patterns across sampling settings indicate reproducible drug-specific prediction challenges rather than parameter-dependent artifacts, suggesting intrinsic limitations in modeling certain drug-channel interaction mechanisms.

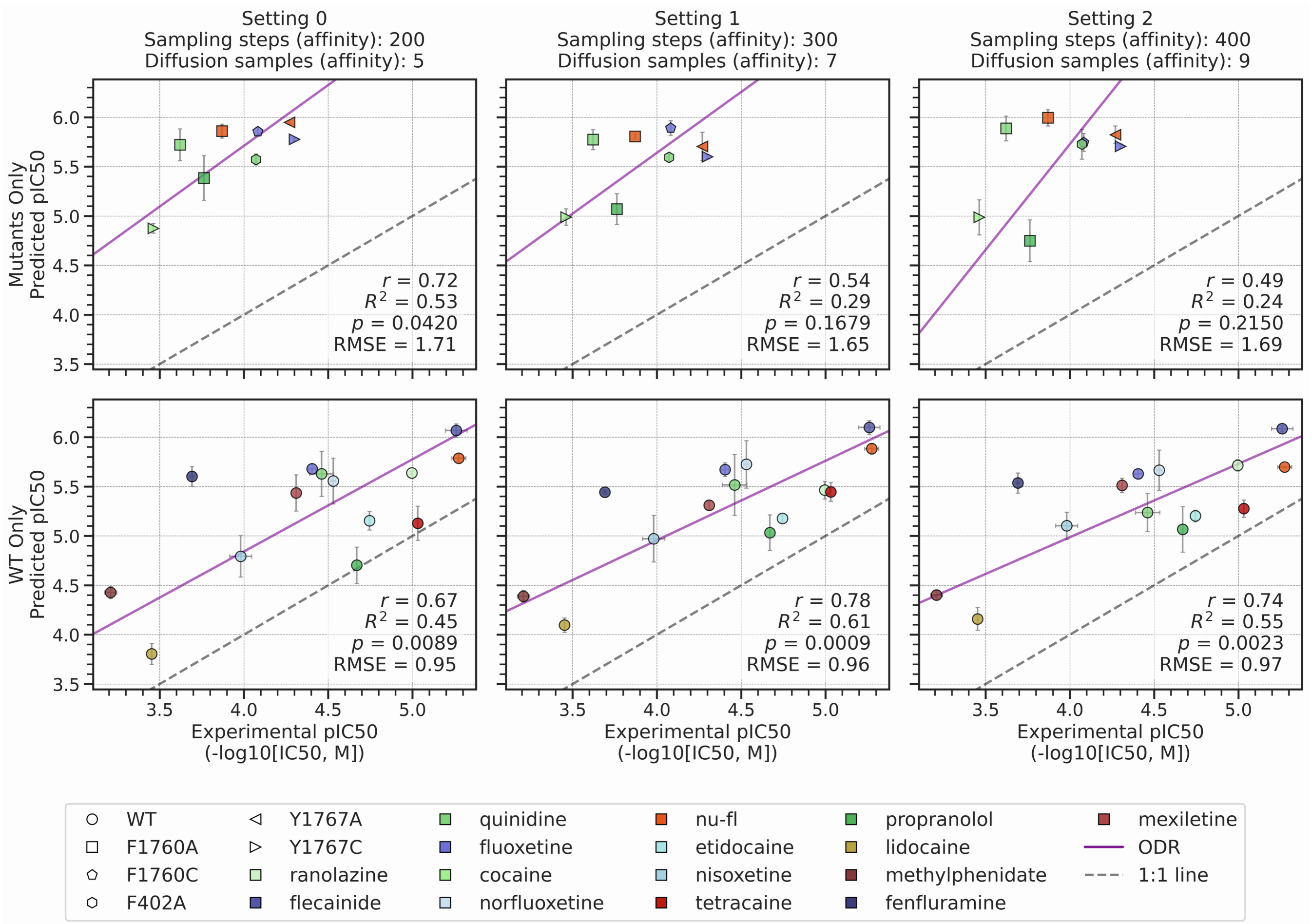

Figure S4. Boltz-2 prediction performance for $Na_V1.5$ sodium channel variants across multiple sampling settings. Scatter plots comparing predicted pIC_{50} values against experimental pIC_{50} data for $Na_V1.5$ WT and five mutant variants (F1760A, F1760C, F402A, Y1767A, Y1767C) across three sampling parameter configurations. Each point represents a protein-drug combination from a panel of 18 compounds including, sodium channel blockers, local anesthetics, and antiarrhythmic drugs, and shows the mean predicted pIC_{50} value with error bars indicating the standard error of the mean. The purple line represents orthogonal distance regression (ODR) fit, while the dashed gray line indicates perfect correlation (1:1). Pearson correlation coefficients (r), coefficient of determination (R^2), and root mean square error (RMSE) are displayed for each variant-setting combination. WT NaV1.5 shows the strongest correlations (r = 0.67-0.78) with moderate RMSE values (0.95-0.97), while mutant variants demonstrate more variable performance with correlations ranging from 0.49-0.72 and higher RMSE values (1.65-1.71).

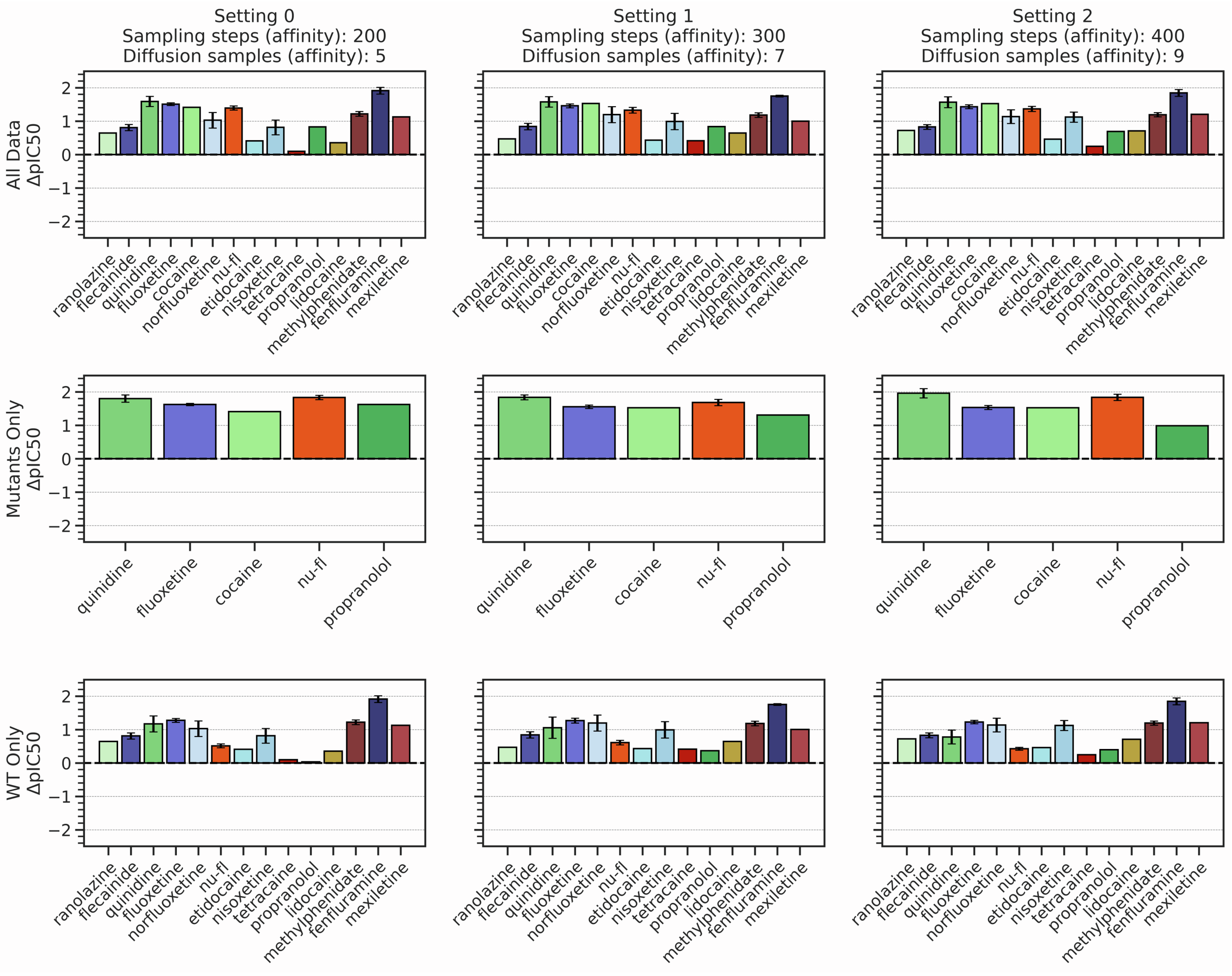

Figure S5. Drug-specific prediction errors for $Na_V1.5$ variants across sampling conditions. Bar plots showing prediction errors (ΔpIC_{50} = predicted - experimental) for individual drugs across all $Na_V1.5$ protein variants and three sampling settings, with drugs ordered from highest to lowest molecular weight. Bars represent mean ΔpIC_{50} values, with error bars indicating the standard error of the mean. The top panel displays errors aggregated across all protein variants for each drug, the middle panel shows errors for mutant variants only, and the bottom panel presents errors for WT $Na_V1.5$ only. Error bars represent standard error across variants (top and middle panels) or technical replicates (bottom panel). The analysis reveals overestimation patterns across most compounds, with local anesthetics like lidocaine and cocaine showing relatively consistent predictions across variants. Antiarrhythmic drugs including quinidine and flecainide demonstrate variable performance, while compounds like mexiletine and fentanyl exhibit larger deviations. The consistent error patterns across sampling settings indicate reproducible compound-specific prediction challenges, suggesting intrinsic limitations in modeling certain drug-channel interaction mechanisms rather than parameter-dependent artifacts.

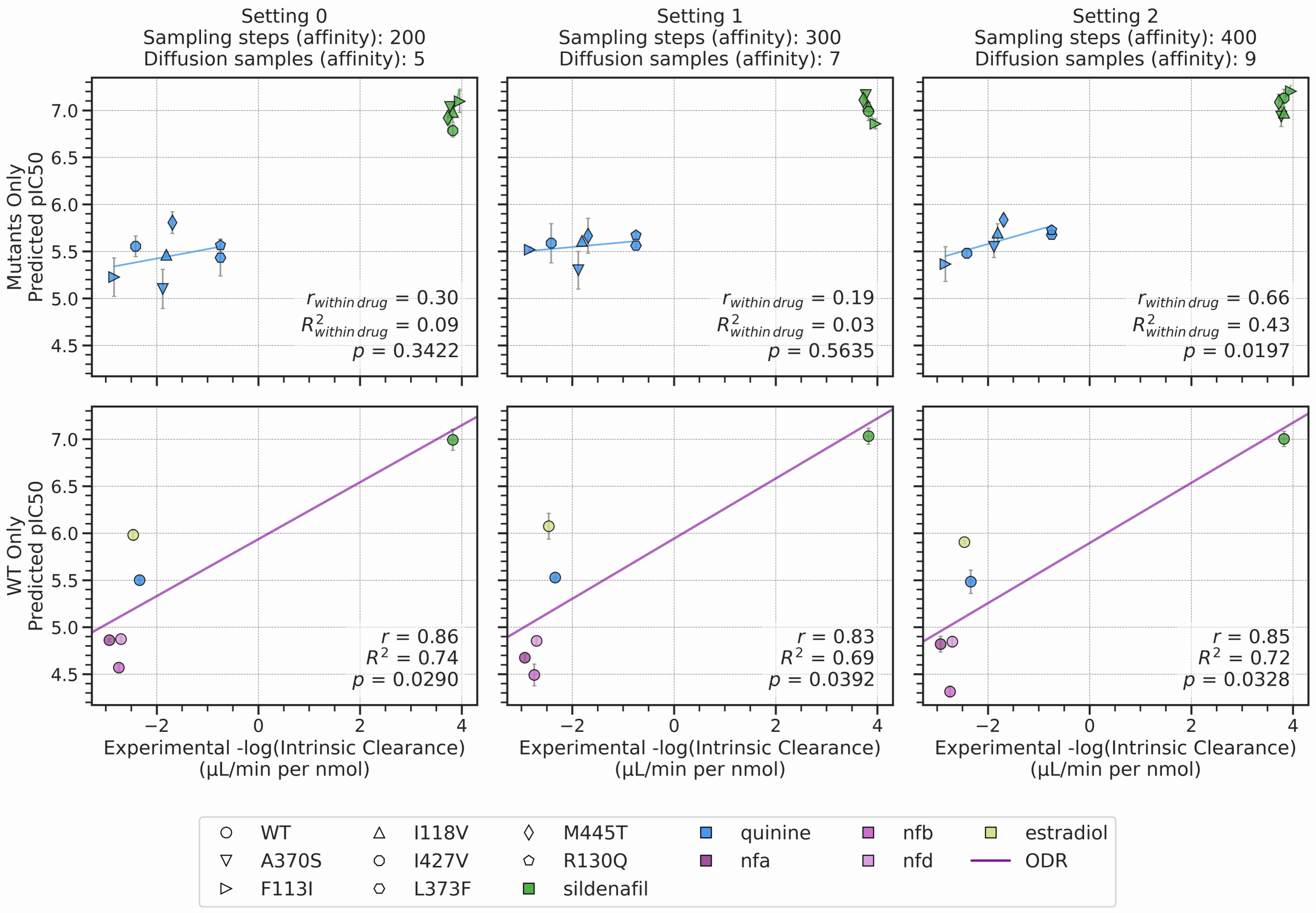

Figure S6. Boltz-2 prediction performance for CYP3A4 enzyme variants across multiple sampling settings. Scatter plots comparing predicted pIC_{50} values against experimental -log(intrinsic clearance) data measured in µl/min per nmol for CYP3A4 WT and mutant variants (A370S, F113I, I118V, I427V, L373F, M445T, R130Q) across three sampling parameter configurations. Each point represents a protein-substrate combination from a panel of 7 probe substrates representing different metabolic pathways and shows the mean predicted pIC_{50} value with error bars indicating the standard error of the mean. The straight lines represent orthogonal distance regression (ODR) fit. Pearson correlation coefficients (r) and coefficient of determination (R^2) are displayed for each variant-setting combination. For the mutant-only panels, agreement is summarized using within-drug metrics ($r_{within\ drug}$ and $R^2_{within\ drug}$), which quantify how well predicted values track differences across variants while controlling for systematic differences between substrates. For the WT-only panels, Pearson r and R^2 are reported directly. Because intrinsic clearance is plotted on a negative log scale, higher values correspond to lower metabolic turnover, resulting in positive correlations with predicted pIC_{50}. In this representation, compounds predicted to bind more tightly (higher pIC_{50}) are associated with reduced clearance, reflecting slower metabolic breakdown.

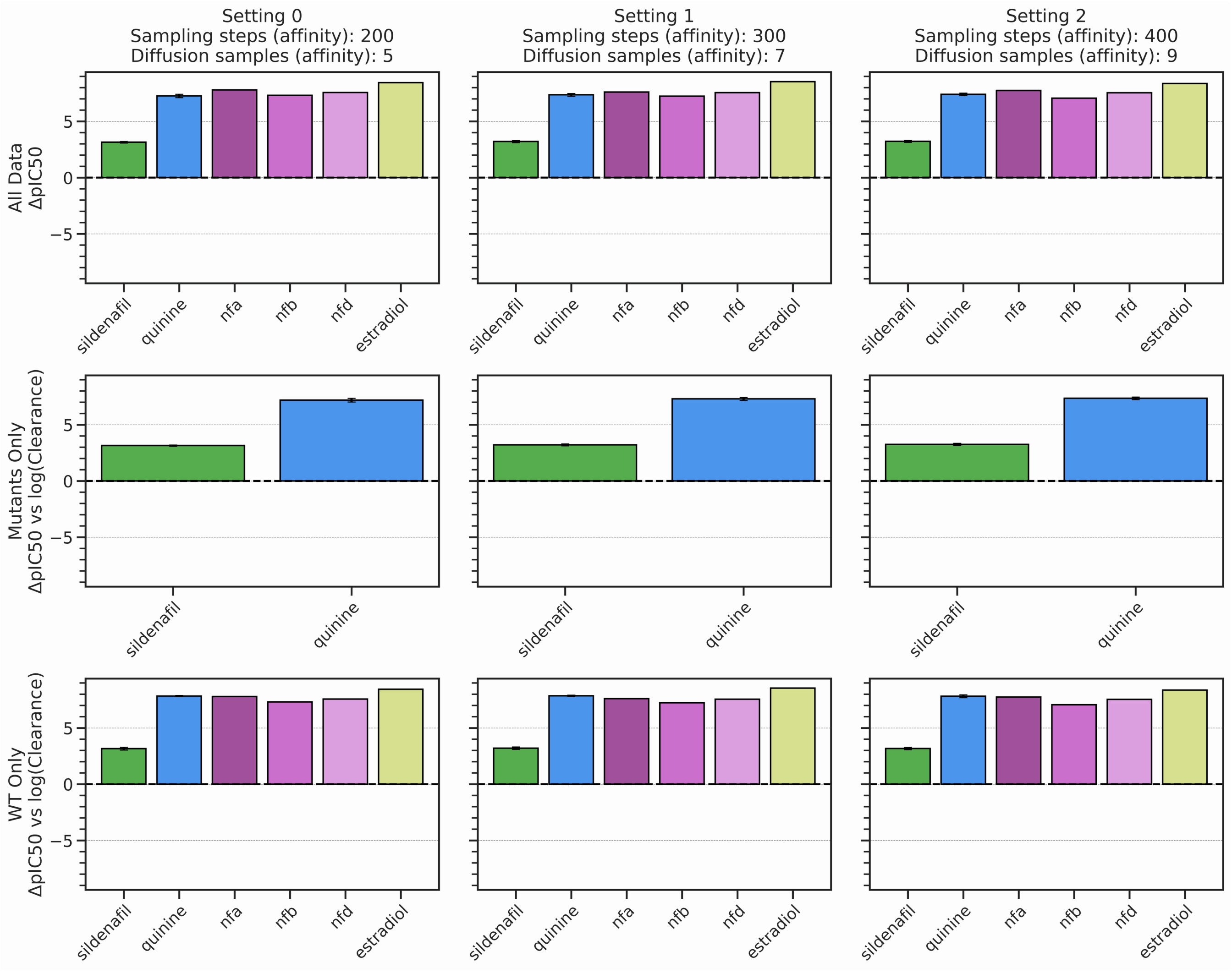

Figure S7. Substrate-specific prediction errors for CYP3A4 variants across sampling conditions. Bar plots show prediction errors for individual substrates across all CYP3A4 protein variants and three sampling settings, with drugs ordered from highest to lowest molecular weight. Bars represent mean ΔpIC_{50} values, with error bars indicating the standard error of the mean. Note that experimental clearance is measured in µl/min per nmol, with the logarithmic transformation used for comparison with predicted pIC_{50} values. The top panel displays errors aggregated across all protein variants for each substrate, the middle panel shows errors for mutant variants only, and the bottom panel presents errors for WT CYP3A4 only. Error bars represent standard error across variants (top and middle panels) or technical replicates (bottom panel). Across substrates, prediction errors are generally consistent, with no single compound dominating the deviation across all conditions.

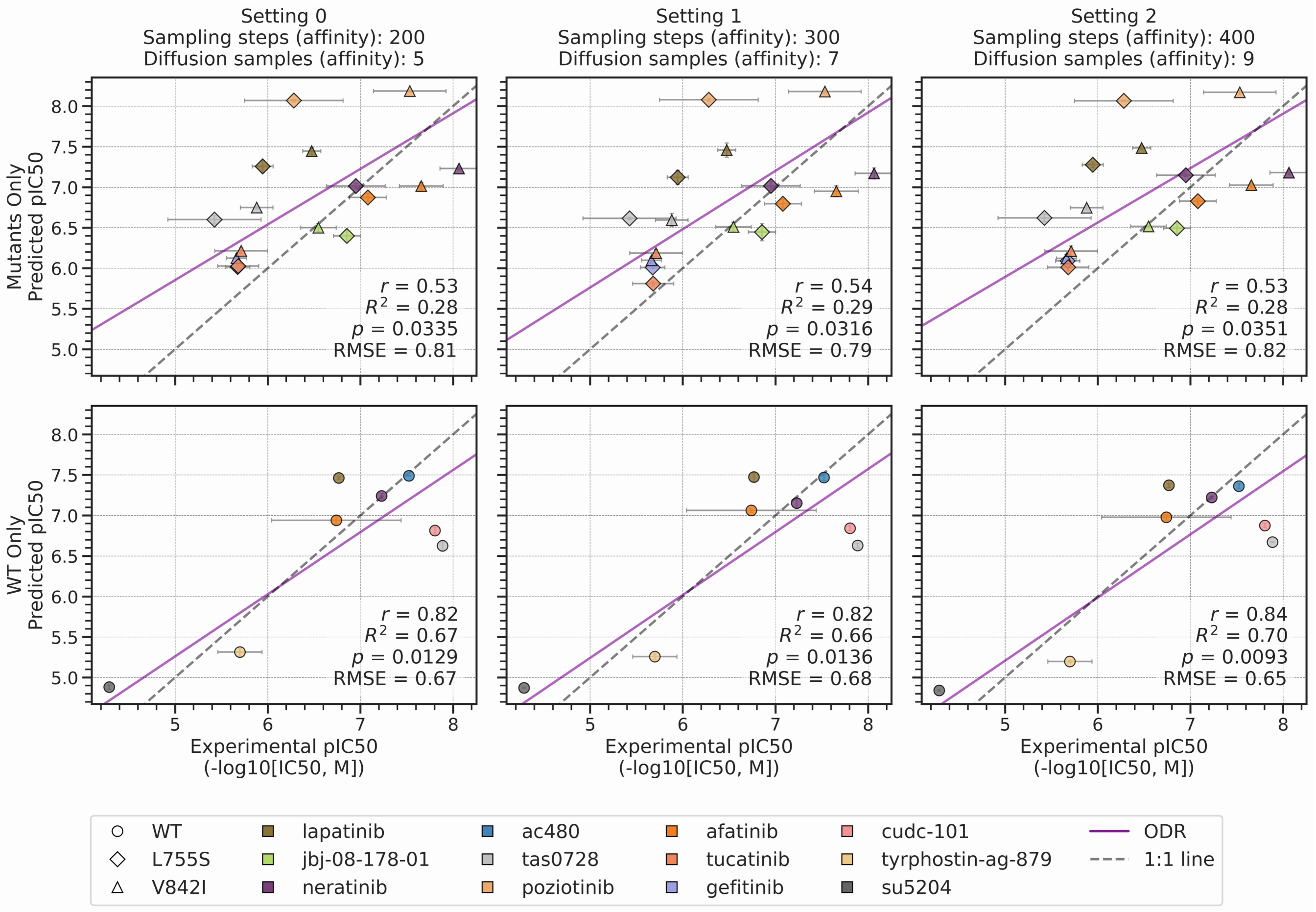

Figure S8. Boltz-2 prediction performance for HER2 kinase domain variants across multiple sampling settings. Scatter plots comparing predicted pIC_{50} values against experimental pIC_{50} data for HER2 kinase domain WT and two mutant variants (L755S, V842I) across three sampling parameter configurations. Each point represents a protein-drug combination from a panel of 12 kinase inhibitors including FDA-approved therapeutics and experimental compounds and shows the mean predicted pIC_{50} value with error bars indicating the standard error of the mean. The purple line represents orthogonal distance regression (ODR) fit, while the dashed gray line indicates perfect correlation (1:1). Pearson correlation coefficients (r), coefficient of determination (R^2), and root mean square error (RMSE) are displayed for each variant-setting combination. WT HER2 kinase domain demonstrates strong correlations (r = 0.82-0.84) with low RMSE values (0.66-0.70), representing excellent performance among all evaluated targets. Mutant variants show moderate but statistically significant correlations (r = 0.53–0.54, R^2 = 0.28–0.29, p ≈ 0.03) with higher RMSE values (0.79–0.82), reflecting reduced accuracy but meaningful capture of variant-specific effects. The consistent performance across sampling settings suggests robust structure-activity relationships for kinase inhibitor binding despite modeling only the isolated kinase domain.

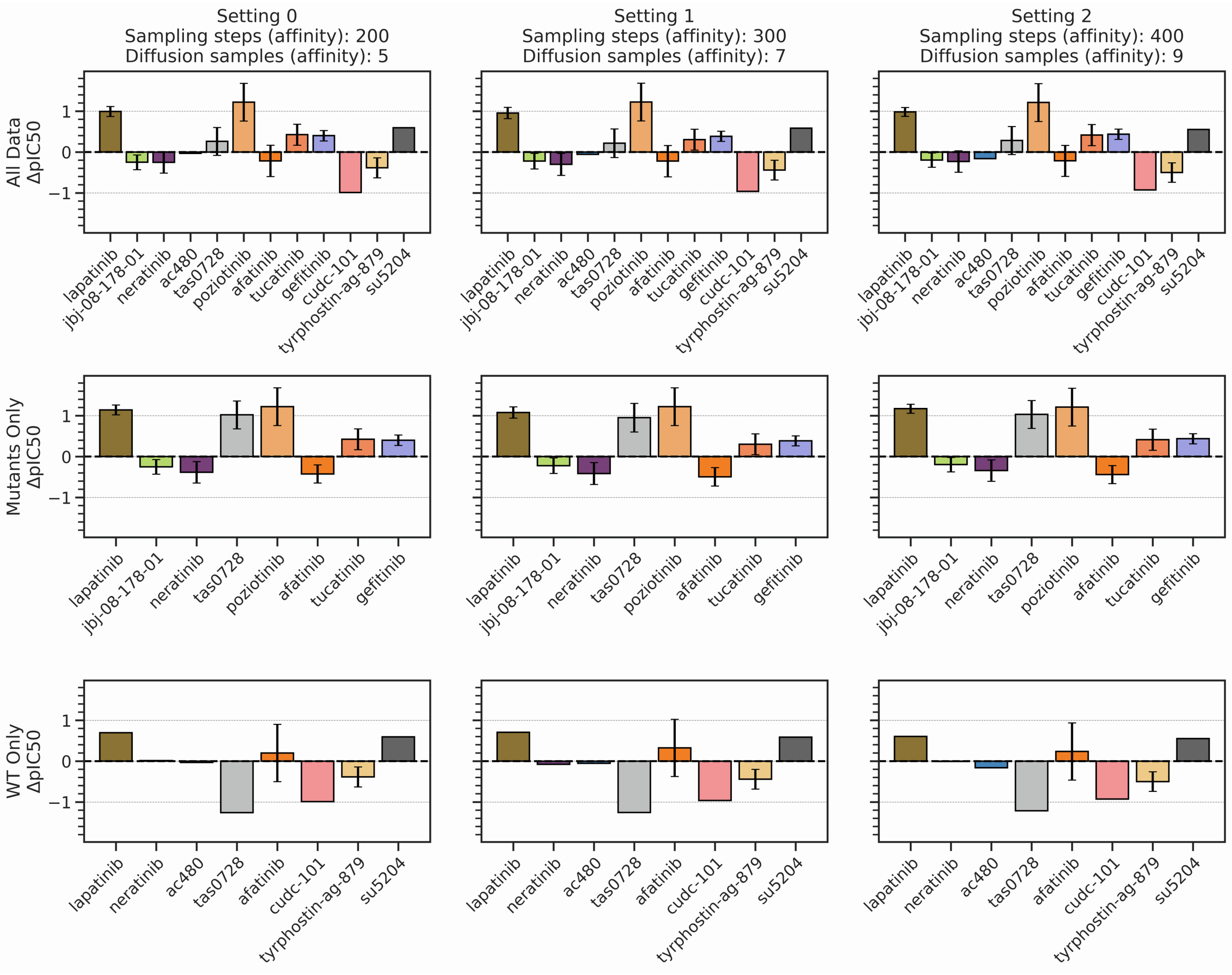

Figure S9. Drug-specific prediction errors for HER2 kinase domain variants across sampling conditions. Bar plots showing prediction errors (ΔpIC_{50} = predicted - experimental) for individual kinase inhibitors across all HER2 protein variants and three sampling settings, with drugs ordered from highest to lowest molecular weight. Bars represent mean ΔpIC_{50} values, with error bars indicating the standard error of the mean. The top panel displays errors aggregated across all protein variants for each drug, the middle panel shows errors for mutant variants only, and the bottom panel presents errors for WT HER2 kinase domain only. Error bars represent standard error across variants (top and middle panels) or technical replicates (bottom panel). The analysis reveals compound-specific prediction patterns, with first-generation inhibitors like lapatinib and gefitinib showing relatively consistent predictions across variants. The covalent inhibitor afatinib demonstrates underestimation across all conditions, potentially reflecting modeling limitations for covalent bond formation. Compounds like poziotinib and tyrphostin-ag-879 show larger deviations, while newer agents like neratinib and tucatinib exhibit variable performance across mutant backgrounds. The consistent error patterns across sampling settings indicate reproducible drug-specific challenges rather than parameter-dependent artifacts.

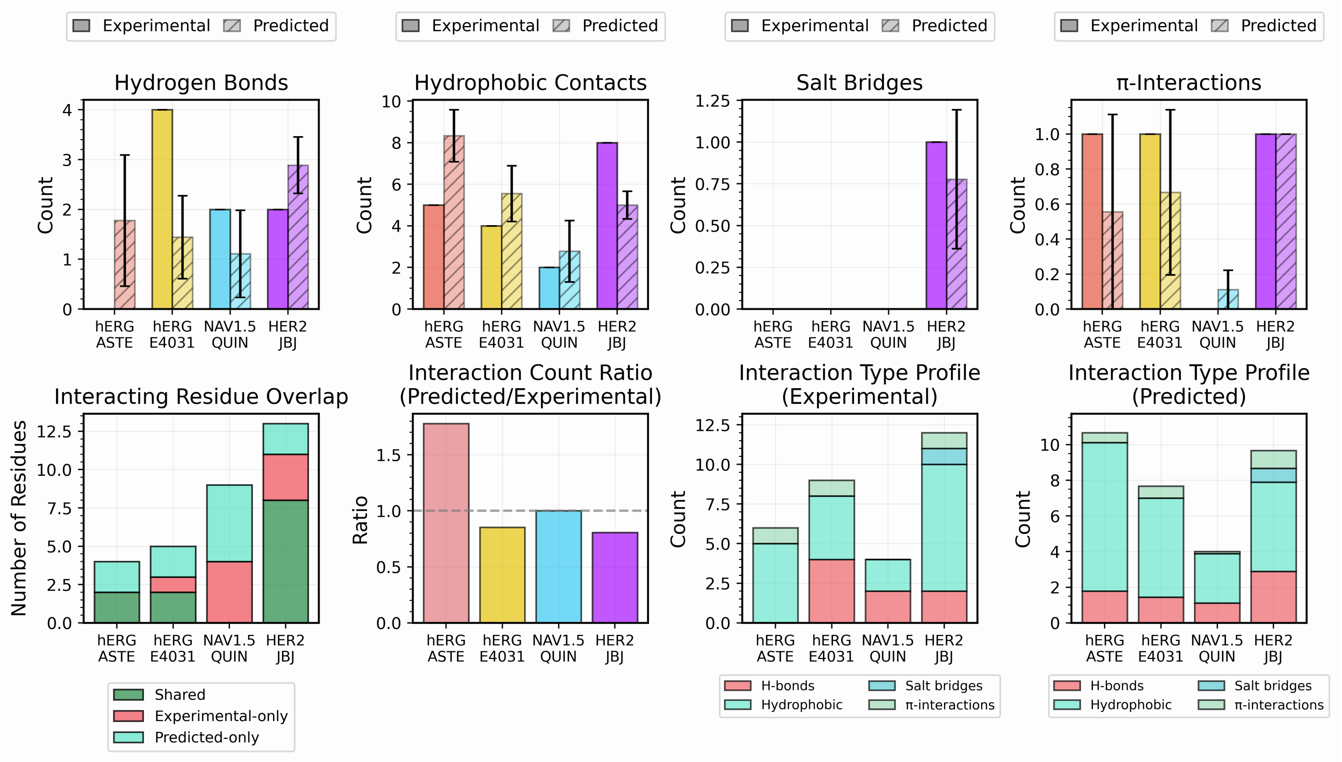

Figure S10. Molecular interaction analysis between predicted and experimental protein-drug complexes. Comparison of specific molecular interactions for representative protein-drug pairs: hERG with astemizole (ASTE) and E4031, $Na_V1.5$ with quinidine (QUIN), and HER2 with JBJ-08-178-01 (JBJ). (Top panels) Counts of hydrogen bonds, hydrophobic contacts, salt bridges, and π-interactions in experimental (solid bars) and predicted (hatched bars) structures. Predicted interaction counts are shown as mean values across three replicate predictions, with error bars indicating the standard error of the mean. (Bottom left) Interacting residue overlap showing shared residues (green), experimental-only contacts (red), and predicted-only contacts (blue). (Bottom center) Interaction count ratio (predicted/experimental) with dashed line indicating perfect agreement. (Bottom right) Interaction type profiles showing the distribution of contact types for experimental and predicted structures. Error bars represent standard deviation across multiple binding poses or structural conformations. The analysis reveals target-specific patterns in interaction reproduction, with some complexes showing excellent agreement in contact patterns while others exhibit differences in the detailed molecular recognition profiles.

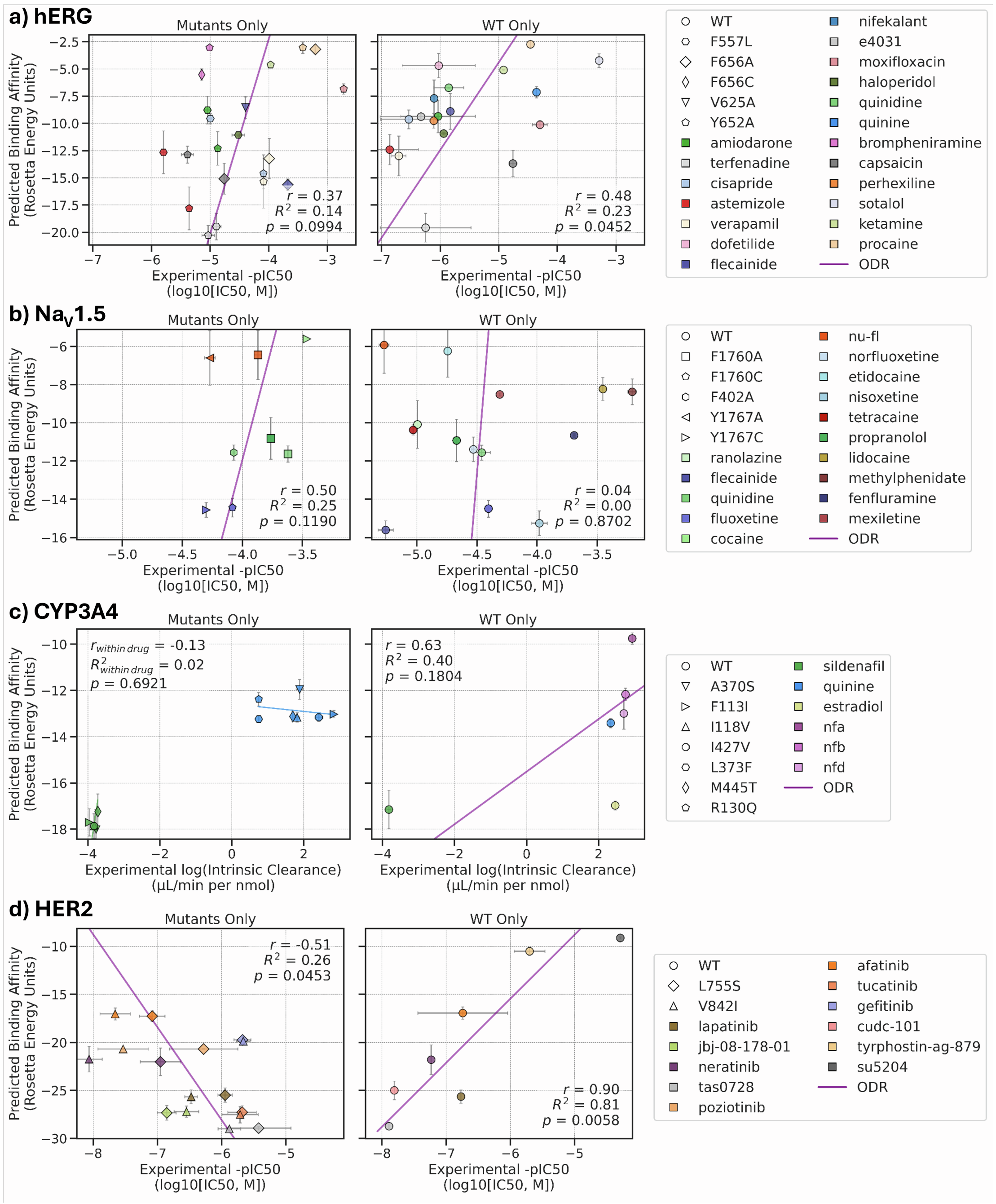

Figure S11. GALigandDock performance across targets and mutations. Scatter plots compare predicted binding scores from GALigandDock with experimental affinity measurements across hERG, NaV1.5, CYP3A4, and HER2 for both WT and mutant variants. Each point represents a protein–drug pair evaluated, using the same experimental datasets used for Boltz-2 benchmarking and shows the mean predicted pIC_{50} value, with error bars indicating the standard error of the mean. The purple line indicates the orthogonal distance regression (ODR) fit. Pearson correlation coefficients (r) and coefficients of determination (R^2) summarize agreement between predicted and experimental values. GALigandDock uses a genetic algorithm to sample ligand poses within the binding pocket and refines candidates using Rosetta-based scoring while allowing limited side-chain flexibility.

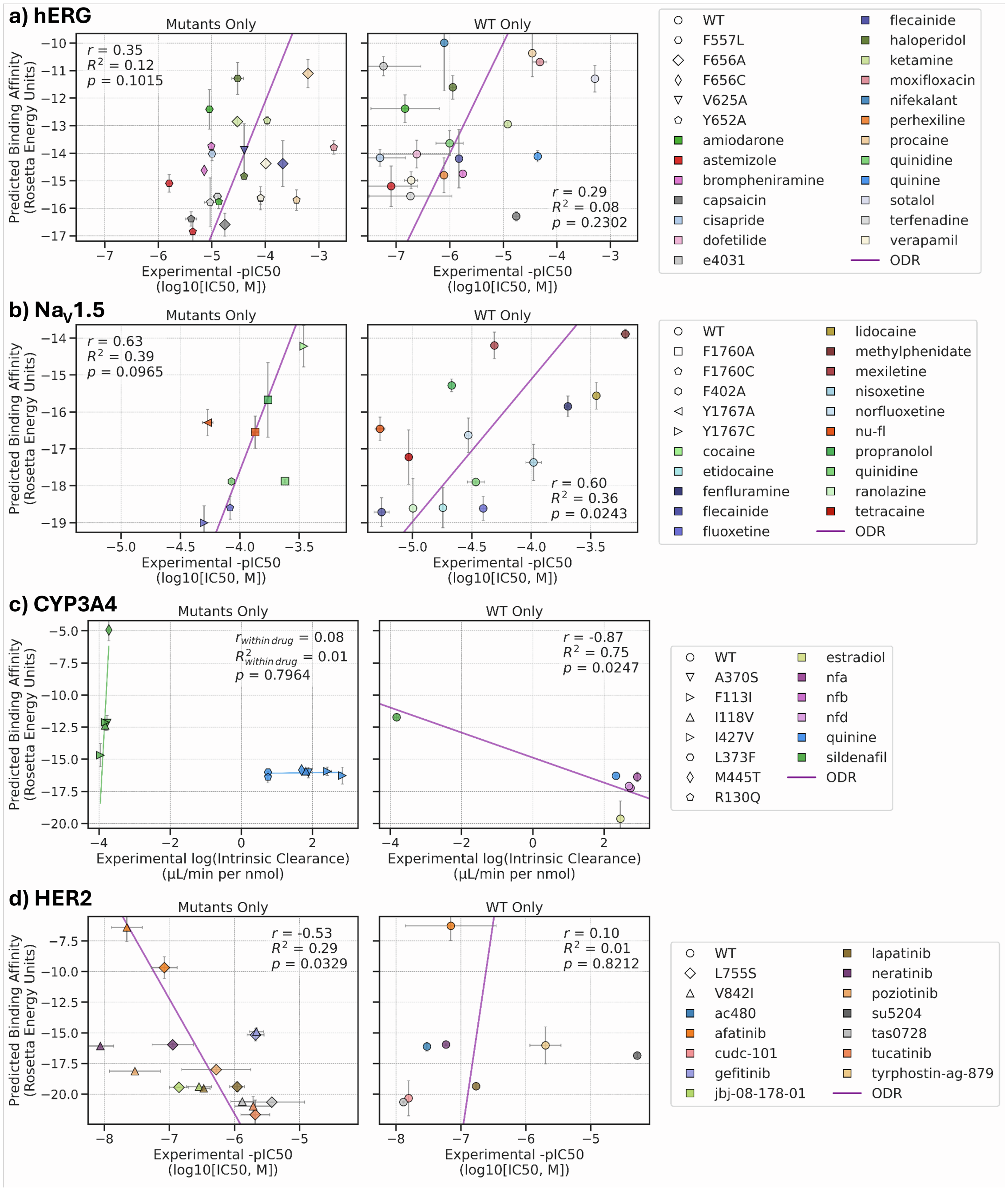

Figure S12. RosettaLigand with receptor flexibility performance across targets and mutations. Scatter plots compare predicted binding scores from RosettaLigand with flexible receptor sampling against experimental affinity measurements across hERG, Nav1.5, CYP3A4, and HER2. Each point represents a protein–drug pair evaluated, using the same datasets used for Boltz-2 benchmarking and shows the mean predicted pIC_{50} value, with error bars indicating the standard error of the mean. The purple line shows the orthogonal distance regression (ODR) fit. Pearson correlation coefficients (r) and R² values quantify agreement between predicted and experimental measurements. RosettaLigand allows side-chain repacking and backbone motion near the ligand during docking to account for local receptor flexibility.

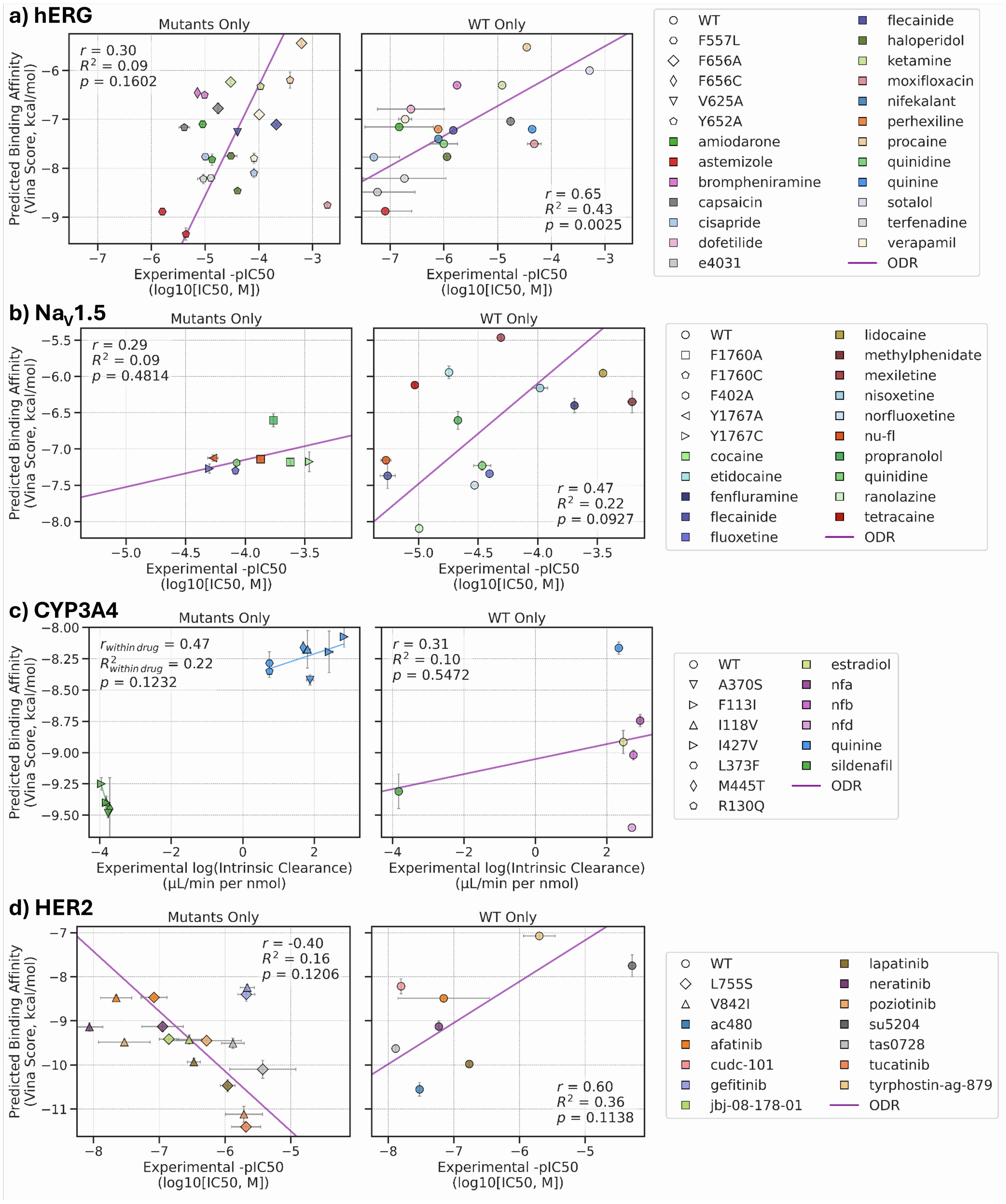

Figure S13. AutoDock Vina performance across targets and mutations. Scatter plots compare predicted binding scores from AutoDock Vina with experimental affinity measurements across hERG, NaV1.5, CYP3A4, and HER2. Each point represents a protein–drug pair evaluated using the same experimental datasets used for Boltz-2 benchmarking and shows the mean predicted pIC_{50} value, with error bars indicating the standard error of the mean. The purple line indicates the orthogonal distance regression (ODR) fit. Pearson correlation coefficients (r) and R^2 values summarize agreement between predicted and experimental measurements. AutoDock Vina uses a semi-empirical scoring function and searches ligand poses within a largely fixed receptor pocket with limited receptor flexibility.

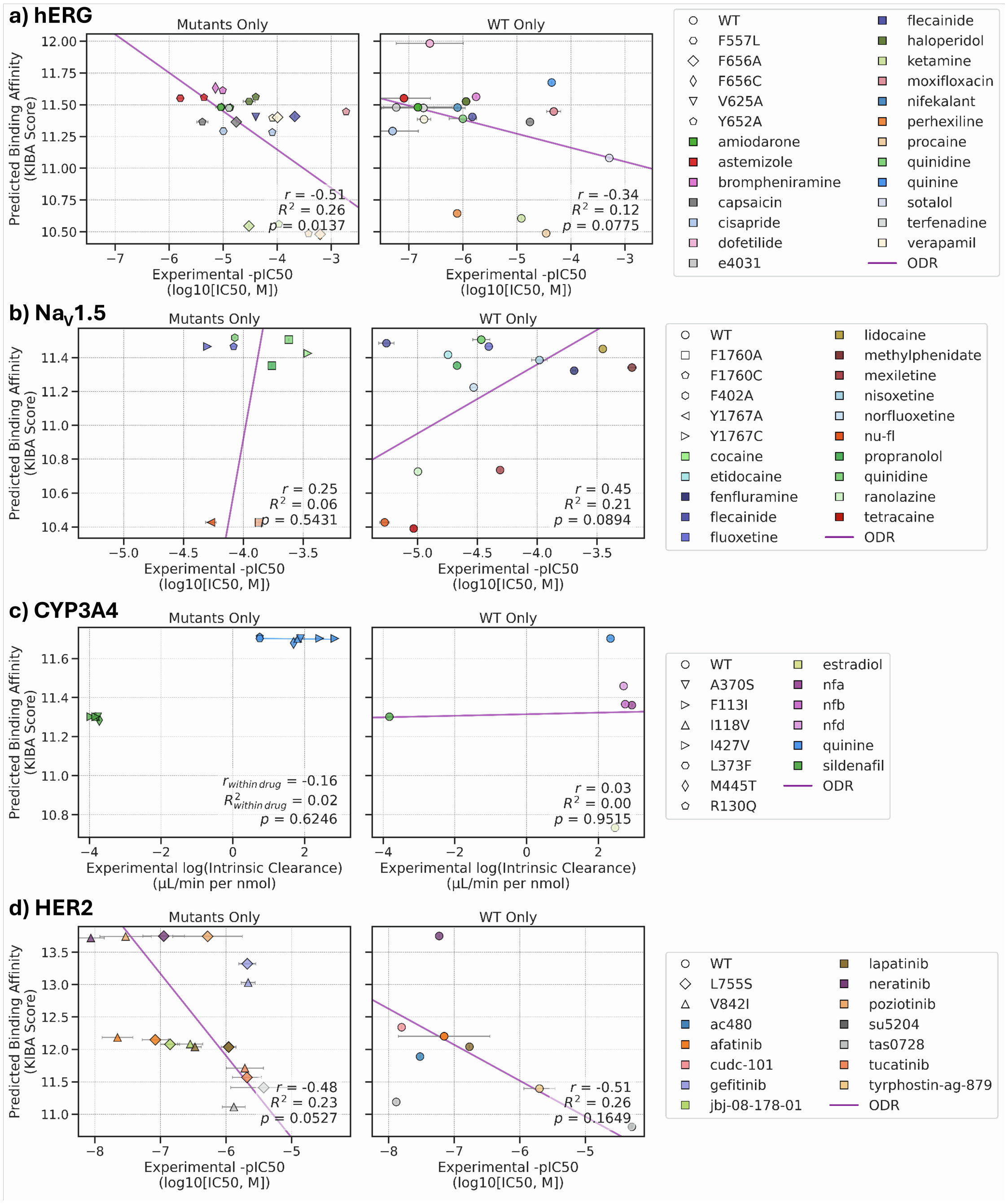

Figure S14. DeepDTA performance across targets and mutations. Scatter plots compare predicted binding affinity values from the sequence-based deep learning model DeepDTA with experimental affinity measurements across hERG, $Na_V1.5$, CYP3A4, and HER2. Each point represents a protein–drug pair evaluated using identical experimental datasets and shows the mean predicted pIC_{50} value, with error bars indicating the standard error of the mean. The purple line indicates the orthogonal distance regression (ODR) fit. Pearson correlation coefficients (r) and R^2 values summarize agreement between predicted and experimental measurements. DeepDTA predicts binding affinity directly from drug SMILES strings and protein amino-acid sequences without explicitly modeling three-dimensional structure.

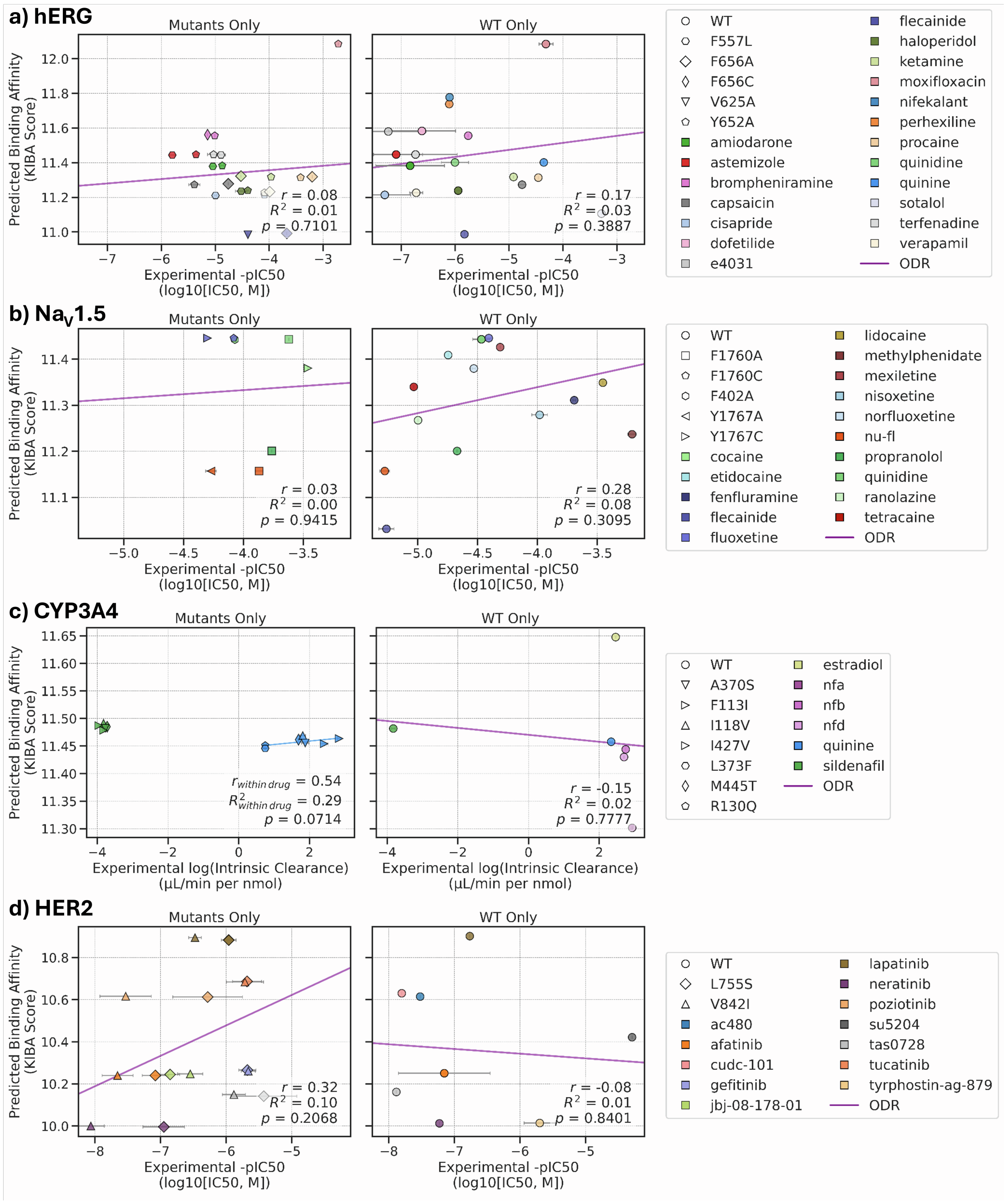

Figure S15. GraphDTA performance across targets and mutations. Scatter plots compare predicted binding affinity values from the GraphDTA model with experimental affinity measurements across hERG, $Na_V1.5$, CYP3A4, and HER2. Each point represents a protein–drug pair evaluated using identical experimental datasets and shows the mean predicted pIC_{50} value, with error bars indicating the standard error of the mean. The purple line indicates the orthogonal distance regression (ODR) fit. Pearson correlation coefficients (|r|) and R² values summarize agreement between predicted and experimental measurements. GraphDTA uses graph neural networks to encode ligand molecular graphs together with protein sequence features to predict drug–target affinity without explicit structural docking.

Supplementary Table 1. IC_{50} measurements for hERG variant–drug combinations obtained from experimental studies

Variant	Drug	IC_{50} (μM)	Error (μM)	Source
**WT**	Amiodarone	2.7	0.3	1
**WT**	Amiodarone	0.0259		2
**WT**	Amiodarone	0.045		4
**WT**	Astemizole	0.25	0.02	1
**WT**	Astemizole	0.026		2
**WT**	Brompheniramine	1.75		7
**WT**	Capsaicin	17.45	2.63	8
**WT**	Cisapride	1.1	0.1	1
**WT**	Cisapride	0.018		3
**WT**	Cisapride	0.0069		2
**WT**	Cisapride	0.0445		9
**WT**	Dofetilide	2.5	0.2	1
**WT**	Dofetilide	0.32		12
**WT**	Dofetilide	0.0179	0.0012	14
**WT**	E4031	0.0181		2
**WT**	E4031	0.0077		16
**WT**	E4031	1.39	0.19	17
**WT**	Flecainide	1.49		18
**WT**	Haloperidol	1.3	0.1	1
**WT**	Haloperidol	1		19
**WT**	Ketamine	12.1		10
**WT**	Moxifloxacin	65	4.2	11
**WT**	Moxifloxacin	35.7		20
**WT**	Nifekalant	0.79		21
**WT**	Perhexiline	0.78		22
**WT**	Procaine	34.8		13
**WT**	Quinidine	0.8	0.1	23
**WT**	Quinidine	0.41		24
**WT**	Quinidine	3	0.03	23
**WT**	Quinine	44	0.6	23
**WT**	Sotalol	515.5		25
WT	Terfenadine	1.1	0.04	1
WT	Terfenadine	0.031		3
WT	Verapamil	0.143		5
WT	Verapamil	0.252		6
Y652A	Amiodarone	13.5	1.05	1
Y652A	Astemizole	4.4	0.3	1
Y652A	Brompheniramine	9.8		7
Y652A	Capsaicin	4.11	0.96	8
Y652A	Cisapride	81.4	8.1	1
Y652A	Haloperidol	40.1	1.9	1
Y652A	Ketamine	108		10
Y652A	Moxifloxacin	1900		11
Y652A	Procaine	382		13
Y652A	Terfenadine	9.3	2.3	1
Y652A	Verapamil	81.6		15
F557L	Amiodarone	9.02	1.05	1
F557L	Astemizole	1.6	0.2	1
F557L	Cisapride	10.1	0.6	1
F557L	Haloperidol	30.3	7.5	1
F557L	Terfenadine	12.8	2.3	1
F656A	Capsaicin	17.45		8
F656A	Flecainide	212.18		18
F656A	Ketamine	30		10
F656A	Procaine	626		13
F656A	Verapamil	102		15
F656C	Brompheniramine	7.2		7

Supplementary Table 2. IC_{50} measurements for $Na_V1.5$ variant–drug combinations obtained from experimental studies

Variant	Drug	IC_{50} (μM)	Error (μM)	Source
**WT**	Etidocaine	18		[26]
**WT**	Fenfluramine	203.5	5.5	[27]
**WT**	Flecainide	5.5	0.8	[28]
**WT**	Fluoxetine	39.4	2	[27]
**WT**	Lidocaine	353		[29]
**WT**	Methylphenidate	618.7	51.5	[27]
**WT**	Mexiletine	49		[30]
**WT**	Nisoxetine	104.5	15.6	[27]
**WT**	Norfluoxetine	29.5	1	[27]
**WT**	NU-FL (flecainide analogue)	5.32	0.51	[28]
**WT**	Propranolol	21.4		[31]
**WT**	Quinidine	28.9	2.2	[28]
**WT**	Quinidine	40.3	1.1	[32]
**WT**	Ranolazine	10.1		[33]
**WT**	Tetracaine	9.3		[34]
**Y1767A**	NU-FL (flecainide analogue)	53.7	5.55	[28]
**Y1767C**	Cocaine	345		[35]
**Y1767C**	Fluoxetine	50.1	1.8	[27]
**F1760A**	NU-FL (flecainide analogue)	134.9	9.96	[28]
**F1760A**	Propranolol	172.7		[36]
**F1760A**	Quinidine	239.3	1.2	[32]
**F1760C**	Fluoxetine	82.8	3.2	[27]
**F402A**	Quinidine	84.8	1.3	[32]

Supplementary Table 3. Intrinsic clearance measurements for Cytochrome P450 3A4 (CYP3A4) variant–drug combinations obtained from experimental studies

Variant	Drug	Intrinsic Clearance (μl/min per nmol)	Error (μl/min per nmol)	Source
**WT**	quinine	217.5	20.6	37
**M445T**	quinine	49.2	0.8	37
**I118V**	quinine	64.8	11.4	37
**R130Q**	quinine	5.6	0	37
**L373F**	quinine	5.6	0.1	37
**F113I**	quinine	688.5	73.4	37
**A370S**	quinine	76.2	17.3	37
**I427V**	quinine	260	64.1	37
**WT**	estradiol	291		38
**WT**	NFa	857		39
**WT**	NFb	558		39
**WT**	NFd	504		39
**WT**	sildenafil	0.00015	0.00002	40
**M445T**	sildenafil	0.00019	0.00001	40
**I118V**	sildenafil	0.00015	0	40
**F113I**	sildenafil	0.00011	0	40
**A370S**	sildenafil	0.00017	0.00001	40
**I427V**	sildenafil	0.00015	0.00001	40

Supplementary Table 4. IC_{50} measurements for HER2 variant–drug combinations obtained from experimental studies

Variant	Drug	IC_{50} (nM)	Error (nM)	Source
**WT**	Lapatinib	171		41
**WT**	Afatinib	350		42
**WT**	Afatinib	14		43
**WT**	Neratinib	59		44
**WT**	CUDC-101	15.7		45
**WT**	AC480 (BMS-599626)	30		46
**WT**	Tyrphostin-AG-879	2000	1000	47
**WT**	SU5204	51500		48
**WT**	TAS0728	13		49
**L755S**	Lapatinib	1424		41
**L755S**	JBJ-08-178-01	139.92	45.28	50
**L755S**	Afatinib	83.17	35.68	50
**L755S**	Poziotinib	523.45	440.32	50
**L755S**	Neratinib	112	69.73	50
**L755S**	Tucatinib	2085.3	980.62	50
**L755S**	Lapatinib	844.97	380.92	50
**L755S**	Gefitinib	2112.33	618.9	50
**L755S**	TAS0728	3757	3081.57	50
**V842I**	JBJ-08-178-01	284.03	117.62	50
**V842I**	Afatinib	22.06	10.94	50
**V842I**	Poziotinib	29.34	42.95	50
**V842I**	Neratinib	8.64	3.81	50
**V842I**	Tucatinib	1940.3	1114.6	50
**V842I**	Lapatinib	335.63	74.45	50
**V842I**	Gefitinib	2177	527.45	50
**V842I**	TAS0728	1316.4	505.73	50

Data S1. Amino acid sequences for WT proteins

A colon (“:”) indicates the start of a new chain within the protein.

>hERG2

WTILHYSPFKAVWDWLILLLVIYTAVFTPYSAAFLLKETEEGPPATECGYACQPLAVVDLIVDIMFIVDILINFRTTYVNANEEVVSHPGRIAVH
YFKGWFLIDMVAAIPFDLLIFGSGSEELIGLLKTARLLRLVRVARKLDRYSEYGAAVLFLLMCTFALIAHWLACIWYAIGNMEQPHMDSRIGW
LHNLGDQIGKPYNSSGLGGPSIKDKYVTALYFTFSSLTSVGFGNVSPNTNSEKIFSICVMLIGSLMYASIFGNVSAIIQRL:WTILHYSPFKAVW
DWLILLLVIYTAVFTPYSAAFLLKETEEGPPATECGYACQPLAVVDLIVDIMFIVDILINFRTTYVNANEEVVSHPGRIAVHYFKGWFLIDMVAAI
PFDLLIFGSGSEELIGLLKTARLLRLVRVARKLDRYSEYGAAVLFLLMCTFALIAHWLACIWYAIGNMEQPHMDSRIGWLHNLGDQIGKPYNS
SGLGGPSIKDKYVTALYFTFSSLTSVGFGNVSPNTNSEKIFSICVMLIGSLMYASIFGNVSAIIQRL:WTILHYSPFKAVWDWLILLLVIYTAVFT
PYSAAFLLKETEEGPPATECGYACQPLAVVDLIVDIMFIVDILINFRTTYVNANEEVVSHPGRIAVHYFKGWFLIDMVAAIPFDLLIFGSGSEELI
GLLKTARLLRLVRVARKLDRYSEYGAAVLFLLMCTFALIAHWLACIWYAIGNMEQPHMDSRIGWLHNLGDQIGKPYNSSGLGGPSIKDKYV
TALYFTFSSLTSVGFGNVSPNTNSEKIFSICVMLIGSLMYASIFGNVSAIIQRL:WTILHYSPFKAVWDWLILLLVIYTAVFTPYSAAFLLKETEE
GPPATECGYACQPLAVVDLIVDIMFIVDILINFRTTYVNANEEVVSHPGRIAVHYFKGWFLIDMVAAIPFDLLIFGSGSEELIGLLKTARLLRLVR
VARKLDRYSEYGAAVLFLLMCTFALIAHWLACIWYAIGNMEQPHMDSRIGWLHNLGDQIGKPYNSSGLGGPSIKDKYVTALYFTFSSLTSVG
FGNVSPNTNSEKIFSICVMLIGSLMYASIFGNVSAIIQRL

>Na$_V$1.5

PIRRAAVKILVHSLFNMLIMCTILTNCVFMAQHDPPPWTKYVEYTFTAIYTFESLVKILARGFCLHAFTFLRDPWNWLDFSVIIMAYTTEFVDL
GNVSALRTFRVLRALKTISVISGLKTIVGALIQSVKKLADVMVLTVFCLSVFALIGLQLFMGNLRHKCVRNFTALNGTNGSVEADGLVWESLD
LYLSDPENYLLKNGTSDVLLCGNSSDAGTCPEGYRCLKAGENPDHGYTSFDSFAWAFLALFRLMTQDCWERLYQQTLRSAGKIYMIFFML
VIFLGSFYLVNLILAVVAMAYEEQNQATIAETEE:CCPLWMSIKQGVKLVVMDPFTDLTITMCIVLNTLFMALEHYNMTSEFEEMLQVGNLVF
TGIFTAEMTFKIIALDPYYYFQQGWNIFDSIIVILSLMELGLSRMSNLSVLRSFRLLRVFKLAKSWPTLNTLIKIIGNSVGALGNLTLVLAIIVFIF
AVVGMQLFGKNYSELRDSDSGLLPRWHMMDFFHAFLIIFRILCGEWIETMWDCMEVSGQSLCLLVFLLVMVIGNLVVLNLFLALLLSSFSA:
GKVWWRLRKTCYHIVEHSWFETFIIFMILLSSGALAFEDIYLEERKTIKVLLEYADKMFTYVFVLEMLLKWVAYGFKKYFTNAWCWLDFLIVD
VSLVSLVANTLGFAEMGPIKSLRTLRALRPLRALSRFEGMRVVVNALVGAIPSIMNVLLVCLIFWLIFSIMGVNLFAGKFGRCINQTEGDLPLN
YTIVNNKSQCESLNLTGELYWTKVKVNFDNVGAGYLALLQVATFKGWMDIMYAAVDSRGYEEQPQWEYNLYMYIYFVIFIIFGSFFTLNLFIG
VIIDNFNQQKKKLGGQDIFMTEEQKKYYNAMKKLGSKKPQKPIPRPLNKYQGFIFDIVTKQAFDVTIMFLICLNMVTMMVETDDQSPEKINI
LAKINLLFVAIFTGECIVKLAALRHYYFTNSWNIFDFVVVILSIVGTVLSDIIQKYFFSPTLFRVIRLARIGRILRLIRGAKGIRTLLFALMMSLPALF
NIGLLLFLVMFIYSIFGMANFAYVKWEAGIDDMFNFQTFANSMLCLFQITTSAGWDGLLSPILNTGPPYCDPTLPNSNGSRGDCGSPAVGIL
FFTTYIIISFLIVVNMYIAIILENFSVATEE

>CYP3A4

KLGIPGPTPLPFLGNILSYHKGFCMFDMECHKKYGKVWGFYDGQQPVLAITDPDMIKTVLVKECYSVFTNRRPFGPVGFMKSAISIAEDEE
WKRLRSLLSPTFTSGKLKEMVPIIAQYGDVLVRNLRREAETGKPVTLKDVFGAYSMDVITSTSFGVNIDSLNNPQDPFVENTKKLLRFDFLD
PFFLSITVFPFLIPILEVLNICVFPREVTNFLRKSVKRMKESRLEDTQKHRVDFLQLMIDSQNSKETESHKALSDLELVAQSIIFIFAGYETTSS
VLSFIMYELATHPDVQQKLQEEIDAVLPNKAPPTYDTVLQMEYLDMVVNETLRLFPIAMRLERVCKKDVEINGMFIPKGVVVMIPSYALHRD
PKYWTEPEKFLPERFSKKNKDNIDPYIYTPFGSGPRNCIGMRFALMNMKLALIRVLQNFSFKPCKETQIPLKLSLGGLLQPEKPVVLKVESR
D

>HER2

AQMRILKETELRKVKVLGSGAFGTVYKGIWIPDGENVKIPVAIKVLRENTSPKANKEILDEAYVMAGVGSPYVSRLLGICLTSTVQLVTQLMP
YGCLLDHVRENRGRLGSQDLLNWCMQIAKGMSYLEDVRLVHRDLAARNVLVKSPNHVKITDFGLARLLDIDETEYHADGGKVPIKWMALE

SILRRRFTHQSDVWSYGVTVWELMTFGAKPYDGIPAREIPDLLEKGERLPQPPICTIDVYMIMVKCWMIDSECRPRFRELVSEFSRMARDPQ
RFVVIQN

References

1. Saxena, P., Zangerl-Plessl, E.-M., Linder, T., Windisch, A., Hohaus, A., Timin, E., Hering, S., and Stary-Weinzinger, A. (2016). New potential binding determinant for hERG channel inhibitors. Sci. Rep. *6*, 24182. https://doi.org/10.1038/srep24182.

2. Chiu, P.J.S., Marcoe, K.F., Bounds, S.E., Lin, C.-H., Feng, J.-J., Lin, A., Cheng, F.-C., Crumb, W.J., and Mitchell, R. (2004). Validation of a [3H]astemizole binding assay in HEK293 cells expressing HERG K+ channels. J. Pharmacol. Sci. *95*, 311–319. https://doi.org/10.1254/jphs.fpe0040101.

3. Orvos, P., Kohajda, Z., Szlovák, J., Gazdag, P., Árpádffy-Lovas, T., Tóth, D., Geramipour, A., Tálosi, L., Jost, N., Varró, A., et al. (2019). Evaluation of Possible Proarrhythmic Potency: Comparison of the Effect of Dofetilide, Cisapride, Sotalol, Terfenadine, and Verapamil on hERG and Native IKr Currents and on Cardiac Action Potential. Toxicol. Sci. Off. J. Soc. Toxicol. *168*, 365–380. https://doi.org/10.1093/toxsci/kfy299.

4. Zhang, Y., Colenso, C.K., El Harchi, A., Cheng, H., Witchel, H.J., Dempsey, C.E., and Hancox, J.C. (2016). Interactions between amiodarone and the hERG potassium channel pore determined with mutagenesis and in silico docking. Biochem. Pharmacol. *113*, 24–35. https://doi.org/10.1016/j.bcp.2016.05.013.

5. Zhang, S., Zhou, Z., Gong, Q., Makielski, J.C., and January, C.T. (1999). Mechanism of block and identification of the verapamil binding domain to HERG potassium channels. Circ. Res. *84*, 989–998. https://doi.org/10.1161/01.res.84.9.989.

6. Johnson, A.A., and Trudeau, M.C. (2024). Inhibition of hERG K channels by verapamil at physiological temperature: Implications for the CiPA initiative. J. Pharmacol. Toxicol. Methods *130*, 107562. https://doi.org/10.1016/j.vascn.2024.107562.

7. Park, S.-J., Kim, K.-S., and Kim, E.-J. (2008). Blockade of HERG K+ channel by an antihistamine drug brompheniramine requires the channel binding within the S6 residue Y652 and F656. J. Appl. Toxicol. JAT *28*, 104–111. https://doi.org/10.1002/jat.1252.

8. Xing, J., Ma, J., Zhang, P., and Fan, X. (2010). Block effect of capsaicin on hERG potassium currents is enhanced by S6 mutation at Y652. Eur. J. Pharmacol. *630*, 1–9. https://doi.org/10.1016/j.ejphar.2009.11.009.

9. Rampe, D., Roy, M.L., Dennis, A., and Brown, A.M. (1997). A mechanism for the proarrhythmic effects of cisapride (Propulsid): high affinity blockade of the human cardiac potassium channel HERG. FEBS Lett. *417*, 28–32. https://doi.org/10.1016/s0014-5793(97)01249-0.

10. Zhang, P., Xing, J., Luo, A., Feng, J., Liu, Z., Gao, C., and Ma, J. (2013). Blockade of the human ether-a-go-go-related gene potassium channel by ketamine. J. Pharm. Pharmacol. *65*, 1321–1328. https://doi.org/10.1111/jphp.12095.

11. Alexandrou, A.J., Duncan, R.S., Sullivan, A., Hancox, J.C., Leishman, D.J., Witchel, H.J., and Leaney, J.L. (2006). Mechanism of hERG K+ channel blockade by the fluoroquinolone antibiotic moxifloxacin. Br. J. Pharmacol. *147*, 905–916. https://doi.org/10.1038/sj.bjp.0706678.

12. Ficker, E., Jarolimek, W., Kiehn, J., Baumann, A., and Brown, A.M. (1998). Molecular Determinants of Dofetilide Block of HERG K+ Channels. Circ. Res. *82*, 386–395. https://doi.org/10.1161/01.RES.82.3.386.

13. Wang, N., Ma, J.H., and Zhang, P.H. (2013). Procaine, a state-dependent blocker, inhibits HERG channels by helix residue Y652 and F656 in the S6 transmembrane domain. J. Pharmacol. Sci. *123*, 25–35. https://doi.org/10.1254/jphs.13007fp.

14. Li, P., Sun, H., Zhou, P., Ma, C., Hu, G., Jiang, H., Li, M., Liu, H., and Gao, Z. (2012). Comparison of the effects of DC031050, a class III antiarrhythmic agent, on hERG channel and three neuronal potassium channels. Acta Pharmacol. Sin. *33*, 728–736. https://doi.org/10.1038/aps.2012.41.

15. Duan, J., Ma, J., Zhang, P., Wang, X., Zou, A., and Tu, D. (2007). Verapamil blocks HERG channel by the helix residue Y652 and F656 in the S6 transmembrane domain. Acta Pharmacol. Sin. *28*, 959–967. https://doi.org/10.1111/j.1745-7254.2007.00562.x.

16. Zhou, Z., Gong, Q., Ye, B., Fan, Z., Makielski, J.C., Robertson, G.A., and January, C.T. (1998). Properties of HERG channels stably expressed in HEK 293 cells studied at physiological temperature. Biophys. J. *74*, 230–241. https://doi.org/10.1016/S0006-3495(98)77782-3.

17. Ishii, K., Nagai, M., Takahashi, M., and Endoh, M. (2003). Dissociation of E-4031 from the HERG channel caused by mutations of an amino acid results in greater block at high stimulation frequency. Cardiovasc. Res. *57*, 651–659. https://doi.org/10.1016/s0008-6363(02)00774-5.

18. Melgari, D., Zhang, Y., El Harchi, A., Dempsey, C.E., and Hancox, J.C. (2015). Molecular basis of hERG potassium channel blockade by the class Ic antiarrhythmic flecainide. J. Mol. Cell. Cardiol. *86*, 42–53. https://doi.org/10.1016/j.yjmcc.2015.06.021.

19. Suessbrich, H., Schönherr, R., Heinemann, S.H., Attali, B., Lang, F., and Busch, A.E. (1997). The inhibitory effect of the antipsychotic drug haloperidol on HERG potassium channels expressed in Xenopus oocytes. Br. J. Pharmacol. *120*, 968–974. https://doi.org/10.1038/sj.bjp.0700989.

20. Chen, X., Cass, J.D., Bradley, J.A., Dahm, C.M., Sun, Z., Kadyszewski, E., Engwall, M.J., and Zhou, J. (2005). QT prolongation and proarrhythmia by moxifloxacin: concordance of preclinical models in relation to clinical outcome. Br. J. Pharmacol. *146*, 792–799. https://doi.org/10.1038/sj.bjp.0706389.

21. Kushida, S., Ogura, T., Komuro, I., and Nakaya, H. (2002). Inhibitory effect of the class III antiarrhythmic drug nifekalant on HERG channels: mode of action. Eur. J. Pharmacol. *457*, 19–27. https://doi.org/10.1016/s0014-2999(02)02666-3.

22. Walker, B.D., Valenzuela, S.M., Singleton, C.B., Tie, H., Bursill, J.A., Wyse, K.R., Qiu, M.R., Breit, S.N., and Campbell, T.J. (1999). Inhibition of HERG channels stably expressed in a mammalian cell line by the antianginal agent perhexiline maleate. Br. J. Pharmacol. *127*, 243–251. https://doi.org/10.1038/sj.bjp.0702502.

23. Yan, M., Fan, P., Shi, Y., Feng, L., Wang, J., Zhan, G., and Li, B. (2016). Stereoselective Blockage of Quinidine and Quinine in the hERG Channel and the Effect of Their Rescue Potency on Drug-Induced hERG Trafficking Defect. Int. J. Mol. Sci. *17*, 1648. https://doi.org/10.3390/ijms17101648.

24. Paul, A.A., Witchel, H.J., and Hancox, J.C. (2002). Inhibition of the current of heterologously expressed HERG potassium channels by flecainide and comparison with quinidine, propafenone and lignocaine. Br. J. Pharmacol. *136*, 717–729. https://doi.org/10.1038/sj.bjp.0704784.

25. Perrin, M.J., Kuchel, P.W., Campbell, T.J., and Vandenberg, J.I. (2008). Drug binding to the inactivated state is necessary but not sufficient for high-affinity binding to human ether-à-go-go-related gene channels. Mol. Pharmacol. *74*, 1443–1452. https://doi.org/10.1124/mol.108.049056.

26. Bräu, M.E., Vogel, W., and Hempelmann, G. (1998). Fundamental properties of local anesthetics: half-maximal blocking concentrations for tonic block of Na+ and K+ channels in peripheral nerve. Anesth. Analg. *87*, 885–889. https://doi.org/10.1097/00000539-199810000-00026.

27. Poulin, H., Bruhova, I., Timour, Q., Theriault, O., Beaulieu, J.-M., Frassati, D., and Chahine, M. (2014). Fluoxetine blocks Nav1.5 channels via a mechanism similar to that of class 1 antiarrhythmics. Mol. Pharmacol. *86*, 378–389. https://doi.org/10.1124/mol.114.093104.

28. Liu, H., Atkins, J., and Kass, R.S. (2003). Common Molecular Determinants of Flecainide and Lidocaine Block of Heart Na+ Channels. J. Gen. Physiol. *121*, 199–214. https://doi.org/10.1085/jgp.20028723.

29. Bean, B.P., Cohen, C.J., and Tsien, R.W. (1983). Lidocaine block of cardiac sodium channels. J. Gen. Physiol. *81*, 613–642. https://doi.org/10.1085/jgp.81.5.613.

30. Kambouris, N.G., Nuss, H.B., Johns, D.C., Marbán, E., Tomaselli, G.F., and Balser, J.R. (2000). A revised view of cardiac sodium channel “blockade” in the long-QT syndrome. J. Clin. Invest. *105*, 1133–1140. https://doi.org/10.1172/JCI9212.

31. Wang, D.W., Mistry, A.M., Kahlig, K.M., Kearney, J.A., Xiang, J., and George, A.L. (2010). Propranolol Blocks Cardiac and Neuronal Voltage-Gated Sodium Channels. Front. Pharmacol. *1*, 144. https://doi.org/10.3389/fphar.2010.00144.

32. Li, Z., Jin, X., Wu, T., Huang, G., Wu, K., Lei, J., Pan, X., and Yan, N. (2021). Structural Basis for Pore Blockade of the Human Cardiac Sodium Channel Nav1.5 by the Antiarrhythmic Drug Quinidine. Angew. Chem. Int. Ed. *60*, 11474–11480. https://doi.org/10.1002/anie.202102196.

33. Crumb, W.J., Vicente, J., Johannesen, L., and Strauss, D.G. (2016). An evaluation of 30 clinical drugs against the comprehensive *in vitro* proarrhythmia assay (CiPA) proposed ion channel panel. J. Pharmacol. Toxicol. Methods *81*, 251–262. https://doi.org/10.1016/j.vascn.2016.03.009.

34. Finkel, A., Wittel, A., Yang, N., Handran, S., Hughes, J., and Costantin, J. (2006). Population Patch Clamp Improves Data Consistency and Success Rates in the Measurement of Ionic Currents. SLAS Discov. *11*, 488–496. https://doi.org/10.1177/1087057106288050.

35. O’Leary, M.E., and Chahine, M. (2024). Regulation of cardiac Nav1.5 channel gating and drug binding by the beta-1 subunit. Biophys. J. *123*, 110a–111a. https://doi.org/10.1016/j.bpj.2023.11.783.

36. Bankston, J.R., and Kass, R.S. (2010). Molecular determinants of local anesthetic action of beta-blocking drugs: Implications for therapeutic management of long QT syndrome variant 3. J. Mol. Cell. Cardiol. *48*, 246–253. https://doi.org/10.1016/j.yjmcc.2009.05.012.

37. Zhou, X.-Y., Hu, X.-X., Wang, C.-C., Lu, X.-R., Chen, Z., Liu, Q., Hu, G.-X., and Cai, J.-P. (2019). Enzymatic Activities of CYP3A4 Allelic Variants on Quinine 3-Hydroxylation In Vitro. Front. Pharmacol. *10*. https://doi.org/10.3389/fphar.2019.00591.

38. Usmani, K.A., Cho, T.M., Rose, R.L., and Hodgson, E. (2006). Inhibition of the Human Liver Microsomal and Human Cytochrome P450 1A2 and 3A4 Metabolism of Estradiol by Deployment-Related and Other Chemicals. Drug Metab. Dispos. *34*, 1606–1614. https://doi.org/10.1124/dmd.106.010439.

39. Zhang, F., Song, L., Wang, R., Zhao, B., Huang, J., Wu, L., Fan, Y., Lin, H., Jiang, Z., Yang, X., et al. (2025). Functional Imaging of CYP3A4 at Multiple Dimensions Using an AI-Driven High Performance Fluorogenic Substrate. Small Weinh. Bergstr. Ger. *21*, 2412178. https://doi.org/10.1002/smll.202412178.

40. Tang, P., Zheng, X., Hu, X., Yang, C., Chen, Z., Qian, J., Cai, J., and Hu, G. (2020). <p>Functional Measurement of CYP2C9 and CYP3A4 Allelic Polymorphism on Sildenafil Metabolism</p>. Drug Des. Devel. Ther. *14*, 5129–5141. https://doi.org/10.2147/DDDT.S268796.

41. Cocco, E., Javier Carmona, F., Razavi, P., Won, H.H., Cai, Y., Rossi, V., Chan, C., Cownie, J., Soong, J., Toska, E., et al. (2018). Neratinib is effective in breast tumors bearing both amplification and mutation of ERBB2 (HER2). Sci. Signal. *11*, eaat9773. https://doi.org/10.1126/scisignal.aat9773.

42. Harada, Y., Sato, A., Nakamura, H., Kai, K., Kitamura, S., Nakamura, T., Kurihara, Y., Ikeda, S., Sueoka, E., Kimura, S., et al. (2023). Anti-cancer effect of afatinib, dual inhibitor of HER2 and EGFR, on novel mutation HER2 E401G in models of patient-derived cancer. BMC Cancer *23*, 77. https://doi.org/10.1186/s12885-022-10428-3.

43. Schroeder, R.L., Stevens, C.L., and Sridhar, J. (2014). Small Molecule Tyrosine Kinase Inhibitors of ErbB2/HER2/Neu in the Treatment of Aggressive Breast Cancer. Molecules *19*, 15196–15212. https://doi.org/10.3390/molecules190915196.

44. Collins, D.M., Conlon, N.T., Kannan, S., Verma, C.S., Eli, L.D., Lalani, A.S., and Crown, J. (2019). Preclinical Characteristics of the Irreversible Pan-HER Kinase Inhibitor Neratinib Compared with Lapatinib: Implications for the Treatment of HER2-Positive and HER2-Mutated Breast Cancer. Cancers *11*, 737. https://doi.org/10.3390/cancers11060737.

45. Cai, X., Zhai, H.-X., Wang, J., Forrester, J., Qu, H., Yin, L., Lai, C.-J., Bao, R., and Qian, C. (2010). Discovery of 7-(4-(3-ethynylphenylamino)-7-methoxyquinazolin-6-yloxy)-N-hydroxyheptanamide (CUDc-101) as a potent multi-acting HDAC, EGFR, and HER2 inhibitor for the treatment of cancer. J. Med. Chem. *53*, 2000–2009. https://doi.org/10.1021/jm901453q.

46. Ashar, Y.V., Zhou, J., Gupta, P., Teng, Q.-X., Lei, Z.-N., Reznik, S.E., Lusvarghi, S., Wurpel, J., Ambudkar, S.V., and Chen, Z.-S. (2020). BMS-599626, a Highly Selective Pan-HER Kinase Inhibitor, Antagonizes ABCG2-Mediated Drug Resistance. Cancers *12*, 2502. https://doi.org/10.3390/cancers12092502.

47. Zoeller, R.A., and Geoghegan-Barek, K. (2019). A cell-based high-throughput screen identifies tyrphostin AG 879 as an inhibitor of animal cell phospholipid and fatty acid biosynthesis. Biochem. Biophys. Rep. *18*, 100621. https://doi.org/10.1016/j.bbrep.2019.100621.

48. Tang, P.C., Sun, L., and McMahon, G. (1998). 3-heteroaryl-2-indolinone compounds for the treatment of disease.

49. Irie, H., Ito, K., Fujioka, Y., Oguchi, K., Fujioka, A., Hashimoto, A., Ohsawa, H., Tanaka, K., Funabashi, K., Araki, H., et al. (2019). TAS0728, A Covalent-binding, HER2-selective Kinase Inhibitor Shows Potent Antitumor

Activity in Preclinical Models. Mol. Cancer Ther. *18*, 733–742. https://doi.org/10.1158/1535-7163.MCT-18-1085.

50. Son, J., Jang, J., Beyett, T.S., Eum, Y., Haikala, H.M., Verano, A., Lin, M., Hatcher, J.M., Kwiatkowski, N.P., Eser, P.Ö., et al. (2022). A Novel HER2-Selective Kinase Inhibitor Is Effective in HER2 Mutant and Amplified Non–Small Cell Lung Cancer. Cancer Res. *82*, 1633–1645. https://doi.org/10.1158/0008-5472.CAN-21-2693.
